# Dual Gene Delivery Reagents From Antiproliferative Alkylphospholipids for Combined Antitumor Therapy

**DOI:** 10.3389/fchem.2020.581260

**Published:** 2020-10-02

**Authors:** Boris Gaillard, Jean-Serge Remy, Françoise Pons, Luc Lebeau

**Affiliations:** Laboratoire de Conception et Application de Molécules Bioactives, UMR 7199 CNRS—Université de Strasbourg, Faculté de Pharmacie, Illkirch, France

**Keywords:** alkylphospholipid, miltefosine, perifosine, erufosine, hemolytic toxicity, prodrug, chemotherapy, gene therapy

## Abstract

Alkylphospholipids (APLs) have elicited great interest as antitumor agents due to their unique mode of action on cell membranes. However, their clinical applications have been limited so far by high hemolytic activity. Recently, cationic prodrugs of erufosine, a most promising APL, have been shown to mediate efficient intracellular gene delivery, while preserving the antiproliferative properties of the parent APL. Here, cationic prodrugs of the two APLs that are currently used in the clinic, miltefosine, and perifosine, are investigated and compared to the erufosine prodrugs. Their synthesis, stability, gene delivery and self-assembly properties, and hemolytic activity are discussed in detail. Finally, the potential of the pro-miltefosine and pro-perifosine compounds **M**_**E12**_ and **P**_**E12**_ in combined antitumor therapy is demonstrated using pUNO1-hTRAIL, a plasmid DNA encoding TRAIL, a member of the TNF superfamily. With these pro-APL compounds, we provide a proof of concept for a new promising strategy for cancer therapy combining gene therapy and APL-based chemotherapy.

## Introduction

**Figure 1:**
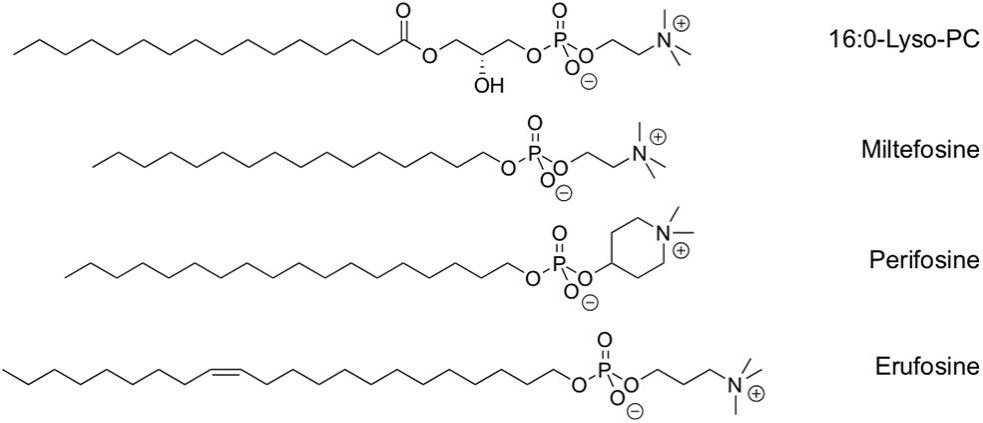
Chemical structure of lyso-PC and clinically relevant synthetic alkylphospholipids.

Alkylphospholipids (APLs) are metabolically stable analogs of lysophosphatidylcholines (lysoPCs) that constitute a new class of anticancer drugs with antiproliferative properties (de Almeida Pachioni et al., 2013; van Blitterswijk and Verheij, 2013; Markova et al., 2014; Jaffrès et al., 2016; Ríos-Marco et al., 2017). Due to their similarity with endogenous phospholipids, it is proposed they target the membrane lipid rafts and interfere with lipid homeostasis, thus altering lipid-linked signaling and inducing apoptosis. The unique way by which APLs can trigger cell apoptosis, through perturbation of the cell membranes, gives these compounds an advantage over conventional chemotherapeutic agents that interact with DNA. Furthermore, the action of APLs appears to be specific for tumor cells, and both cellular uptake and APL-induced apoptosis are increased in the malignant state of the cells (Kostadinova et al., 2015). Significant efforts have thus been made to synthesize metabolically stable analogs of lysoPCs with potential antineoplastic activity. Among these compounds, miltefosine and perifosine have been evaluated for their selective antitumor activity in phase I and II clinical trials against many types of advanced cancers (Figure 1). However, the clinical applications of these compounds still are limited, mainly due to gastrointestinal toxicity and high hemolytic activity, which are likely related to their high critical micellar concentration (CMC) that prevents their use as systemic agents. To date, the clinical use of miltefosine and perifosine has been essentially limited to the treatment of metastasis in breast cancer, through topical administration. More recently, erufosine and erucylphosphocholine were developed as next-generation APLs for systemic treatment of cancers. Substitution of the alkyl chain for an unsaturated 22-carbon chain resulted in lowered surface active properties and reduced hemolytic activity as tested at clinically relevant high doses, which was not feasible with previous APLs (Georgieva et al., 2002). However, none of these two drugs has reached the clinic yet.

As APLs have mechanisms of action that target various membrane signaling pathways involved in carcinogenesis (Ríos-Marco et al., 2017), they have been extensively investigated in combination with other chemotherapeutics (Bendell et al., 2011; Richardson et al., 2011; Fu et al., 2012; Gojo et al., 2013; Guidetti et al., 2014), and radiation therapy (Belka et al., 2004; Vink et al., 2007). Indeed, the use of combined treatments to fight against cancer is well-established (Gee et al., 2005). Monotherapies involve one single agent and aim at the suspension of one single signaling pathway. In contrast, tumor cells can grow through the initial oncogenic route or activate parallel signaling pathways (Lee et al., 2014). Combined therapies can simultaneously modulate more than one signaling pathway in tumor cells, with the consequence that the therapeutic effect can be maximized and, possibly, the resistance mechanisms can be overcome. Besides, a coordinate treatment with two drugs in separated carriers is associated with complex variations in pharmacokinetics and cannot ensure a proper co-localization of the drugs for synergistic action. This issue can be addressed by covalent conjugation of the drugs into hybrid molecules (Alam et al., 2018, 2019), or through their co-delivery in one single carrier (Tsouris et al., 2014). In this respect, the recommandation of therapies combining small-molecular drugs and nucleic acids has been endorsed in recent years for cancer treatment. Furthermore, such therapies are expected to help address the issues of genetic heterogeneity and existence of complicated signaling pathways (Huang et al., 2017). Noteworthy, only very few reports have yet appeared in the literature on the combined use of nucleic acids and APLs. In 2003, Zeisig *et al*. described the use of APLs as helper lipids in dimethyldioctadecyl ammonium bromide-based liposomal formulations of a LacZ reporter gene to improve intracellular gene delivery (Zeisig et al., 2003). The authors assumed that APLs may facilitate the transport of the lipoplexes through the cell membrane due to their “detergent-like properties.” In 2004, intratumoral co-injection of naked DNA with miltefosine has been reported (Settelen et al., 2004). While this non-condensing plasmid formulation failed to promote transgene expression *in vitro*, a reporter gene expression was increased by an order of magnitude *in vivo*. Another report published in 2007 describes the use of perifosine in combination with siRNA lipoplexes silencing c-FLIP, an inhibitory protein involved in the extrinsic pathway of apoptosis (Elrod et al., 2007). This is just about all that can be found in the literature on the joint use of nucleic acids and APLs.

As highly polar macromolecules, nucleic acids cannot diffuse through cell membranes and require the use of a delivery system for significant cell uptake. Various delivery strategies of nucleic acid based therapeutics have been developed, including viral (Lukashev and Zamyatnin, 2016) and non-viral (Shim et al., 2018) approaches. Although non-viral vectors can generally hardly compete with viral ones in terms of transfection efficiencies, they are superior to the latter with regard to production costs, payload size, and safety issues (Naldini, 2015). In non-viral delivery systems, convenient nucleic acid loading is usually accomplished through electrostatic interaction, and the type of non-viral vectors essentially includes cationic lipids and cationic polymers. In the course of our quest for nucleic acid carriers with improved transfection properties and safeness, we previously developed cationic derivatives of 1,2-dioleoyl-*sn*-glycero-3-phosphatidylcholine (DOPC), a major component of the cell membranes. These phosphotriester compounds proved highly efficient nucleic acid delivery reagents both *in vitro* (Pierrat et al., 2012, 2013a,b) and *in vivo* (Pierrat et al., 2016a,b,c). This inspired us to develop erufosine-based biolabile phosphotriesters (pro-APLs) as dual gene delivery reagents for combined cancer therapy (Gaillard et al., 2019). Our hypothesis was that the combination of a cationic biodegradable precursor of erufosine, as a nucleic acid carrier, and a DNA sequence encoding a pro-apoptotic protein, would diminish tumor cell survival as a result of both the expression of the transgene product and the *in situ* release of the antineoplastic APL upon carrier degradation. The results obtained indeed confirmed our hypothesis. As erufosine has not reached the clinic yet, we extended this new antitumor concept implementing gene therapy and chemotherapy to miltefosine and perifosine, the two APLs used clinically to date. We designed a series of 12 cationic prodrugs of miltefosine and perifosine which can regenerate the parent APL *in situ* under a chemical or enzyme stimulus. The properties of these APL prodrugs as gene delivery reagents have been investigated using a luciferase reporter gene assay, and the intrinsic cytotoxicity and hemolytic activity of the vectors have been determined. Finally, using a plasmid DNA encoding the tumor necrosis factor-related apoptosis-inducing ligand (TRAIL), the *in vitro* combined antiproliferative effect of TRAIL and APL prodrugs has been examined. For comparison purpose, results obtained with previously reported structure-related prodrugs of erufosine have been included and are discussed in this study.

## Materials and Methods

Detailed description of starting materials and standard procedures, and ^1^H-, ^13^C-, and ^31^P-NMR spectra are given as Supplementary Material.

### Synthesis of the Parent APLs

Dodecyl triflate, chloromethyl dodecanoate, 1-chloroethyl dodecanoate, chloromethyl dodecyl carbonate, 1-chloroethyl dodecyl carbonate, and chloromethyl oleyl carbonate were synthesized as described elsewhere (Heyes et al., 2002; Pierrat et al., 2013a).

*Miltefosine* Triethylamine (822 μL, 5.90 mmol) was added dropwise to phosphorus oxychloride (500 μL, 5.36 mmol) in anhydrous THF (20 mL) at 0°C under inert atmosphere. Then 1-hexadecanol (1.30 g, 5.36 mmol) in THF (10 mL) was added dropwise over a 30-min period and the reaction mixture was allowed to warm to rt. When all the alcohol had reacted (checked by TLC), temperature was brought back to 0°C and a second portion of triethylamine (3.3 mL, 21.4 mmol) was added followed by dropwise addition of 2-bromoethanol (380 μL, 5.36 mmol) in THF (10 mL). The reaction mixture was stirred at rt for 16 h before decomposition by addition of HCl 10% (10 mL) and heating at 40°C for 2 h. Solvent was removed under vacuum and the aqueous residue was extracted twice with CH_2_Cl_2_. The organic layer was dried over MgSO_4_, filtered and reduced under vacuum, and the crude residue was purified by flash chromatography (CH_2_Cl_2_/MeOH 10:0–8:2) to yield intermediate 2-bromoethyl hexadecyl hydrogenophosphate (1.01 g, 44%). *R*_f_: 0.43 (CH_2_Cl_2_/MeOH/H_2_O 75:22:3). ^1^H-NMR (400 MHz, CD_3_OD/CDCl_3_ 1:1) δ 0.85 (t, *J* = 6.7 Hz, 3H); 1.24 (m, 26H); 1.60 (tt, *J*_1_ = *J*_2_ = 6.4 Hz, 2H); 3.51 (t, *J* = 6.4 Hz; 2H); 3.86 (td, *J*_1_ = *J*_2_ = 6.5 Hz, 2H); 4.10 (t, *J*_1_ = *J*_2_ = 6.5 Hz, 2H). ^13^C-NMR (100.7 MHz, CD_3_OD/CDCl_3_ 1:1) δ 14.3; 23.3; 26.4; 30.0; 30.3 (br); 31.3; 32.6; 66.0; 66.7. ^31^P-NMR (162 MHz; CD_3_OD/CDCl_3_ 1:1) δ−0.56. IR ν 495; 596; 695; 733; 772; 879; 1,007; 1,218; 1,270; 1,456; 2,852; 2,921. The previous compound (1.0 g, 2.33 mmol) in CHCl_3_/CH_3_CN/*i*-PrOH 3:5:5 (13 mL) was treated with 45% (w/w) aqueous trimethylamine (5 mL, 34.3 mmol) at 70°C. When all starting material was consumed (checked by TLC), volatile was removed under vacuum and the residue was extracted twice with CHCl_3_/MeOH 1:3. The organic layer was washed with brine, reduced under vacuum, diluted with CHCl_3_, dried over MgSO_4_, filtered and evaporated. The crude residue was purified by flash chromatography (CH_2_Cl_2_/MeOH 75:22:3–45:45:10) to yield miltefosine (0.77 g, 81%). *R*_f_: 0.1 (CH_2_Cl_2_/MeOH/H_2_O 75:22:3). ^1^H-NMR (400 MHz, CD_3_OD/CDCl_3_ 1:1) δ 0.85 (t, *J* = 6.2 Hz, 3H); 1.24 (m, 26H); 1.61 (tt, *J*_1_ = *J*_2_ = 6.4 Hz, 2H); 3.19 (s, 9H); 3.58 (m, 2H); 3.83 (td, *J*_1_ = *J*_2_ = 6.5 Hz, 2H); 4.20 (m, 2H). ^13^C-NMR (100.7 MHz, CD_3_OD/CDCl_3_ 1:1) δ 14.3; 23.2; 26.4; 30.1 (2C); 30.3 (8C); 31.3; 32.5; 54.5; 59.7; 66.7; 67.0. ^31^P-NMR (162 MHz, CD_3_OD/CDCl_3_ 1:1) δ−0.32. IR (ATR) ν 497; 716; 746; 853; 927; 957; 1,050; 1,126; 1,246; 1,471; 1,633; 2,849; 2,914; 3,372. HR-MS (ESI+) m/z [M+H]^+^ calcd for C_21_H_47_NO_4_P^+^ 408.3237, found 408.3239.

*Perifosine* Phosphorus oxychloride (864 μL, 9.2 mmol) was added to 1-hexadecanol (2.50 g, 9.2 mmol) in anhydrous Et_2_O (100 mL) at 0°C, followed by dropwise addition of triethylamine (1.29 mL, 9.2 mmol). The reaction mixture was stirred at rt for 16 h and filtered, and the filtrate was reduced under vacuum. The dry residue was dissolved in anhydrous CHCl_3_ (100 mL) and *N,N*-dimethyl-4-hydroxypiperidinium tosylate (2.78 g, 9.24 mmol), triethylamine (3.00 mL, 21.5 mmol), and 4-DMAP (65 mg, 0.53 mmol) in CHCl_3_ (100 mL) were introduced in the reaction flask and allowed to react for 2 d at rt. The reaction mixture was then reduced under vacuum, suspended in THF (100 mL) and refluxed with H_2_O (2 mL) for 6 h. The crude mixture was reduced under vacuum and the residue was directly purified by flash chromatography (CHCl_3_/MeOH/NH_4_OH 10:6:1) to yield perifosine (0.70 g, 16%). *R*_f_: 0.2 (CH_2_Cl_2_/MeOH/H_2_O 75:22:3). ^1^H-NMR (400 MHz, CD_3_OD/CDCl_3_ 1:1) δ 0.85 (t, *J* = 6.5 Hz, 3H); 1.24 (m, 30H); 1.59 (m, 2H); 2.10 (m, 4H); 3.09 (s, 3H); 3.16 (s, 3H); 3.25-3.53 (m, 4H); 3.82 (m, 2H); 4.40 (m, 1H). ^13^C-NMR (100.7 MHz, CD_3_OD/CDCl_3_ 1:1) δ 14.3; 23.2; 26.4; 27.2 (2C); 29.9; 30.0; 30.2 (10C); 31.5; 32.5; 55.4 (2C); 59.2 (2C); 65.0; 66.6. ^31^P-NMR (162 MHz, CD_3_OD/CDCl_3_ 1:1) δ−0.40. IR ν 479; 720; 846; 919; 994; 1,062; 1,124; 1,156; 1,225; 1,468; 2,848; 2,916. HR-MS (ESI+) m/z [M+Na]^+^ calcd for C_25_H_52_NNaO_4_P^+^ 484.3526, found 484.3528.

### Synthesis of the Pro-APLs

*General Procedure* The APL (0.5 mmol) and electrophilic reagent (4.0 mmol) were reacted in refluxing anhydrous CHCl_3_ (12 mL) for 24 h with stirring under inert atmosphere. Solvent was removed *in vacuo* and the crude residue was purified by flash chromatography (CH_2_Cl_2_/MeOH 10:0–7:3) to yield the corresponding pro-APL.

*2-(((Dodecyloxy)(hexadecyloxy)phosphoryl)oxy)-N,N,N-trimethylethan-1-aminium triflate (****M***_***12***_*)*. This compound (101 mg, 84%) was obtained from miltefosine (80 mg, 0.20 mmol) and dodecyl triflate (162 mg, 0.53 mmol) according to the general procedure except the reaction was conducted at rt. *R*_f_: 0.7 (CH_2_Cl_2_/MeOH/H_2_O 75:22:3). ^1^H-NMR (400 MHz, CDCl_3_) δ 0.88 (t, *J* = 6.6 Hz, 6H); 1.26 (m, 44H); 1.68 (tt, *J*_1_ = *J*_2_ = 6.8 Hz, 4H); 3.31 (s, 9H); 3.83 (m; 2H); 4.08 (m, 4H); 4.48 (m, 2H). ^13^C-NMR (100.7 MHz, CDCl_3_) δ 14.3 (2C); 22.9 (2C); 25.6 (2C); 29.4 (2C); 29.6 (2C); 29.7 (2C); 29.8 (4C); 29.9 (6C); 30.5 (2C); 32.1 (2C); 54.6 (3C); 61.2; 65.9; 69.2 (2C). ^31^P-NMR (162 MHz, CDCl_3_) δ−2.14. IR ν 517; 574; 638; 1,030; 1,161; 1,226; 1,253; 1,467; 2,852; 2,921; 3,500. HR-MS (ESI+) m/z [M-Cl]^+^ calcd for C_33_H_71_NO_4_P^+^ 576.5115, found 576.5108.

*2-((((Dodecanoyloxy)methoxy)(hexadecyloxy)phosphoryl)oxy)-N,N,N-trimethylethan-1-aminium chloride (****M***_***E12***_*)*. This compound (136 mg, 42%) was obtained from miltefosine (200 mg, 0.49 mmol) and chloromethyl dodecanoate (979 mg, 3.94 mmol) according to the general procedure. *R*_f_: 0.5 (CH_2_Cl_2_/MeOH/H_2_O 75:22:3). ^1^H-NMR (400 MHz, CD_3_OD/CDCl_3_ 1:1) δ 0.85 (t, *J* = 6.3 Hz, 6H); 1.24 (m, 42H); 1.63 (tt, *J*_1_ = *J*_2_ = 6.8 Hz, 2H); 1.69 (tt, *J*_1_ = *J*_2_ = 6.6 Hz, 2H); 2.40 (t, *J* = 7.5 Hz, 2H); 3.23 (s, 9H); 3.76 (m, 2H); 4.11 (td, *J*_1_ = *J*_2_ = 6.3 Hz, 2H); 4.50 (m, 2H); 5.64 (ABX syst., *J*_*AB*_ = 5.2 Hz, *J*_*AX*_ = 13.4 Hz, *J*_*BX*_ = 11.4 Hz, 2H). ^13^C-NMR (100.7 MHz, CD_3_OD/CDCl_3_ 1:1) δ 14.5 (2C); 23.3 (2C); 25.1; 25.9; 29.7; 29.8; 29.9; 30.0 (2C); 30.1 (4C); 30.2 (7C); 30.7; 32.5; 34.6; 54.7 (3C); 62.3; 66.5; 70.2; 84.2; 173.2. ^31^P-NMR (162 MHz, CD_3_OD/CDCl_3_ 1:1) δ−3.80. IR ν 458; 490; 721; 832; 873; 965; 1,042; 1,159; 1,264; 1,468; 1,760; 2,849; 2,917; 2,956; 3,382. HR-MS (ESI+) m/z [M-Cl]^+^ calcd for C_34_H_71_NO_6_P^+^ 620.5014, found 620.5004.

*2-(((1-(Dodecanoyloxy)ethoxy)(hexadecyloxy)phosphoryl)oxy)-N,N,N-trimethylethan-1-aminium chloride (****M***${\;}_{{\text{E}}^{\prime }12}$*)*. This compound (169 mg, 51%) was obtained as two separated couples of enantiomers (E1 and E2) from miltefosine (201 mg, 0.49 mmol) and 1-chloroethyl dodecanoate (1.04 g, 3.95 mmol) according to the general procedure. *R*_f_: 0.5 (E1) and 0.4 (E2) (CH_2_Cl_2_/MeOH/H_2_O 75:22:3). ^1^H-NMR (400 MHz, CD_3_OD/CDCl_3_ 1:1) E1 δ 0.85 (t, *J* = 6.1 Hz, 6H); 1.24 (m, 42H); 1.58 (d, *J* = 5.3 Hz, 3H); 1.61 (tt, *J*_1_ = *J*_2_ = 6.8 Hz, 2H); 1.69 (tt, *J*_1_ = *J*_2_ = 6.6 Hz, 2H); 2.37 (t, *J* = 7.2 Hz, 2H); 3.24 (s, 9H); 3.76 (m, 2H); 4.09 (td, *J*_1_ = *J*_2_ = 6.7 Hz, 2H); 4.50 (m, 2H); 6.42 (qd, *J*_1_ = *J*_2_ = 5.3 Hz, 1H). ^1^H-NMR (400 MHz, CD_3_OD/CDCl_3_ 1:1) E2 δ 0.86 (t, *J* = 6.2 Hz, 6H); 1.24 (m, 42H); 1.56 (d, *J* = 5.3 Hz, 3H); 1.61 (tt, *J*_1_ = *J*_2_ = 6.8 Hz, 2H); 1.68 (tt, *J*_1_ = *J*_2_ = 6.6 Hz, 2H); 2.37 (t, *J* = 7.3 Hz, 2H); 3.24 (s, 9H); 3.76 (m, 2H); 4.09 (td, *J*_1_ = *J*_2_ = 6.3 Hz, 2H); 4.50 (m, 2H); 6.42 (qd, *J*_1_ = *J*_2_ = 5.3 Hz, 2H). ^13^C-NMR (100.7 MHz, CD_3_OD/CDCl_3_ 1:1) E1 δ 14.5 (2C); 21.7; 23.4 (2C); 25.3; 26.1; 29.6; 29.7; 29.8; 29.9; 30.0 (2C); 30.1 (2C); 30.2 (4C); 30.3 (4C); 30.7; 32.5 (2C); 34.5; 54.6 (3C); 62.3; 66.5; 70.0; 92.4; 173.3. ^13^C-NMR (100.7 MHz, CD_3_OD/CDCl_3_ 1:1) E2 δ 14.5 (2C); 21.6; 23.4 (2C); 25.3; 26.1; 29.6; 29.7; 29.8; 29.9; 30.0 (2C); 30.1 (2C); 30.2 (4C); 30.3 (4C); 30.7; 32.5 (2C); 34.5; 54.6 (3C); 62.1; 66.5; 70.2; 92.3; 172.8. ^31^P-NMR (162 MHz, CD_3_OD/CDCl_3_ 1:1) E1 δ−5.96. ^31^P-NMR (162 MHz, CD_3_OD/CDCl_3_ 1:1) E2 δ−5.57. IR ν 509; 721; 934; 976; 1,050; 1,082; 1,165; 1,238; 1,267; 1,467; 1,755; 2,849; 2,916; 2,956; 3,389. HR-MS (ESI+) m/z [M-Cl]^+^ calcd for C_35_H_73_NO_6_P^+^ 634.5170, found 634.5167. Note: IR absorption and HR-MS were measured on the mixture of the four diastereomers.

*3-((((((Dodecyloxy)carbonyl)oxy)methoxy)(hexadecyloxy)phosphoryl)oxy)-N,N,N-trimethylpropan-1- aminium chloride (****M***_***C12***_*)*. This compound (86 mg, 26%) was obtained from miltefosine (200 mg, 0.49 mmol) and chloromethyl dodecyl carbonate (1.06 g, 3.78 mmol) according to the general procedure. *R*_f_: 0.55 (CH_2_Cl_2_/MeOH/H_2_O 75:22:3). ^1^H-NMR (400 MHz, CD_3_OD/CDCl_3_ 1:1) δ 0.85 (t, *J* = 6.3 Hz, 6H); 1.24 (m, 44H); 1.69 (m, 4H); 3.23 (s, 9H); 3.77 (m, 2H); 4.13 (td, *J*_1_ = *J*_2_ = 6.9 Hz, 2H); 4.19 (t, *J* = 6.7 Hz, 2H); 4.52 (m, 2H); 5.66 (m, 2H). ^13^C-NMR (100.7 MHz, CD_3_OD/CDCl_3_ 1:1) δ 14.5 (2C); 23.2 (2C); 25.9; 26.2; 29.1; 29.7; 29.8; 29.9 (2C); 30.1 (2C); 30.2 (5C); 30.3 (5C); 30.7; 32.5 (2C); 54.5 (3C); 62.2; 66.2; 70.0; 70.3; 86.6; 154.5. ^31^P-NMR (162 MHz, CD_3_OD/CDCl_3_ 1:1) δ −3.58. IR ν 670; 771; 950; 1,042; 1,266; 1,468; 1,762; 2,850; 2,918; 3,378. HR-MS (ESI+) m/z [M-Cl]^+^ calcd for C_35_H_73_NO_7_P^+^ 650.5119, found 650.5135.

*3-((((((Dodecyloxy)carbonyl)oxy)ethoxy)(hexadecyloxy)phosphoryl)oxy)-N,N,N-trimethylpropan-1-aminium chloride (****M***${\;}_{{\text{C}}^{\prime }12}$*)*. This compound (201 mg, 59%) was obtained as a mixture of diastereomers from miltefosine (200 mg, 0.49 mmol) and 1-chloroethyl dodecyl carbonate (1.16 g, 3.97 mmol) according to the general procedure. *R*_f_: 0.5 and 0.6 (CH_2_Cl_2_/MeOH/H_2_O 75:22:3). ^1^H-NMR (400 MHz, CD_3_OD/CDCl_3_ 1:1) δ 0.86 (t, *J* = 6.3 Hz, 6H); 1.24 (m, 44H); 1.60 (d, *J* = 5.2 Hz, 3H); 1.68 (m, 4H); 3.23 (s, 9H); 3.75 (m, 2H); 4.17 (m, 4H); 4.49 (m, 2H); 6.29–6.38 (qd, m, 1H). ^13^C-NMR (100.7 MHz, CD_3_OD/CDCl_3_ 1:1) δ 14.3 (2C); 21.5; 23.2 (2C); 25.9; 26.2; 29.1; 29.9; 30.0 (2C); 30.1 (2C); 30.2 (5C); 30.3 (6C); 30.7; 32.5 (2C); 54.5 (3C); 62.2; 66.3; 69.7; 70.0; 95.8; 154.0. ^31^P-NMR (162 MHz, CD_3_OD/CDCl_3_ 1:1) δ−5.63; −5.95. IR ν 458; 490; 721; 832; 873; 965; 1,042; 1,159; 1,264; 1,468; 1,760; 2,849; 2,917; 2,956; 3,382. HR-MS (ESI+) m/z [M-Cl]^+^ calcd for C_34_H_71_NO_6_P^+^ 620.5014, found 620.5004.

*3-((((((Dodecyloxy)carbonyl)oxy)methoxy)(oleyloxy)phosphoryl)oxy)-N,N,N-trimethylpropan-1-aminium chloride (****M***_*****C18:1*****_*)*. This compound (60 mg, 16%) was obtained from miltefosine (201 mg, 0.49 mmol) and chloromethyl oleyl carbonate (1.42 g, 3.92 mmol) according to the general procedure. *R*_f_: 0.5 (CH_2_Cl_2_/MeOH/H_2_O 75:22:3). ^1^H-NMR (400 MHz, CD_3_OD/CDCl_3_ 1:1) δ 0.86 (t, *J* = 6.1 Hz, 6H); 1.24 (m, 48H); 1.68 (m, 4H); 2.00 (m, 4H); 3.24 (s, 9H); 3.79 (m, 2H); 4.12 (td, *J*_1_ = *J*_2_ = 6.7 Hz, 2H); 4.19 (t, *J* = 6.5 Hz, 2H); 4.52 (m, 2H); 5.32 (m, 2H); 5.65 (m, 2H). ^13^C-NMR (100.7 MHz, CD_3_OD/CDCl_3_ 1:1) δ 14.3 (2C); 23.2 (2C); 25.9; 26.2; 27.7 (2C); 29.1; 29.7 (2C); 29.8; 30.1 (2C); 30.2; 30.3 (3C); 30.4 (9C); 30.7; 32.5 (2C); 54.4 (3C); 62.1; 66.2; 70.0; 70.2; 86.5; 130.2; 130.5; 154.3. ^31^P-NMR (162 MHz, CD_3_OD/CDCl_3_ 1:1) δ−3.57. IR ν 486; 721; 786; 836; 951; 977; 1,022; 1,049; 1,156; 1,258; 1,417; 1,467; 1,758; 2,850; 2,919; 2,956; 3,390. HR-MS (ESI+) m/z [M-Cl]^+^ calcd for C_41_H_83_NO_7_P^+^ 732.5902, found 732.5905.

*4-(((Dodecyloxy)(octadecyloxy)phosphoryl)oxy)-1,1-dimethylpiperidin-1-ium chloride (****P***_*****12*****_*)*. This compound (55 mg, 48%) was obtained from perifosine (79 mg, 0.17 mmol) and dodecyl triflate (141 mg, 0.44 mmol) according to the general procedure except the reaction was conducted at rt. *R*_f_: 0.68 (CH_2_Cl_2_/MeOH/H_2_O 75:22:3). ^1^H-NMR (400 MHz, CD_3_OD/CDCl_3_ 1:1) δ 0.86 (t, *J* = 6.7 Hz, 6H); 1.24 (m, 48H); 1.68 (tt, *J*_1_ = *J*_2_ = 7.0 Hz, 4H); 2.10-2.32 (m, 4H); 3.14 (s, 3H); 3.20 (s, 3H); 3.41-3.55 (m, 4H); 4.06 (m, 4H); 4.70 (m, 1H). ^13^C-NMR (100.7 MHz; CD_3_OD/CDCl_3_ 1:1) δ 14.4 (2C); 23.3 (2C); 26.1 (2C); 27.1 (2C); 29.6 (2C); 29.8 (2C); 30.0 (2C); 30.1 (5C); 30.2 (6C); 30.3; 30.6 (2C); 32.6 (2C); 54.1 (2C); 58.9 (2C); 69.3; 69.4 (2C). ^31^P-NMR (162 MHz, CD_3_OD/CDCl_3_ 1:1) δ−2.07. IR ν 516; 573; 638; 721; 764; 923; 1,029; 1,161; 1,245; 1,467; 2,851; 2,920; 3,252. HR-MS (ESI+) m/z [M-Cl]^+^ calcd for C_37_H_77_NO_4_P^+^ 630.5585, found 630.5581.

*4-((((Dodecanoyloxy)methoxy)(octadecyloxy)phosphoryl)oxy)-1,1-dimethylpiperidin-1-ium chloride (****P***_***E12***_*)*. This compound (51 mg, 22%) was obtained from perifosine (150 mg, 0.33 mmol) and chloromethyl dodecanoate (675 mg, 2.70 mmol) according to the general procedure. *R*_f_: 0.6 (CH_2_Cl_2_/MeOH/H_2_O 75:22:3). ^1^H-NMR (400 MHz, CD_3_OD/CDCl_3_ 1:1) δ 0.86 (t, *J* = 6.7 Hz, 6H); 1.24 (m, 46H); 1.63 (tt, *J*_1_ = *J*_2_ = 6.9 Hz, 2H); 1.68 (tt, *J*_1_ = *J*_2_ = 7.2 Hz, 2H); 2.10–2.32 (m, 4H); 2.39 (t, *J* = 7.6 Hz, 2H); 3.17 (s, 3H); 3.22 (s, 3H); 3.45–3.59 (m, 4H); 4.08 (td, *J*_1_ = *J*_2_ = 6.8 Hz, 2H); 4.70 (m, 1H); 5.62 (ABX syst., *J*_*AB*_ = 5.2 Hz, *J*_*AX*_ = 13.8 Hz, *J*_*BX*_ = 11.1 Hz, 2H). ^13^C-NMR (100.7 MHz, CD_3_OD/CDCl_3_ 1:1) δ 14.5 (2C); 23.4 (2C); 25.3; 26.1; 27.1 (2C); 29.8; 29.9; 30.0; 30.1 (2C); 30.2; 30.3 (5C); 30.4 (7C); 30.9; 32.6 (2C); 34.6; 54.7 (2C); 58.9 (2C); 69.9; 70.0; 83.5; 173.1. ^31^P-NMR (162 MHz, CD_3_OD/CDCl_3_ 1:1) δ−4.22. IR ν 531; 666; 720; 760; 802; 923; 966; 1,020; 1,148; 1,264; 1,468; 1,644; 1,764; 2,849; 2,916; 2,955; 3,378. HR-MS (ESI+) m/z [M-Cl]^+^ calcd for C_38_H_77_NO_6_P^+^ 674.5483, found 674.5477.

*4-(((1-(Dodecanoyloxy)ethoxy)(octadecyloxy)phosphoryl)oxy)-1,1-dimethylpiperidin-1-ium chloride (****P***${\;}_{{\text{E}}^{\prime }12}$*)*. This compound (96 mg, 20%) was obtained as two separated couples of enantiomers (E1 and E2) from perifosine (300 mg, 0.65 mmol) and 1-chloroethyl dodecanoate (1.33 g, 5.06 mmol) according to the general procedure. *R*_f_: 0.3 (E1) and 0.4 (E2) (CH_2_Cl_2_/MeOH/H_2_O 75:22:3). ^1^H-NMR (400 MHz, CD_3_OD/CDCl_3_ 1:1) D1 δ 0.86 (t, *J* = 6.7 Hz, 6H); 1.24 (m, 46H); 1.56 (d, *J* = 5.2 Hz, 3H); 1.61 (tt, *J*_1_ = *J*_2_ = 6.9 Hz, 2H); 1.68 (tt, *J*_1_ = *J*_2_ = 7.2 Hz, 2H); 2.08–2.30 (m, 4H); 2.36 (t, *J* = 7.6 Hz, 2H); 3.17 (s, 3H); 3.24 (s, 3H); 3.45–3.59 (m, 4H); 4.06 (td, *J*_1_ = *J*_2_ = 6.8 Hz, 2H); 4.70 (m, 1H); 6.46 (qd, *J*_1_ = *J*_2_ = 5.2 Hz, 1H). ^1^H-NMR (400 MHz, CD_3_OD/CDCl_3_ 1:1) E2 δ 0.86 (t, *J* = 6.7 Hz, 6H); 1.24 (m, 46H); 1.54 (d, *J* = 5.2 Hz, 3H); 1.61 (tt, *J*_1_ = *J*_2_ = 6.9 Hz, 2H); 1.68 (tt, *J*_1_ = *J*_2_ = 7.2 Hz, 2H); 2.08–2.30 (m, 4H); 2.35 (t, *J* = 7.6 Hz, 2H); 3.18 (s, 3H); 3.24 (s, 3H); 3.45–3.59 (m, 4H); 4.08 (td, *J*_1_ = *J*_2_ = 6.8 Hz, 2H); 4.70 (m, 1H); 6.50 (qd, *J*_1_ = *J*_2_ = 5.2 Hz, 1H).^13^C-NMR (100.7 MHz, CD_3_OD/CDCl_3_ 1:1) E1 δ 14.5 (2C); 21.7; 23.4 (2C); 25.3; 26.1; 26.8; 27.1; 29.7; 29.8; 30.0; 30.1 (2C); 30.2; 30.3 (5C); 30.4 (7C); 30.8; 32.6 (2C); 34.7; 55.2 (2C); 58.6 (2C); 69.5; 69.7; 92.1; 173.1. ^13^C-NMR (100.7 MHz, CD_3_OD/CDCl_3_ 1:1) E2 δ 14.5 (2C); 21.7; 23.4 (2C); 25.3; 26.1; 27.0; 27.1; 29.7; 29.8; 30.0; 30.1 (2C); 30.2; 30.3 (5C); 30.4 (7C); 30.8; 32.6 (2C); 34.7; 54.8 (2C); 58.8 (2C); 69.7; 69.9; 92.3; 172.8. ^31^P-NMR (162 MHz, CD_3_OD/CDCl_3_ 1:1) E1 δ−6.1. ^31^P-NMR (162 MHz, CD_3_OD/CDCl_3_ 1:1) E2 δ−6.0. IR ν 553; 638; 772; 752; 974; 1,012; 1,090; 1,163; 1,263; 1,466; 1,745; 2,851; 2,920; 3,380. HR-MS (ESI+) m/z [M-Cl]^+^ calcd for C_39_H_79_NO_6_P^+^ 688.5640, found 688.5627. Note: IR absorption and HR-MS were measured on the mixture of the four diastereomers.

*3-((((((Dodecyloxy)carbonyl)oxy)methoxy)(octadecyloxy)phosphoryl)oxy)-N,N,N-trimethylpropan-1-aminium chloride (****P***_***C12***_*)*. This compound (53 mg, 22%) was obtained from perifosine (152 mg, 0.33 mmol) and chloromethyl dodecyl carbonate (738 mg, 2.65 mmol) according to the general procedure. *R*_f_: 0.55 (CH_2_Cl_2_/MeOH/H_2_O 75:22:3). ^1^H-NMR (400 MHz, CDCl_3_) δ 0.86 (t, *J* = 6.8 Hz, 6H); 1.24 (m, 48H); 1.68 (m, 4H); 2.10–2.34 (m, 4H); 3.53 (s, 3H); 3.66 (s, 3H); 3.67–3.96 (m, 4H); 4.10 (td, *J*_1_ = *J*_2_ = 6.8 Hz, 2H); 4.19 (t, *J* = 6.8 Hz, 2H); 4.80 (m, 1H); 5.65 (ABX syst., *J*_*AB*_ = 5.6 Hz, *J*_*AX*_ = 11.6 Hz, *J*_*BX*_ = 10.4 Hz, 2H). ^13^C-NMR (100.7 MHz, CD_3_OD/CDCl_3_ 1:1) δ 14.4 (2C); 23.3 (2C); 26.1; 26.4; 27.1 (2C); 29.2; 29.8 (2C); 29.9 (2C); 30.2 (4C); 30.4 (10C); 30.9; 32.6 (2C); 55.7 (2C); 58.8 (2C); 69.6; 69.8; 70.1; 86.5; 154.4. ^31^P-NMR (162 MHz, CD_3_OD/CDCl_3_ 1:1) δ−3.82. IR ν 482; 529; 665; 720; 781; 850; 922; 944; 979; 1,019; 1,165; 1,266; 1,427; 1,465; 1,761; 2,848; 2,916; 2,956; 3,492. HR-MS (ESI+) m/z [M-Cl]^+^ calcd for C_39_H_79_NO_7_P^+^ 704.5589, found 704.5588.

*3-((((((Dodecyloxy)carbonyl)oxy)ethoxy)(octadecyloxy)phosphoryl)oxy)-N,N,N-trimethylpropan-1-aminium chloride (****P***${\;}_{{\text{C}}^{\prime }\text{12}}$*)*. This compound (38 mg, 16%) was obtained as two separated couples of enantiomers (E1 and E2) from perifosine (149 mg, 0.32 mmol) and 1-chloroethyl dodecyl carbonate (762 mg, 2.60 mmol) according to the general procedure. *R*_f_: 0.4 (E1) and 0.5 (E2) (CH_2_Cl_2_/MeOH/H_2_O 75:22:3). ^1^H-NMR (400 MHz, CD_3_OD/CDCl_3_ 1:1) E1 δ 0.86 (t, *J* = 6.8 Hz, 6H); 1.24 (m, 48H); 1.60 (d, *J* = 5.3 Hz, 3H); 1.63 (tt, *J*_1_ = *J*_2_ = 6.9 Hz, 2H); 1.68 (tt, *J*_1_ = *J*_2_ = 6.4 Hz, 2H); 2.10–2.34 (m, 4H); 3.15 (s, 3H); 3.22 (s, 3H) 3.41–3.65 (m, 4H); 4.10 (td, *J*_1_ = *J*_2_ = 6.8 Hz, 2H); 4.19 (t, *J* = 6.8 Hz, 2H); 4.72 (m, 1H); 6.33 (m, 1H). ^1^H-NMR (400 MHz, CD_3_OD/CDCl_3_ 1:1) E2 δ 0.86 (t, *J* = 6.8 Hz, 6H); 1.24 (m, 48H); 1.58 (d, *J* = 5.3 Hz, 3H); 1.63 (tt, *J*_1_ = *J*_2_ = 6.9 Hz, 2H); 1.68 (tt, *J*_1_ = *J*_2_ = 6.4 Hz, 2H); 2.10–2.34 (m, 4H); 3.17 (s, 3H); 3.24 (s, 3H); 3.41–3.65 (m, 4H); 4.10 (td, *J*_1_ = *J*_2_ = 6.8 Hz, 2H); 4.19(t, *J* = 6.8 Hz, 2H); 4.72 (m, 1H); 6.35 (m, 1H). ^13^C-NMR (100.7 MHz, CD_3_OD/CDCl_3_ 1:1) E1 δ 14.6 (2C); 21.9; 23.3 (2C); 25.9; 26.2; 27.1; 29.1; 29.7; 29.8; 29.9 (2C); 30.2 (4C); 30.3 (4C); 30.4 (6C); 30.9; 32.6 (2C); 55.7 (2C); 58.3 (2C); 68.9; 69.3; 69.6; 95.1; 153.8. ^13^C-NMR (100.7 MHz, CD_3_OD/CDCl_3_ 1:1) E2 δ 14.6 (2C); 22.0; 23.3 (2C); 25.9; 26.2; 27.1 (2C); 29.1; 29.8 (2C); 29.9 (2C); 30.2 (4C); 30.3 (5C); 30.4 (5C); 30.9; 32.6 (2C); 55.7 (2C); 58.3 (2C); 68.9; 69.3; 69.6; 95.2; 153.8. ^31^P-NMR (162 MHz, CD_3_OD/CDCl_3_ 1:1) E1 δ−6.13. ^31^P-NMR (162 MHz, CD_3_OD/CDCl_3_ 1:1) E2 δ−5.93. IR ν 534; 720; 772; 924; 971; 1,023; 1,152; 1,270; 1,396; 1,467; 2,849; 2,916; 2,955; 3,388. HR-MS (ESI+) m/z [M-Cl]^+^ calcd for C_40_H_81_NO_7_P^+^ 718.5745, found 718.5744. Note: IR and HR-MS were measured on the mixture of the four diastereomers.

*3-((((((Dodecyloxy)carbonyl)oxy)methoxy)(oleyloxy)phosphoryl)oxy)-N,N,N-trimethylpropan-1-aminium chloride (****P***_***C18:1***_*)*. This compound (40 mg, 15%) was obtained from perifosine (148 mg, 0.32 mmol) and chloromethyl oleyl carbonate (930 mg, 2.58 mmol) according to the general procedure. *R*_f_: 0.55 (CH_2_Cl_2_/MeOH/H_2_O 75:22:3). ^1^H-NMR (400 MHz, CD_3_OD/CDCl_3_ 1:1) δ 0.86 (t, *J* = 6.8 Hz, 6H); 1.24 (m, 52H); 1.68 (m, 4H); 2.00 (m, 4H); 2.10–2.34 (m, 4H); 3.10 (s, 3H); 3.19 (s, 3H); 3.45–3.59 (m, 2H); 4.10 (td, *J*_1_ = *J*_2_ = 6.8 Hz, 2H); 4.19 (t, *J* = 6.8 Hz, 2H); 4.72 (m, 1H); 5.31 (m, 2H); 5.65 (m, 2H). ^13^C-NMR (100.7 MHz, CD_3_OD/CDCl_3_ 1:1) δ 14.5 (2C); 23.3 (2C); 26.4; 26.5; 27.0 (2C); 27.8 (2C); 29.2; 30.0 (2C); 30.1; 30.2; 30.3; 30.4 (15C); 30.9; 32.6 (2C) 54.7 (2C); 58.8 (2C); 70.0; 70.1; 70.2; 86.6; 130.4 (2C); 155.2. ^31^P-NMR (162 MHz, CD_3_OD/CDCl_3_ 1:1) δ−4.10. IR ν 516; 720; 968; 1,027; 1,251; 1,465; 1,646; 1,764; 2,851; 2,919; 3,387. HR-MS (ESI+) m/z [M-Cl]^+^ calcd for C_45_H_89_NO_7_P^+^ 786.6371, found 786.6355.

## Results and Discussion

### Synthesis

As zwitterionic phosphodiesters, APLs can be conveniently transformed into cationic lipids with potential nucleic acid condensing properties, by straightforward esterification of their phosphate group. The net positive charge generated in the resulting phosphotriester molecules can thus establish electrostatic interactions with anionic phosphates in nucleic acids. This results in the formation of complexes with a nanometric size ready for cell uptake through the endocytic route (Rehman et al., 2013). Besides, when the chemical transformation is reversible and sensitive, e.g., to a pH or enzyme stimulus as met along the endo-lysosome pathway, the zwitterionic parent APLs can be regenerated *in situ*, and this is expected to have cascading effect. Firstly, due to their membrane-active properties, the APLs can disrupt the endosome membrane, hence facilitating escape of the genetic material into the cytosol. Secondly, as the newly unmasked negative charge on the APLs favorably competes with that of the phosphates of nucleic acids, decomplexation of the latters occurs, thus triggering decondensation of the transfection particles that favors proper processing of the transgene by the cell translation machinery. Finally, as most amphiphilic molecules enter the endocytic recycling pathway from the sorting endosomes through a highly dynamic and effective process (Mukherjee et al., 1999; Juliano, 2018), the *in situ* regenerated APL molecules are intended to traffic to the plasma membrane, where they are expected to operate their intrinsic apoptotic activity (Gaillard et al., 2020).

**Figure 2:**
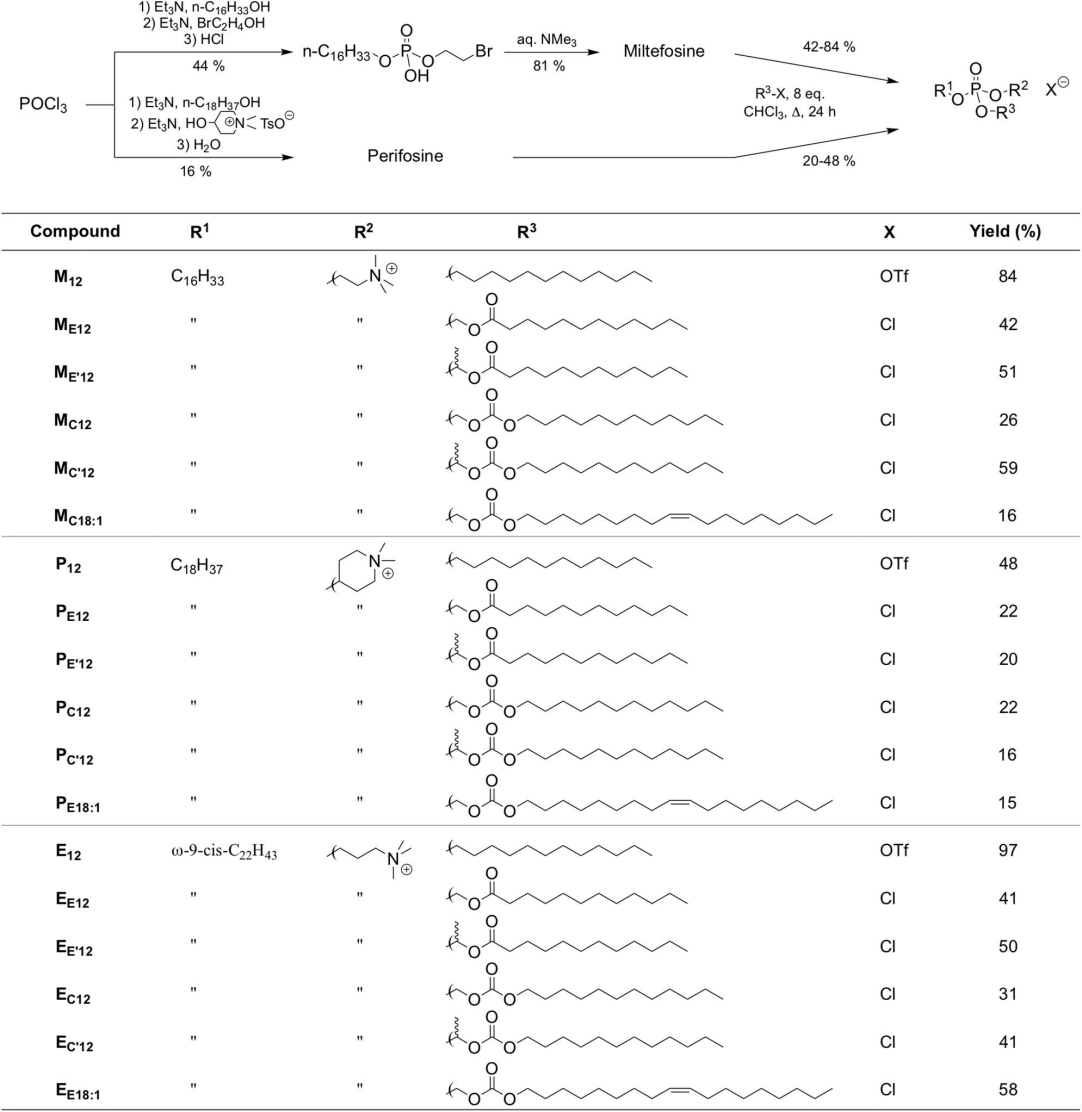
Synthetic route to APLs and pro-APLs. Data for erufosine prodrugs are reported from the literature (Gaillard et al., 2019).

However, the non-selective hydrolysis of an APL-derived phosphotriester into a phosphodiester would provide a mixture of the APL together with two other phosphodiester molecules. To favor the exclusive formation of the APL, it is thus of value to mask its phosphate group by introducing a (bio)labile phosphoester that will be removed preferentially, under a chemical or enzyme stimulus. Mixed phosphoacetals have been selected for this purpose, as they may degrade through the selective cleavage of the acetal bridge (Farquhar et al., 1983). Miltefosine and perifosine were thus converted into “pro-miltefosine” and “pro-perifosine” compounds incorporating various linkers for modulating their biodegradability which will determine their effectiveness as transfection reagents and the rate of *in situ* APL release (Figure 2). For comparison and SAR analysis, the previously described homolog compounds in the erufosine series (Gaillard et al., 2019) have been introduced in this study. The pro-miltefosine and pro-perifosine compounds were prepared in the ester (compounds noted **M**_****En****_ or **P**_****En****_) and carbonate (compounds noted **M**_****Cn****_ or **P**_****Cn****_) mixed phosphoacetal series. The acetal bridge received a methyl substituent (compounds noted **M**${\;}_{{\text{E}}^{\prime }\text{n}}$ and **M**${\;}_{{\text{C}}^{\prime }\text{n}}$) to modulate the hydrolysis rate of the compounds, as was recently demonstrated in phosphotriesters (Pierrat et al., 2013b). The compounds were straightforwardly synthesized by reaction of the parent APLs with a series of dodecyl-, dodecanoyl-, and oleyl-based electrophilic reagents (Figure 2) The reaction yields were generally lower than those obtained in the erufosine series (Gaillard et al., 2019). They ranged from 15 to 97%, depending both on the nucleophilicity of the phosphate group in the APL and on the reactivity of the electrophilic reagent. In some cases, the stability of the reaction product also was a obstacle to higher yields. As they contain two stereogenic centers, compounds with a methyl substitution on the acetal bridge (**M**${\;}_{{\text{E}}^{\prime }12}$, **M**${\;}_{{\text{C}}^{\prime }12}$, **P**${\;}_{{\text{E}}^{\prime }12}$, and **P**${\;}_{{\text{C}}^{\prime }12}$) were obtained as a mixture of four diastereomers. The separation of the two couples of enantiomers has been realized whenever possible, for analytic purpose, but the original mixture of isomers was used in the subsequent evaluations.

### Hydrolytic Stability of the Pro-APL Compounds

Aqueous formulation of the APL prodrugs produces nanosized lipid aggregates that most likely enter cells through the endocytosis pathway. To produce the expected intrinsic antineoplastic effect, APL-derived phosphotriesters need to be processed into the bioactive parent APLs. The hydrolysis of the prodrugs may be under the control of a chemical stimulus, e.g., a pH decrease as observed during the maturation of endosomes, or of degradation enzymes that massively enter the endosome compartment when continuing to lysosomes. To get some information on the transformation of pro-APLs into APLs, we monitored the hydrolysis of these compounds over time, at pH 7.4 and 4.5, conditions that mimick the extracellular milieu and the environment of the late endosome, respectively. This monitoring was realized using ^31^P-NMR spectroscopy, according to a previously reported method (Pierrat et al., 2013b). Whatever the compound investigated, only one single ^31^P resonance did appear during the course of the experiments, corresponding to that of the parent APL. This confirmed that hydrolysis of the pro-APLs selectively occurred at the acetal center. Consistently, phosphotriesters **M**_**12**_ and **P**_**12**_ lacking an acetal moiety revealed fully stable under both pH conditions, even after an extended incubation of 31 days at 25°C, as was previously observed for **E**_**12**_. All the other APL derivatives revealed sensitive to hydrolysis (Table 1). As a general trend, hydrolysis of the pro-APLs was quicker under neutral conditions (*i.e.*, pH 7.4) than under acidic ones (*i.e.*, pH 4.5). This revealed that the reactivity of mixed acetals of phosphoric and carboxylic or carbonic esters is completely different from that observed for dialkyl acetals that are more readily hydrolyzed under acidic conditions, and is consistent with results previously reported for other phosphoacetals (Pierrat et al., 2013a,b). Besides, substitution of one hydrogen atom with a methyl group on the acetal bridge accelerated the rate of hydrolysis of the prodrugs. This effect revealed more pronounced in the ester than in the carbonate series. With respect to the effect of the pendant arm tethered to the APLs through the acetal bridge on the rate of hydrolysis, the dodecyl and oleyl substituents did not show any significant difference. Finally, though these results allowed a comparison of the APL prodrugs with each other, it is important to consider that it could give an image of the stability of these compounds that is truncated. Indeed, in biological media, the enzyme-mediated hydrolysis of such compounds can proceed far quicker than “simple” chemical hydrolysis under pH control (Pierrat et al., 2012).

**Table 1:** Hydrolytic stability of the APL prodrugs.

**Compound**	**H**_****120****_ **(%)**		***t***_****1/2****_ **(h)**	
	**pH 7.4**	**pH 4.5**	**pH 7.4**	**pH 4.5**
**M**_**12**_^**[a]**^	0	0	–	–
**M**_**E12**_	20	3	–	–
**M**${\;}_{{\text{E}}^{\prime }12}$	89	91	18	17
**M**_****C12****_	11	3	–	–
**M**${\;}_{{\text{C}}^{\prime }12}$	14	13	–	–
**M**_****C18:**1**_	15	2	–	–
**P**_**12**_^**[a]**^	0	0	–	–
**P**_**E12**_	16	2	–	–
**P**${\;}_{{\text{E}}^{\prime }12}$	60	60	84	90
**P**_****C12****_	6	6	–	–
**E**_**12**_^**[a]**^	0	0	–	–
**E**_**E12**_	17	2	–	–
**E**${\;}_{{\text{E}}^{\prime }12}$	56	53	96	104
**E**_****C12****_	8	1	–	–
**E**${\;}_{{\text{C}}^{\prime }12}$	6	5	–	–
**E**_****C18:**1**_	10	0	–	–

*See Supplementary Material for detailed experimental conditions. H_120_ corresponds to the hydrolysis rate that was measured after a 120-h incubation period. Whenever possible, time needed for 50% hydrolysis (t_1/2_) was extrapolated from the experimental data. Data for erufosine prodrugs are reported from the literature (Gaillard et al., 2019). ^[a]^no significant hydrolysis was measured after a 31-d incubation period*.

### Gene Delivery Properties

The capacity of the pro-APLs to interact electrostatically with nucleic acids and form lipoplexes was checked by standard agarose gel electrophoresis. All the prodrugs tested led to full DNA complexation at a lipid/DNA phosphate ratio (N/P) > 1.3–3 (Supplementary Figure 1). The size (hydrodynamic diameter) and charge (zeta potential, ζ) of the lipoplexes prepared at an N/P ratio of 3 with one molar equivalent of dioleoyl-*sn*-glycero-3-phosphatidylethanolamine (DOPE), a lipid with fusogenic properties (Hui et al., 1996; Zuhorn et al., 2005), were investigated by DLS (Supplementary Table 1). The lipoplexes in the miltefosine series displayed a size in the range of 86–275 nm, which was similar to what was reported for DNA complexes with pro-erufosine compounds (Gaillard et al., 2019). In the perifosine series, larger complexes were formed (330–724 nm). This may be tentatively attributed to the expected higher main phase transition temperature of the perifosine derivatives as they display the longer saturated (stiffer) alkyl chain (Cevc, 1991). Introduction on the phosphate group of the APLs of an unsaturated chain resulted in significantly larger lipoplexes in the case of **M**_****C18:1****_ and **E**_****C18:1****_, (275 and 611 nm, resp.). Once again, results were different in the perifosine series and tethering an oleyl chain to the phosphate (**P**_****C18:1****_) did not translate into larger lipoplexes. With regard to the structure of the biolabile linker (ester or carbonate series, with or without substitution at the acetal bridge), no general trend could be observed. In the miltefosine series, lipoplexes prepared from acetal esters (**M**_**E12**_, **M**${\;}_{{\text{E}}^{\prime }12}$) and acetal carbonates (**M**_****C12****_, **M**${\;}_{{\text{C}}^{\prime }12}$) did not differ in size, but the introduction of a methyl substituent at the acetal center led to lipoplexes with half the size of those prepared from the unsubstituted compounds. In the perifosine series, acetal substitution had the opposite effect. Considering the pro-erufosine compounds, smaller lipoplexes were obtained with the biolabile derivatives **E**_**E12**_, **E**${\;}_{{\text{E}}^{\prime }12}$, **E**_****C12****_, and **E**${\;}_{C^{\prime }12}$ as compared to phosphotriester **E**_**12**_ (92–140 *vs*. 194 nm), and substitution at the acetal bridge had no significant effect on the size of the lipoplexes (Gaillard et al., 2019). As could be expected considering electrophoretic behavior, all the pro-APL/DNA complexes displayed a net positive charge at N/P 3, with ζ values spreading from +16 to +54 mV.

**Figure 3:**
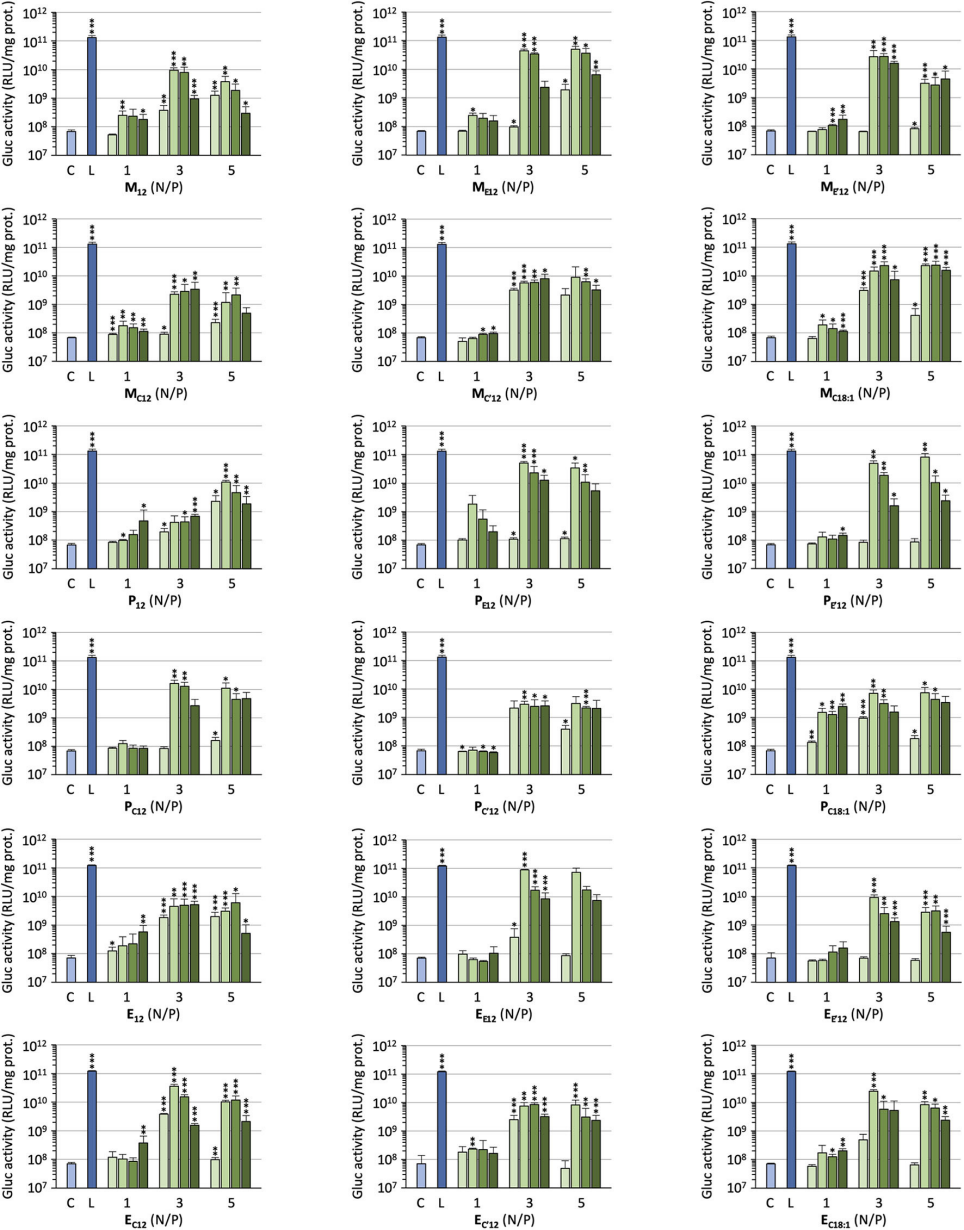
Gene transfer efficiency of pro-APLs in A549 cells. Lipoplexes were prepared by introducing DOPE in the formulations (0, 1, 2, and 3 molar equivalents, from light to dark green, resp.) and varying the N/P ratio from 1 to 5. Control (C) refers to basal bioluminescence measured in untreated cells. Lipofectamine® 2000 (L) was used as a positive control. Data for pro-erufosine compounds are reported from our previous work (Gaillard et al., 2019). Statistical significance *vs*. control. ^*^**p < 0.001, ^*^*p < 0.01, ^*^p < 0.05.

The efficacy of the pro-APLs to mediate intracellular delivery of a plasmid DNA was examined in A549 human lung epithelial carcinoma cells using the pCMV-Gluc reporter gene encoding the luciferase of *Gaussia princeps*. This luciferase is secreted by cells and transgene expression can be conveniently assessed by standard bioluminescence measurements on aliquots of the cell culture supernatant, without need for prior cell lysis. Lipoplexes were formulated in glucose 5% at N/P ratios of 1, 3, and 5, with increasing amounts of DOPE as a helper lipid. They were deposited onto cells in the presence of 10% serum and luciferase activity was measured after 24 h (Figure 3). A number of general trends emerged. Firstly, lipoplexes prepared at the lower charge ratio (N/P = 1) failed to mediate any significant transgene expression. This was consistent with the results obtained in gel electrophoresis revealing that full complexation of DNA required an excess of cationic lipid (N/P > 1.3–3). Accordingly, increasing the N/P ratio to 3 or 5 allowed for obtaining high transfection rates whatever the pro-APL considered. Secondly, whatever the N/P value, DOPE-free formulations revealed only poorly efficient for mediating transgene expression. The best results were thus generally obtained with 1–2 molar equivalents of the helper lipid whereas higher proportion of DOPE appeared deleterious as evidenced by degraded transfection rates. Thirdly, with regard to the structure of the biolabile linker, pro-APLs in the ester series invariably outperformed those in the carbonate series, and allowed transfection rates that were mostly 3–10 times higher. The only differences in the size of the lipoplexes (*vide supra*), a larger size favoring particle sedimentation and cell uptake (Rejman et al., 2004), cannot explain this effect. These results thus suggest that it is the rate at which the pro-APLs are intracellularly hydrolyzed that has a major impact on the transfection efficiency. On the other hand, the introduction of a methyl substituent on the acetal bridge had mixed effects, depending on the compounds. Most of the time, this modification led to some decrease in the transfection rate. One exception however was observed in the miltefosine series, **M**${\;}_{{\text{C}}^{\prime }12}$ allowing a higher transfection rate than **M**_****C12****_ by a *ca*. 2-fold factor. The influence of the nature of the pendant hydrophobic arm was also examined. In the miltefosine series, **M**_****C18:1****_ showed significantly enhanced transfection properties as compared to **M**_****C12****_. In the perifosine series, the opposite effect was observed and oleyl derivatives performed a little less than dodecyl derivatives. The same trend has been observed in the erufosine series (Gaillard et al., 2019). Finally, pro-APL compounds lacking a biolabile linker, *i.e.*, **M**_**12**_, **P**_**12**_, and **E**_**12**_, were not systematically outperformed by their more labile analogs. This revealed that enhanced biodegradability of the gene carriers did not necessarily translate into higher transgene expression. More important, likely, was where and when the degradation of the vector did occur.

**Figure 4:**
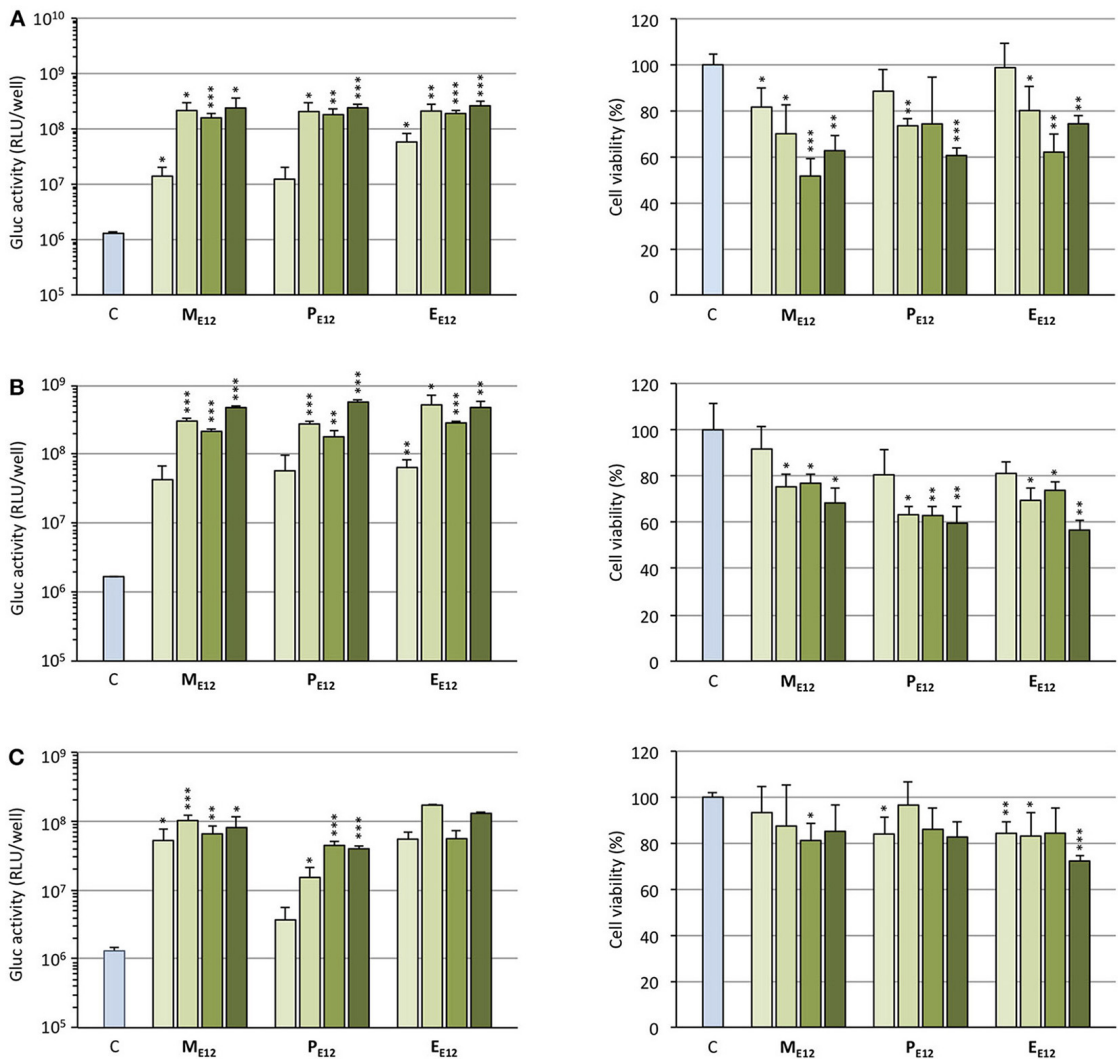
**(A)** Rate of transfection (left) mediated by pro-APLs in A549 cells with increasing dose of pCMV-Gluc (0.1, 0.2, 0.3, and 0.4 μg/well, from light to dark green, resp.) and cytotoxicity of the transfection particles (right). **(B)** Rate of transfection (left) mediated by pro-APLs in H292 cells, and cytotoxicity of the transfection particles (right). **(C)** Rate of transfection (left) mediated by pro-APLs in 16HBE cells, and cytotoxicity of the transfection particles (right). Transfection experiments were carried out in the presence of 10% serum. Control refers to basal bioluminescence measured in untreated cells. Cell viability was assessed by mitochondrial activity measurements using the MTT assay. Basal mitochondrial activity measured in untreated cells is set at 100%. Data for **E**_**E12**_ are reported from our previous work (Gaillard et al., 2019). Statistical significance *vs*. control. ***p < 0.001, **p < 0.01, *p < 0.05.

Selecting **M**_**E12**_ and **P**_**E12**_ that performed the best in the previous experiments, a dose-response study was conducted. A549 epithelial cells were exposed to increasing amounts of pCMV-Gluc (from 0.1 to 0.4 μg/well) formulated into lipoplexes (pro-APL/DOPE 1/1) at the N/P ratio of 3. As a general trend, transfection rate reached a plateau at the intermediate pDNA dose of 0.2 μg/well (Figure 4A, left). Increasing the dose above this value did not significantly improve the transfection rate, while decreasing cell viability (Figure 4A, right). The same behavior was reported with **E**_**12**_ (Gaillard et al., 2019). Similar transfection and cytotoxicity profiles were obtained in two other pulmonary epithelial cell lines (human bronchial epithelial cells 16HBE and human lung mucoepidermoid carcinoma cells H292) (Figures 4B,C).

### Serum Compatibility

**Figure 5:**
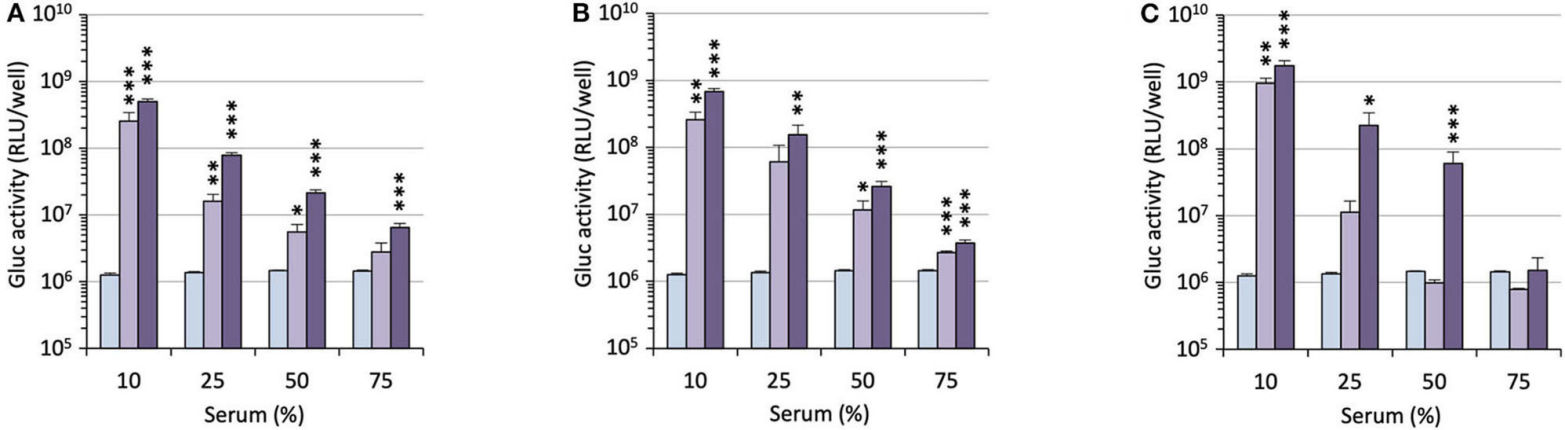
Transfection efficiency of pro-APLs **M**_**E12**_ **(A)**, **P**_**E12**_ **(B)**, and **E**_**E12**_ **(C)** as a function of serum content. Pro-APL/DNA complexes were prepared at N/P 3, with 1 molar eq. of DOPE. A549 cells (6,000 cells/well) were seeded into 96-well plates in 100 μL of culture medium containing 10% serum. Twenty-four hours later, cell supernatant was replaced with culture medium complemented with increasing amounts of serum (from 10 to 75%) just before adding the lipoplexes (0.2 and 0.4 μg of DNA per well; light and deep purple bars, resp.). Control (light blue bar) refers to basal bioluminescence measured in untreated cells. Data for **E**_**E12**_ are reported from our previous work (Gaillard et al., 2019). Statistical significance *vs*. control. ***p < 0.001, **p < 0.01, *p < 0.05.

For potential clinical applications, the interaction of positively charged lipoplexes and negatively charged proteins in the bulk medium cannot be ignored (Pouton and Seymour, 2001). Thus, a limitation of gene delivery mediated by cationic non-viral carriers is a drastic decrease of transfection efficiency in the presence of serum proteins forming the so-called “biocorona” around the transfection particles. This protein corona drastically changes the identity of the particles, may provoke their destabilization or inhibit their cell uptake and endosome escape. To investigate the effect of serum on the gene delivery properties of the APL prodrugs, cells were treated with lipoplexes in the presence of an increasing concentration of serum (Figure 5). Two DNA doses were considered. As might be expected, transgene expression was decreased with increasing serum concentration though at various rates, depending on the pro-APL compound involved in the formulation. At the lower DNA dose, *i.e.*, 0.2 μg/well, **M**_**E12**_ and **P**_**E12**_ mediated significant transgene expression up to 50% serum, which is about the concentration in blood. At the higher DNA dose, 0.4 μg/well, transfection activity extended up to 75% serum. Besides, though pro-APL **E**_**E12**_ revealed less efficient at the low DNA dose, it provided the best transfection results at the high DNA dose in 50% serum. These results thus demonstrate that the herein described APL prodrugs are serum compatible nucleic acid carriers that can mediate efficient gene delivery at high serum content.

### Self-Assembly Properties

The pro-APL molecules have been specifically engineered so they can be metabolized *in situ* under a biological stimulus to revert to the parent APL with antiproliferative activity. As membrane-active compounds, APLs can disrupt cell membranes or alter cholesterol homeostasis, and thus interfere with various membrane signaling pathways involved in carcinogenesis. It has been shown that the lytic concentration for various APLs in the absence of serum reflects their critical micellar concentration (CMC) (Fleer et al., 1992; Kötting et al., 1992). We thus aimed to determine the self-assembly properties of the APL prodrugs. For that, we applied a pyrene-based fluorescence technique that is commonly used to analyze surfactant micellization (Goddard et al., 1985; Piñeiro et al., 2015). Pyrene is a probe which fluorescence is sensitive to the polarity of the solubilizing medium. Hence, fluorescence emission can be used to detect self-assembly or aggregation of amphiphilic compounds. Due to the introduction of an “additional” hydrophobic element within the original APL structure, pro-APL compounds were expected to display a lower hydrophilic-lipophilic balance (HLB) than the parent APLs and assemble into lamellar structures in aqueous media. Therefore, the term critical aggregation concentration (CAC) is suggested instead of CMC (Parlato et al., 2011). Fluorescence measurements were carried out in ultrapure water and data obtained for the various APLs and their prodrugs are shown in Table 2. The CMC measured for miltefosine, perifosine, and erufosine were 11.2, 4.4, and 1.6 μM, respectively. The value determined for miltefosine was consistent with that previously obtained by the Du Noüy ring method (12 μM) (Yaseen et al., 2005) whereas the Langmuir trough method yielded a lower one (2.5–3.0 μM) (Rakotomanga et al., 2004). For perifosine, the CMC value was similar to that determined by the Langmuir trough method (2.5 μM) (Mravljak et al., 2005).

**Table 2:** CMC of APLs and CAC of pro-APLs as measured using a fluorescent probe technique.

**Compound**	**CAC (μM)**	**Compound**	**CAC (μM)**	**Compound**	**CAC (μM)**
Miltefosine	11.2 ± 2.3	Perifosine	4.4 ± 1.7	Erufosine	1.6 ± 0.5
**M**_**12**_	1.1 ± 0.6	**P**_**12**_	1.2 ± 0.4	**E**_**12**_	0.7 ± 0.3
**M**_**E12**_	4.3 ± 2.5	**P**_**E12**_	2.9 ± 1.0	**E**_**E12**_	0.6 ± 0.2
**M**${\;}_{{\text{E}}^{\prime }12}$	1.3 ± 0.7	**P**${\;}_{{\text{E}}^{\prime }12}$	2.3 ± 1.4	**E**${\;}_{{\text{E}}^{\prime }12}$	1.1 ± 0.7
**M**_****C12****_	4.1 ± 2.5	**P**_****C12****_	4.5 ± 1.6	**E**_****C12****_	1.2 ± 0.2
**M**${\;}_{{\text{C}}^{\prime }12}$	2.0 ± 0.7	**P**${\;}_{{\text{C}}^{\prime }12}$	3.3 ± 0.7	**E**${\;}_{{\text{C}}^{\prime }12}$	0.9 ± 0.2
**M**_****C18:**1**_	0.9 ± 0.4	**P**_****C18:**1**_	2.3 ± 1.0	**E**_****C18:**1**_	0.8 ± 0.4

*Data for erufosine and erufosine prodrugs are reported from the literature (Gaillard et al., 2019)*.

As expected, the transformation of APLs into pro-APLs, by neutralizing the phosphate negative charge and tethering of an additional hydrophobic substituent, translated into a shift of the HLB which resulted in a decrease in the CMC/CAC values. Larger effects were observed in the pro-miltefosine series as compared to the pro-perifosine series. In the pro-erufosine series, lower variations were reported (Gaillard et al., 2019). All this was consistent with the increasing length of the “main” alkyl chain in the APL molecules (miltefosine: C_16_; perifosine: C_18_; erufosine: C_22_). On the other hand, no particular trend could be identified with respect to some relationship between the nature of the labile spacer (*i.e.*, phosphoester *vs*. phosphoacetal, ester *vs*. carbonate, and non-substituted *vs*. substituted acetal) and CAC of the compounds. However, the variations measured were small and accuracy of the pyrene-based technique was limited. Thus, tentatively getting a deeper understanding of the structure-property relationships would require a more precise determination of the CAC values.

### Hemolytic Activity

**Figure 6:**
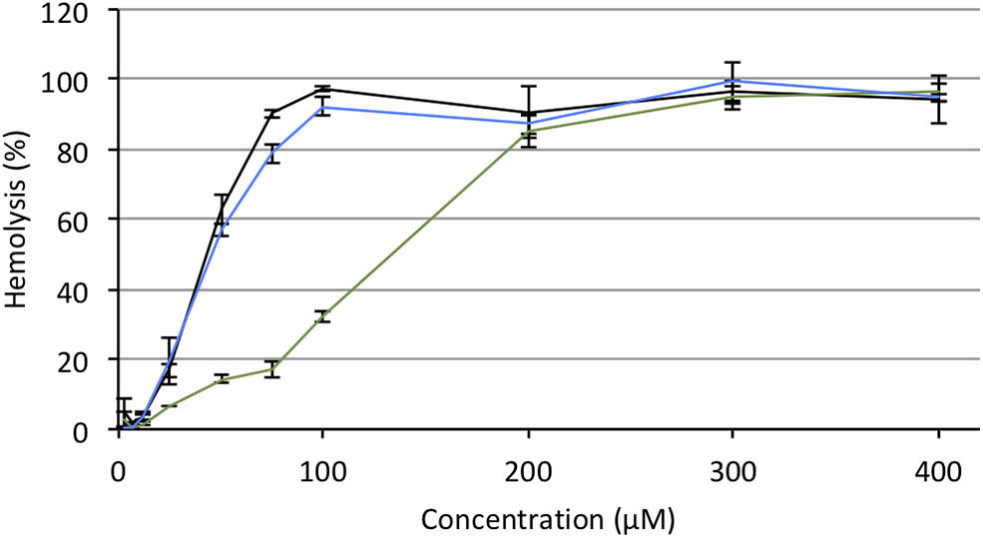
Hemolytic effect of miltefosine (black), perifosine (blue), and erufosine (green) on sheep RBC incubated at 37°C for 1 h, as a function of the APL concentration.

Hemolytic effect is systematically reported for most of the antitumor APLs that have been described so far and makes these compounds incompatible with intravenous administration (Kötting et al., 1992). APLs alter the thermotropic behavior of lipid membranes, increasing their fluidity. In red blood cells (RBC), such an alteration of the plasma membrane induces drastic morphological changes and provokes the release of hemoglobin (Petit et al., 2019). In serum-free conditions, there is a good fit between the hemolytic concentration of APLs and their CMC (Fleer et al., 1992; Kötting et al., 1992). Consequently, decreasing the CMC of APLs by turning them reversibly into derivatives with enhanced hydrophobicity should result in decreasing the hemolytic effect, thus increasing the biocompatibility of the compounds. To evaluate this hypothesis, the hemolytic activity of the APLs and pro-APLs was measured. Sheep RBC were incubated for 1 h with escalating doses of aqueous dispersion of the compounds at 37°C. At the end of the incubation period, hemoglobin leakage was determined spectrophotometrically. As expected, APLs provoked massive hemolysis in a dose-dependent manner. The concentration provoking 50% hemolysis (HC_50_, calculated by using the best-fit regression curve method) was 43 and 46 μM for miltefosine and perifosine, respectively (Figure 6). The HC_50_ value found for miltefosine was consistent with those reported in the literature (41 μM Petit et al., 2019, and 34–51 μM Alonso and Alonso, 2016, depending on the hematocrit). Though other authors have reported *ca*. 30% hemolysis after a 24-h exposure to 5.4 μM perifosine (Egler and Lang, 2017), a direct correlation with our results was difficult, due to significant differences in the experimental conditions.

**Figure 7:**
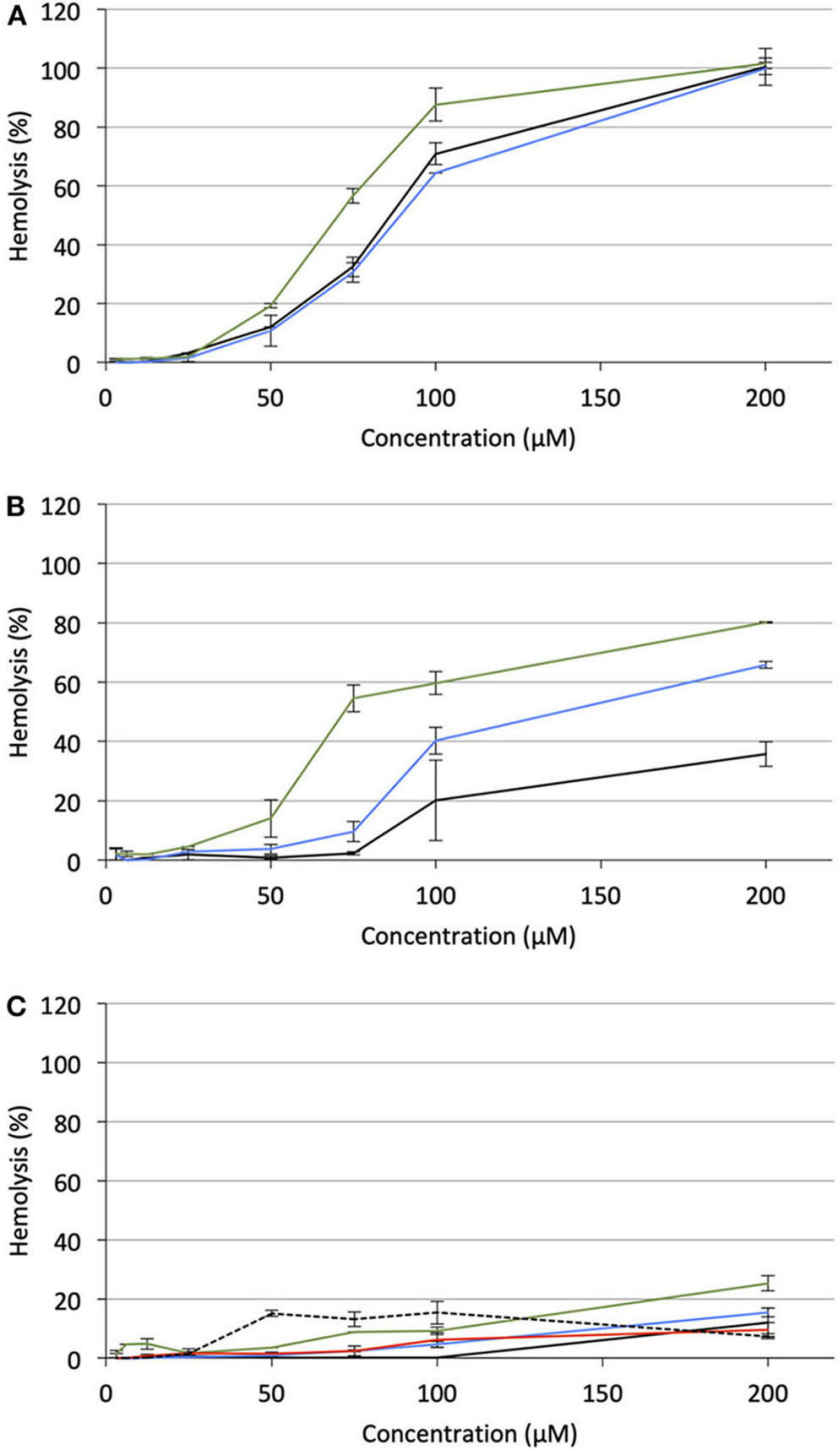
Hemolytic effect of pro-APL compounds on sheep RBC incubated at 37°C for 24 h, as a function of the prodrug concentration. **(A)** Pro-miltefosine series (**M**_**12**_: black; **M**_**E12**_: blue; **M**${\;}_{{\text{E}}^{\prime }12}$: green). **(B)** Pro-perifosine series (**P**_**12**_: black; **P**_**E12**_: blue; **P**${\;}_{{\text{E}}^{\prime }12}$: green). **(C)** Pro-erufosine series (**E**_**12**_: black; **E**_**E12**_: blue; **E**${\;}_{{\text{E}}^{\prime }12}$: green; **E**_****C12****_: red; **E**${\;}_{{\text{C}}^{\prime }12}$: dashed).

Under the same incubation conditions as those used for APL analysis, hemolytic effect of the APL prodrugs was substantially lower. Table 3 displays the hemolytic activity of the compounds at 200 μM (HA_200_) after a 1-h incubation. In most cases, <10–20% hemolysis was measured. Thus, in order to better characterize the hemolytic effect of the pro-APLs, the incubation period has been extended and release of hemoglobin was measured after 24 h. Even under these more challenging conditions, the APL prodrugs revealed poorly harmful to RBC and significant hemolysis (> 20%) was measured only at a concentration above *ca*. 50–100 μM (Figure 7). The corresponding HA_200_ values are presented in Table 3. The direct correlation between the structure of the biolabile linker in the pro-APL molecules and their hemolytic effect (SAR analysis) is especially difficult due to the unique biodegradability profile of each compound, so no general trend was identified. Nevertheless, our data show that the pro-APLs display drastically reduced hemolytic effect as compared to the parent APLs. Especially, it is worth to note that hemolysis provoked by miltefosine and perifosine prodrugs after 24 h was much lower in most cases than that induced by erufosine after 1 h, and erufosine prodrugs were practically devoid of any hemolytic activity (HA_200_ <25%, after 24 h). As erufosine is the only APL currently considered for intravenous administration (Bagley et al., 2011), it can be concluded that all the prodrugs of miltefosine and perifosine described herein could be safely qualified for this administration route.

**Table 3:** Hemolytic activity of the APLs and pro-APLs (200 μM) upon incubation with sheep RBC for 24 h at 37°C.

**Compound**	**HA**_****200****_ **(%)**	
	**1 h**	**24 h**
Miltefosine	90.8 ± 1.5	n.d.
**M**_**12**_	13.1 ± 1.0	63.9 ± 8.1
**M**_**E12**_	18.0 ± 1.2	103.2 ± 3.6
**M**${\;}_{{\text{E}}^{\prime }12}$	n.d.	101.7 ± 1.8
**M**_****C12****_	6.0 ± 1.4	59.7 ± 5.4
**M**${\;}_{{\text{C}}^{\prime }12}$	21.5 ± 2.3	70.8 ± 4.4
**M**_****C18:**1**_	1.3 ± 1.2	12.5 ± 2.8
Perifosine	85.1 ± 4.0	n.d.
**P**_**12**_	8.1 ± 0.9	35.7 ± 4.1
**P**_**E12**_	7.1 ± 0.9	20.6 ± 5.9
**P**${\;}_{{\text{E}}^{\prime }12}$	n.d.	80.1 ± 0.2
**P**_****C12****_	< 1	60.7 ± 2.2
**P**${\;}_{{\text{C}}^{\prime }12}$	< 1	24.4 ± 7.6
**P**_****C18:**1**_	n.d.	n.d.
Erufosine	84.9 ± 4.5	n.d.
**E**_**12**_	1.9 ± 0.7	12.0 ± 1.9
**E**_**E12**_	< 1	18.6 ± 1.5
**E**${\;}_{{\text{E}}^{\prime }12}$	n.d.	25.3 ± 2.6
**E**_****C12****_	< 1	9.6 ± 2.3
**E**${\;}_{{\text{C}}^{\prime }12}$	< 1	7.3 ± 0.8
**E**_****C18:**1**_	< 1	20.9 ± 2.5

*Data for erufosine and erufosine prodrugs are reported from the literature (Gaillard et al., 2019). n.d.: not determined*.

### Cytotoxicity

The cytotoxicity of APLs does not just have to do with the ability of these compounds to destabilize lipid membranes and lyse cells. There is broad agreement that, at concentrations that are pharmacologically relevant, APLs modify the cell membrane fluidity and interfere with the metabolism of phospholipids and homeostasis of cholesterol, hence provoking perturbations in various signal transduction pathways, some of which are essential for cell functioning, survival and proliferation (Ríos-Marco et al., 2017). In order to evaluate the antiproliferative potency of the pro-APL compounds, a dose-response study was conducted in the three human pulmonary epithelial cell lines considered in this study: A549 (alveolar carcinoma epithelial cells), H292 (mucoepidermoid carcinoma cells), and 16HBE (bronchial epithelial cells). The cells were exposed for a 24-h period to increasing concentration of the pro-APLs, ranging from 500 nM to 1 mM, and cell viability was assessed using the MTT colorimetric assay (Mosmann, 1983). For the three cell lines, viability decreased in a dose-dependent manner in response to APLs and pro-APLs (Supplementary Figures 2–4). Using the best-fit regression curve method, the IC_50_ value (concentration that provokes 50% inhibition of the cell growth) was calculated for each compound (Table 4). The three cell lines responded to the compounds with varying sensitivities. The IC_50_ values extended from 39 to 326 μM for A549 cells, from 46 to 295 μM for H292 cells, and from 19 to 257 μM for non-cancerous 16HBE cells. The cell toxicity of the APL prodrugs did not correlate with their hydrolytic susceptibility as determined above by the ^31^P-NMR measurements in an “enzyme-free” model. This is attributed to enzyme-mediated hydrolysis of the pro-APL compounds in the complemented culture medium or in the intracellular compartment, competing with pure pH-controlled chemical hydrolysis. According to its structure, a pro-APL may or not be a substrate for specific lipases and, thus, may or not be hydrolyzed into the parent cytotoxic compound. Also, no direct correlation could be found between aggregation properties (CAC) and IC_50_ values, supporting that cytotoxicity of APLs and pro-APLs did not solely express through the membrane disrupting properties of the compounds or of their hydrolysis product. Nevertheless, a marked cytotoxicity was occasioned by all the pro-APL compounds that roughly compared to that of the parent APLs, revealing that the prodrugs were productively metabolized into APLs, although not at the same rate. In the case of miltefosine and perifosine, some of the prodrugs were more potent than the parent compounds and some were less, results varying from one cell line to another. With respect to the erufosine prodrugs, although they all revealed highly cytotoxic, none of them did perform better than erufosine itself, as was previously reported (Gaillard et al., 2019).

**Table 4:** Antitumor activity of APLs and APL prodrugs.

**Compound**	**IC**_****50****_ **(μM)**		
	**A549**	**H292**	**16HBE**
Miltefosine	77 ± 15	70 ± 9	73 ± 8
**M**_**12**_	46 ± 11	56 ± 7	107 ± 14
**M**_**E12**_	39 ± 7	52 ± 28	69 ± 26
**M**${\;}_{{\text{E}}^{\prime }12}$	139 ± 32	59 ± 10	49 ± 6
**M**_****C12****_	151 ± 42	81 ± 29	62 ± 15
**M**${\;}_{{\text{C}}^{\prime }12}$	48 ± 15	146 ± 37	66 ± 27
**M**_****C18:**1**_	92 ± 22	135 ± 41	50 ± 12
Perifosine	127 ± 39	77 ± 3	116 ± 33
**P**_**12**_	56 ± 7	63 ± 5	72 ± 7
**P**_**E12**_	139 ± 4	168 ± 7	231 ± 70
**P**${\;}_{{\text{E}}^{\prime }12}$	148 ± 38	116 ± 25	60 ± 5
**P**_****C12****_	89 ± 24	73 ± 17	82 ± 33
**P**${\;}_{{\text{C}}^{\prime }12}$	189 ± 52	295 ± 33	145 ± 34
**P**_****C18:**1**_	273 ± 69	186 ± 42	207 ± 49
Erufosine	57 ± 8	46 ± 8	19 ± 3
**E**_**12**_	81 ± 7	124 ± 11	225 ± 31
**E**_**E12**_	151 ± 42	125 ± 26	144 ± 66
**E**${\;}_{{\text{E}}^{\text{′}}12}$	192 ± 44	73 ± 15	63 ± 7
**E**_****C12****_	161 ± 21	167 ± 56	252 ± 112
**E**${\;}_{{\text{C}}^{\prime }12}$	179 ± 63	161 ± 25	257 ± 81
**E**_****C18:**1**_	326 ± 36	154 ± 40	238 ± 58

*The IC_50_ values were determined in various cell lines exposed to the compounds for 24 h at 37°C, using the MTT assay. Data for erufosine and erufosine prodrugs are reported from the literature (Gaillard et al., 2019)*.

### Combined Antitumor Activity of TRAIL and APL Prodrugs

In order to investigate the potential of APL prodrugs in combined antitumor therapy, we performed gene delivery experiments with pUNO1-hTRAIL. This plasmid DNA encodes the tumor necrosis factor-related apoptosis-inducing ligand (TRAIL), a member of the tumor necrosis factor (TNF) superfamily (Shi et al., 2018). TRAIL suppresses tumor growth by a direct and specific mechanism without affecting normal tissues (Walczak et al., 1999; Nair et al., 2015). Interestingly, perifosine and edelfosine, another compound related to the APL family, have been reported to sensitize cells to TRAIL-induced apotosis (Gajate and Mollinedo, 2007; Lim et al., 2016). This, however, has not been demonstrated for other APLs so far. Expression of the TRAIL receptors in the three cell lines considered herein has been assessed elsewhere (Azijli et al., 2014; Gaillard et al., 2019). Both TRAIL death receptors, TRAIL-R1 and TRAIL-R2, were found expressed in the cell lines, in comparable amounts, except for TRAIL-R1 which expression in A549 cells is higher. Among the TRAIL decoy receptors that may impair TRAIL activity, *i.e.*, TRAIL-R3 and TRAIL-R4, TRAIL-R3 was found in H292 and 16HBE cells, but not in A549 cells, while TRAIL-R4 was missing in the three cell lines. To sum up, the selected cells may stand as suitable models for gene delivery experiments with a pDNA encoding TRAIL for apoptosis induction.

**Figure 8:**
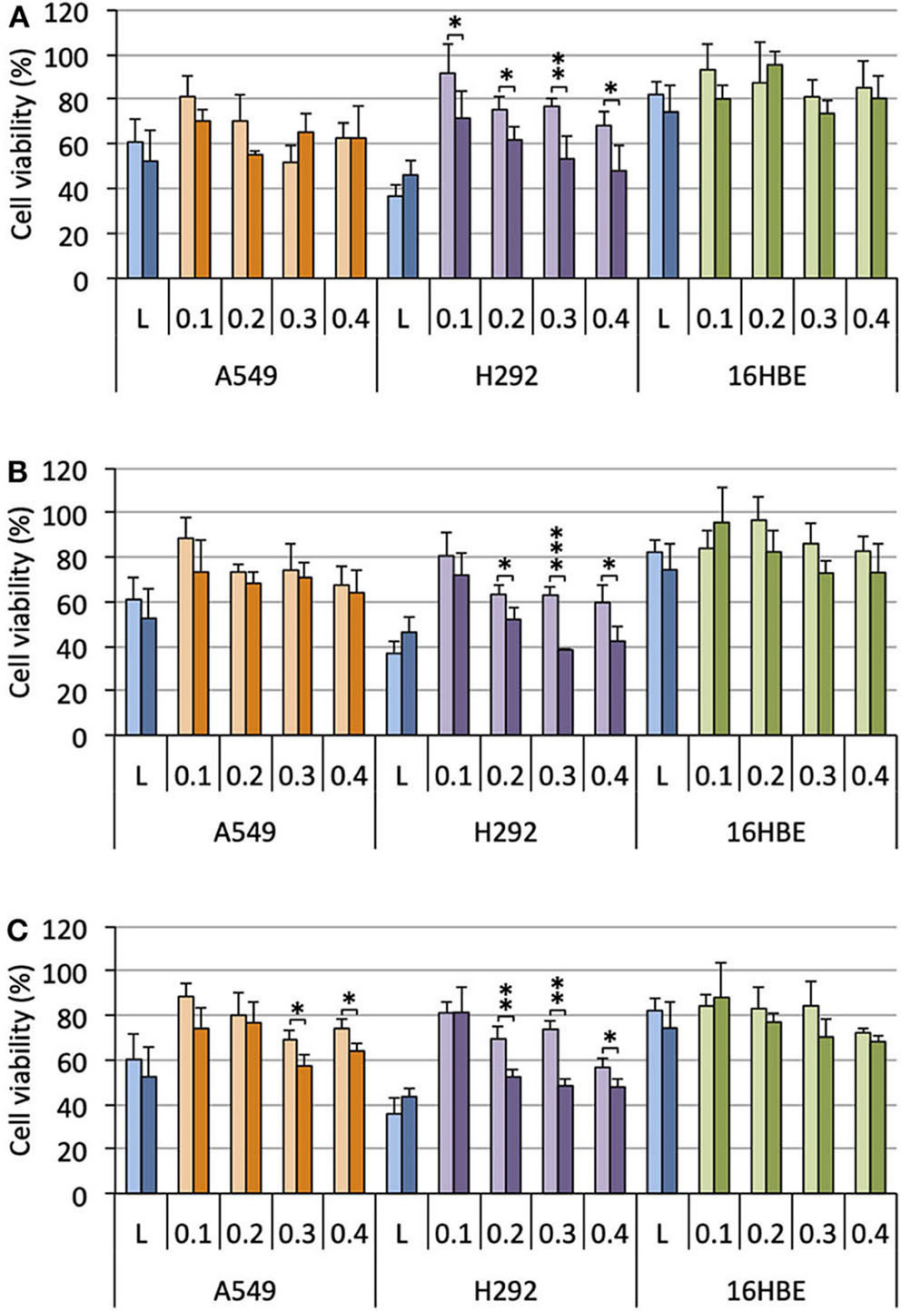
Antiproliferative effect of lipoplexes prepared from **M**_**E12**_ **(A)**, **P**_**E12**_ **(B)**, **E**_**E12**_ **(C)**, and the two DNA plasmids pCMV-Gluc (light bars) and pUNO1-hTRAIL (dark bars), on the three cell lines. The lipid/pDNA complexes were formulated at N/P 3 with 1 molar eq. of DOPE, and an increasing pDNA dose (from 0.1 to 0.4 μg/well). Lipofectamine®-based lipoplexes (0.1 μg plasmid/well) were prepared according to the optimized experimental conditions determined following the supplier's instructions. Cell viability was measured by the MTT assay. Basal mitochondrial activity measured in untreated cells is set at 100%.

Cells were treated with lipoplexes prepared from pro-APLs **M**_**E12**_, **P**_**E12**_, and **E**_**E12**_, with increasing dose of pDNA (0.1–0.4 μg per well). The gold standard gene delivery reagent Lipofectamine® 2000 was assayed in parallel for comparison purpose. In order to distinguish the intrinsic cytotoxic effect of the *in situ* generated APLs from that resulting from TRAIL expression, pCMV-Gluc was assayed in parallel to pUNO1-hTRAIL. Using pCMV-Gluc, the observed cytotoxicity was assumed to result only from the APL antiproliferative activity, whereas results obtained with pUNO1-hTRAIL are a combination of TRAIL activity and APL antiproliferative effect. Besides, monitoring of luciferase expression allowed assessing the transfection efficiency with the various carriers in the three cell lines (Figure 4), suggesting that replacement of the plasmid DNA coding for luciferase with pUNO1-hTRAIL should indeed result in significant TRAIL expression. As a general trend, a slow decrease in cell viability was observed with pUNO1-hTRAIL, as compared to pCMV-Gluc (Figure 8). Though the temptation is to take it as an indicator that functional TRAIL was produced, the difference found was not always statistically significant. The activity profile of the three pro-APLs varied according to the cell line. In A549 cells that are poorly sensitive to TRAIL (Azijli et al., 2014), no significant cytotoxic effect of the transgene expression product was evidenced, except for the pro-erufosine-based lipoplexes. Indeed, at the high pDNA doses (0.3 and 0.4 μg/well), **E**_**E12**_ led to 15-17% of TRAIL-induced cell death. The H292 cell line was much more sensitive to TRAIL, right from the lower dose of transgene and regardless of the pro-APL. This was consistent with previous reports in the literature describing the high sensitivity of H292 cells to TRAIL (Azijli et al., 2014). Depending on the pro-APL used to deliver the plasmid, cell death specifically induced by TRAIL could reach *ca*. 45%, and the higher score was attained with the pro-perifosine compound, **P**_**E12**_, in this cell line. In any case, the lipoplexes incorporating the plasmid encoding TRAIL led to a decrease in viability mostly below 50-55%, whatever the pro-APL used. On the other hand, no such effect was observed with Lipofectamine®, though only the lower DNA dose (0.1 μg/well) was assayed in this case, due to the high intrinsic cytotoxicity of the reagent that likely overwrite TRAIL activity. Finally, given their non-tumor origin, 16HBE cells were not affected by TRAIL and no specific cell toxicity was observed upon treatment with pUNO1-hTRAIL. Summing up, the combination of the APL prodrugs and pUNO1-hTRAIL revealed harmful to A549 and H292 carcinoma cells (cell death > 40–50%) while preserving normal 16HBE cells (cell death <25%), and the two pro-miltefosine and pro-perifosine compounds, **M**_**E12**_ and **P**_**E12**_, did perform as well as or better than the previously described erufosine prodrug **E**_**E12**_.

## Conclusion

In this study, biolabile miltefosine- and perifosine-based cationic lipids were engineered and their properties as gene carriers and antineoplastic prodrugs were investigated in normal and carcinoma cells. Transfection efficiency was optimized varying the charge ratio of the lipoplexes and their DOPE content. Significant serum resistance of the lipoplexes was demonstrated, the pro-APLs maintaining a high transfection rate even at a serum content above 50%. Contrary to the parent compounds miltefosine and perifosine, the pro-APLs did not show any hemolytic activity which is a dose-limiting side effect of the APLs in antitumor therapy. All the pro-APLs investigated herein displayed cytotoxic effects in the same concentration range as the parent APLs, revealing that they were properly metabolized into APLs. Finally, gene delivery experiments with a DNA plasmid encoding TRAIL that can trigger apoptosis in a wide variety of cancer cells, but not in normal cells, provided a proof of concept for a new promising strategy for cancer therapy combining gene therapy and APL-based chemotherapy.

## Data Availability Statement

All datasets generated for this study are included in the article/Supplementary Material.

## Author Contributions

LL designed the research and wrote the manuscript with editorial input from J-SR and FP. LL and BG performed the chemical synthesis experiments. BG performed the biological experiments under J-SR, FP, and LL supervision. All authors contributed to the article and approved the submitted version.

## Conflict of Interest

The authors declare that the research was conducted in the absence of any commercial or financial relationships that could be construed as a potential conflict of interest.
