# Supplementary material for: Dual Gene Delivery Reagents From Antiproliferative Alkylphospholipids for Combined Antitumor Therapy
Source: Front Chem. 2020 Oct 2;8:581260. doi: 10.3389/fchem.2020.581260 (PMC7566913; doi:10.3389/fchem.2020.581260)
Supplement: Supplementary file 1 [file Data_Sheet_1.pdf]

Supplementary Material

CONTENTS

General	2
Supplementary Figures	5
Supplementary Table	9
^{1}H-, ^{13}C-, and ^{31}P-NMR spectra	10
References	31

General

Materials

Unless otherwise stated, all chemical reagents were purchased from Alfa Aesar (Bischeim, France) and used without purification. When required, solvents were dried just before use as described elsewhere (Lebeau et al, 1992). Thin layer chromatography (TLC) was performed on precoated plates (0.25 mm Silica Gel 60, F_{254}, Merck, Darmstadt, Germany). Products were purified by flash chromatography over silica gel (Silica Gel 60, 40-63 µm, Merck, Darmstadt, Germany). NMR spectra were recorded on Bruker 400 MHz Avance III instrument. ^{1}H-, ^{13}C-, and ^{31}P-NMR chemical shifts δ are reported in ppm relative to their standard reference (^{1}H: $CHCl_3$ at 7.27 ppm, CD_2HOD at 3.31 ppm; ^{13}C: $CDCl_3$ at 77.0 ppm, CD_3OD at 49.0 ppm; ^{31}P: $(MeO)_2P(O)Me$ at 38.78 ppm). IR spectra were recorded on a FT-IR Nicolet 380 spectrometer in the ATR mode and absorption values ν are in wave numbers (cm-1). Mass Spectra (MS) were recorded on an Agilent Technologies 6520 Accurate Mass QToF instrument, using electrospray ionization (ESI) mode. Mass data are reported in mass units (m/z). 1,2-Dioleoyl-sn-glycero-3-phosphoethanolamine (DOPE) was from Avanti Polar Lipids (Alabaster, USA). Lipofectamine 2000® was obtained from Invitrogen (Cergy Pontoise, France). Plasmid pCMV-Gluc (5.7 kbp) and coelenterazine substrate for monitoring Gaussia luciferase activity were from Nanolight Technology (Pinetop, Az, USA). Plasmid pUNO1-hTRAIL (4026 bp) was from InvivoGen (Toulouse, France). 3-(4,5-Dimethylthiazol-2-yl)-2,5-diphenyl tetrazolium bromide (MTT) was from Sigma-Aldrich (Saint-Quentin Fallavier, France). DMEM/F12 and RPMI culture media and their supplements were from GIBCO-BRL (Cergy-Pontoise, France). Fetal calf serum (FCS) was from Perbio (Brebières, France). A549 cells (human lung carcinoma; CCL-185), and NCI-H292 (H292) cells (human lung mucoepidermoid carcinoma; CRL-1848) were obtained from ATCC-LGC (Molsheim, France). The 16HBE14o- (16HBE) cells were a generous gift from Dr D. Gruenert (California Pacific Medical Center Research Institute, San Francisco, CA, USA). Defibrinated sheep blood was from Eurobio (Les Ulis, France).

Nucleic Acid Retardation Assay

Freshly prepared lipoplexes (vide infra) at the desired N/P ratio (where N is the concentration of the lipid ammonium group and P that of nucleic acid phosphate) were analyzed by agarose (1 %) gel electrophoresis. The gel was run in a 40 mM Tris-acetate-EDTA buffer, pH 8.0 and nucleic acid was further stained using an ethidium bromide solution (0.5 µg/mL).

Dynamic Light Scattering (DLS) Measurements

The average particle size and zeta potential of lipoplexes were measured using a Zetasizer nanoZS apparatus (Malvern Instruments). All measurements were performed on freshly prepared lipoplexes (vide infra) at 25 °C and in triplicate. Data were analyzed using the multimodal number distribution software supplied with the instrument, and expressed as mean (± SD).

Hydrolytic Stability of the pro-APLs

Hydrolysis rate of pro-APLs was measured by 31P-NMR spectroscopy. Compounds were formulated as liposomes using a solvent injection technique (Gentine et al, 2012). Briefly, the pro-APLs (10 µmol) were dissolved in i-PrOH (200 µL) and then injected with a syringe with a flow rate of approximately 600 µL min-1 and a stirring speed of 400 rpm into the appropriate aqueous buffer

medium (300 µL, either HEPES 10 mM pH 7.4, or AcOK/AcOH 10 mM pH 4.5) containing 10 mM Triton X-100. The resulting preparations were complemented with buffer (400 µL) and D2O (100 µL), introduced into 5-mm NMR tubes, and 31P-NMR spectra were recorded periodically at 20 °C with a 4-s pulse cycle for quantitative measurements.

Pro-APLs Self-Assembly Properties

Self-assembly properties of APLs and pro-APLs were determined using a fluorescent probe technique (Goddard et al, 1985; Piñeiro et al, 2015). Starting from a lipid film, compounds were dispersed in H2O (2 mM, 1 mL) and diluted in a concentration range from 2 mM to 2 nM. The samples were added with pyrene (0.1 mM in DMSO, 10 µL) under vigorous stirring, and incubated in the dark for 30 min at rt. Fluorescence spectra were then recorded with a Fluoromax-4 spectrophotometer (Horiba Jobin Yvon), from 350 to 450 nm, with excitation at 330 nm. Emission spectral ratio at 375 and 387 nm (I375/I387) was expressed as the logarithm of concentration, and CMC which is the concentration above which the molecules spontaneously associate to form micelles was determined as the first break point in the fluorescence behavior of pyrene.

Hemolytic Activity

Red blood cell (RBC) leakage assay was carried out according to a previously described procedure (Evans et al, 2013). RBC were prepared from defibrinated sheep blood. Briefly, blood sample (5 mL) was centrifuged at 500 x g for 5 min. After removal of the yellow upper layer, an equal volume of PBS pH 7.4 was added to the sample, and the mixture was homogenized. This treatment (centrifugation, removal of upper layer, addition of PBS and homogenization) was repeated three times. The last homogenate was diluted to 1/25 in PBS and the resulting suspension of RBCs (140 µL) was added to lipid samples (vide supra, 10 µL) prepared at increasing concentration (from 45 µM to 3 mM) in 96-well plates. After incubation for 1 or 24 h at 37 °C and 5 % CO2, plates were centrifuged at 250 x g for 10 min to pellet intact RBC and absorbance of the supernatants (100 µL) was measured at 450 nm. Hemolysis induced by the compounds was expressed as percent, calculated from negative and 100% controls, and plotted as a function of lipid concentration. Negative control was obtained on suspensions of RBC in PBS alone. Complete hemolysis (100 % control) was obtained by adding Triton X-100® (10 µM, 10 µL) to suspensions of RBC in PBS alone. Lipid concentration inducing 50 % hemolysis (HC50) and the percentage of hemolysis provoked by 200 µM of the lipid (HA200) were graphically determined from the curve fitted to the data.

Cell Culture

All cell lines were grown in culture flasks (Becton-Dickinson) at 37 °C in a 5 % CO2 humidified chamber. Human lung carcinoma (A549) and bronchial (16HBE) epithelial cells were grown in DMEM/F12 medium containing FBS (10 %), penicillin (100 units/mL), streptomycin (100 µg/mL), and Hepes (5 mM). Lung mucoepidermoid carcinoma cells (H292) were grown in RPMI 1640 supplemented with FBS (10 %), sodium pyruvate (1 mM), L-glutamine (2 mM), penicillin (100 units/mL), streptomycin (100 µg/mL), and Hepes (10 mM). At confluency, cells were released from flasks with trypsin (0.5 % in PBS), centrifuged (4 °C, 5 min, 120 x g) counted and transferred into 96-well plates (Becton-Dickinson) in 100 µL culture medium (A549: 6,000 cells/well; H292: 9,000 cells/well; 16HBE: 12,000 cells/well) for transfection experiments, cytotoxicity assays, or IC50 determinations. Plates were maintained at 37 °C in a 5 % CO2 humidified chamber for 24 h before experiments.

Lipoplexes Preparation

Typically, the appropriate volume of a freshly prepared solution of a pro-APL compound (2 mM in EtOH) was deposited at the bottom of a 500 µL polyethylene tube and dried in a SpeedVac vacuum concentrator for 1 h. To the resulting lipid film, pCMV-Gluc DNA (40 µL at the required concentration in 5 % glucose, DNA concentration refers to phosphate content) was added. After stirring by vortex for 20 s, the preparation was allowed to stand at rt for 30 min before use.

Transfection Experiments

Cells were seeded into 96-well plates (Becton-Dickinson) at the required density (vide supra) in 100 µL of serum containing culture medium. Twenty-four hours later, freshly prepared DNA lipoplexes (10 µL, i.e. 0.4 µg DNA) were added to the wells and cells were then let to grow in the incubator without further handling for 24 h. Negative control was obtained by adding glucose 5 % (10 µL) to the wells instead of lipoplexes. Then, Gaussia luciferase production was measured by monitoring light production on an aliquot of culture supernatant (20 µL of a 1/100th dilution of supernatant prepared in non-supplemented culture medium) for 1 s upon addition of the coelenterazine substrate (50 µL, 1.5 µM) using a luminometer (Berthold Centro LB960 XS). Protein content of the culture supernatant was determined using a bicinchoninic acid (BCA) assay and luciferase activity was normalized to protein content (RLU/mg protein). Value for each sample is the mean of a triplicate determination (± SD).

Cell Viability Assay

Mitochondrial activity measurements (MTT assay) was used to assess cytotoxicity of the compounds. Lipid samples were prepared from a lipid film (3.6 µmol of compound evaporated from an ethanolic solution) hydrated with aqueous glucose 5 % and vigorously vortexed for 20 s before serial dilution in aqueous glucose 5 %. For addition of lipid samples to cell culture, part of the culture medium in the culture plates prepared the day before (25 µL) was removed and replaced by lipid samples (25 µL). Negative control was obtained by adding glucose 5 % to the cells instead of lipoplexes. After a 24-h incubation period at 37 °C, culture supernatant was removed, cells were carefully washed with PBS and MTT (100 µL, 0.5 mg/mL) in complete culture medium was added. After a 1-h incubation period at 37 °C, the MTT solution was removed and DMSO (100 µL) was added to lyse cells and dissolve reduced MTT. Intensity of MTT reduction was then evaluated by measuring absorbance at 492 nm. Viability of cells treated with lipids or lipoplexes was expressed as the percentage of the absorbance measured in untreated cells. Value for each sample is the mean of triplicate determinations (± SD). IC50 values were determined within the 95 % confidence interval using nonlinear regression analysis with the GraphPad Prism software (GraphPad Software, San Diego, CA, USA).

Statistical Analysis

All data are representative of a triplicate determination and are presented as the mean ± SD. Statistical significance between treatments was assessed by one-way analysis of variance (ANOVA) followed by the Dunnett multiple comparison test, using the Kaleidagraph 4.5 software (Synergy Software, Reading, PA, USA). Data were considered as statistically significant for p value less than 0.05 (*** $p < 0.001$, ** $p < 0.01$, * $p < 0.05$).

Supplementary Figures

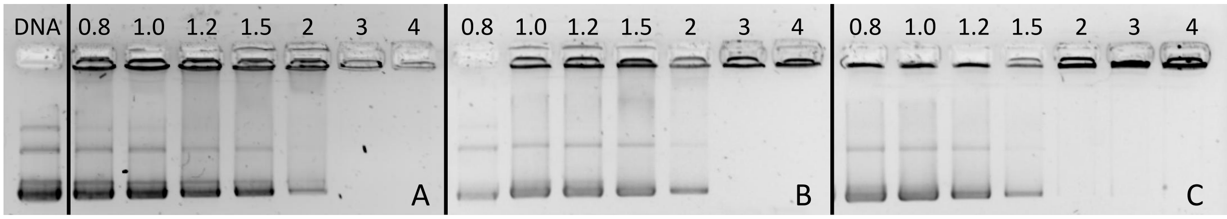

Figure S1. Representative agarose gels obtained for determining pDNA binding ability of pro-APLs (A: $\mathbf{M}_{\mathbf{E12}}$; B: $\mathbf{P}_{\mathbf{E12}}$; C: $\mathbf{E}_{\mathbf{E12}}$). Lipoplexes were prepared at increasing N/P ratio (0 to 4) by mixing 0.8 µg of plasmid DNA (pCMV-Gluc) and pro-APLs in 10 mM Hepes buffer. After an incubation period of 20 min, samples (25 µL) were analyzed by 1 % agarose gel electrophoresis using Tris-acetate-EDTA buffer. DNA was visualized after ethidium bromide staining.

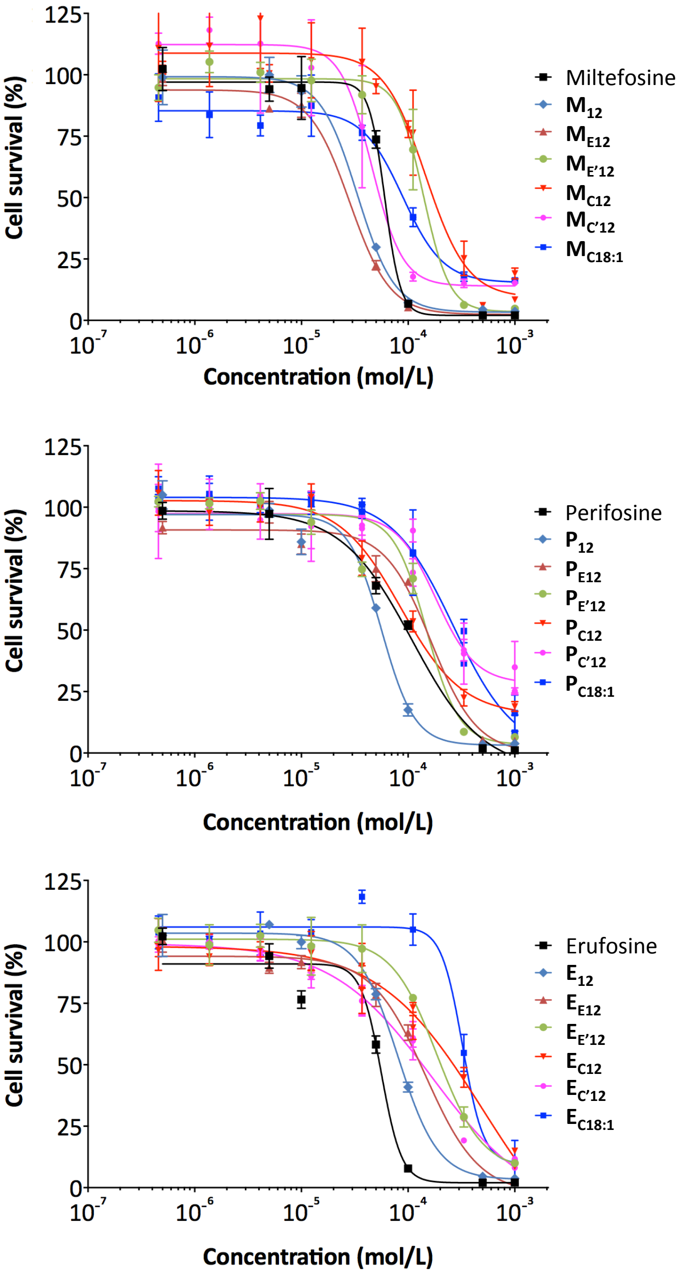

Figure S2. Survival of A549 cells treated with increasing concentrations of APLs and pro-APLs.

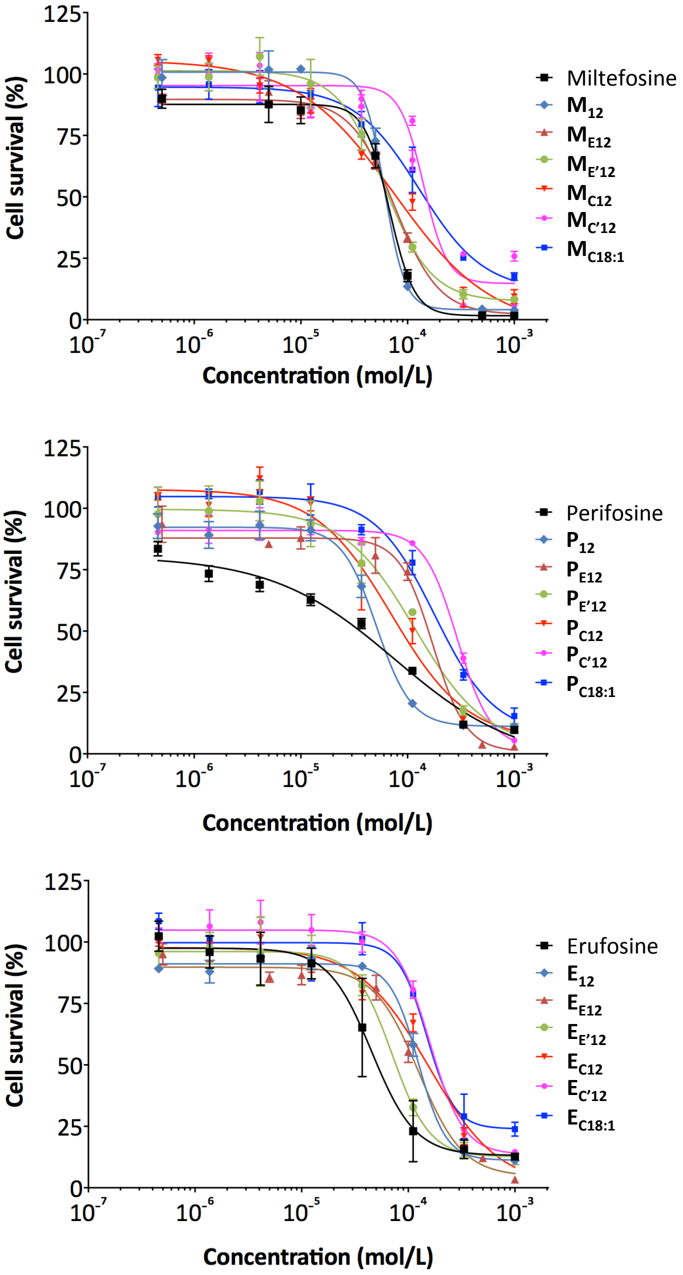

Figure S3. Survival of H292 cells treated with increasing concentrations of APLs and pro-APLs.

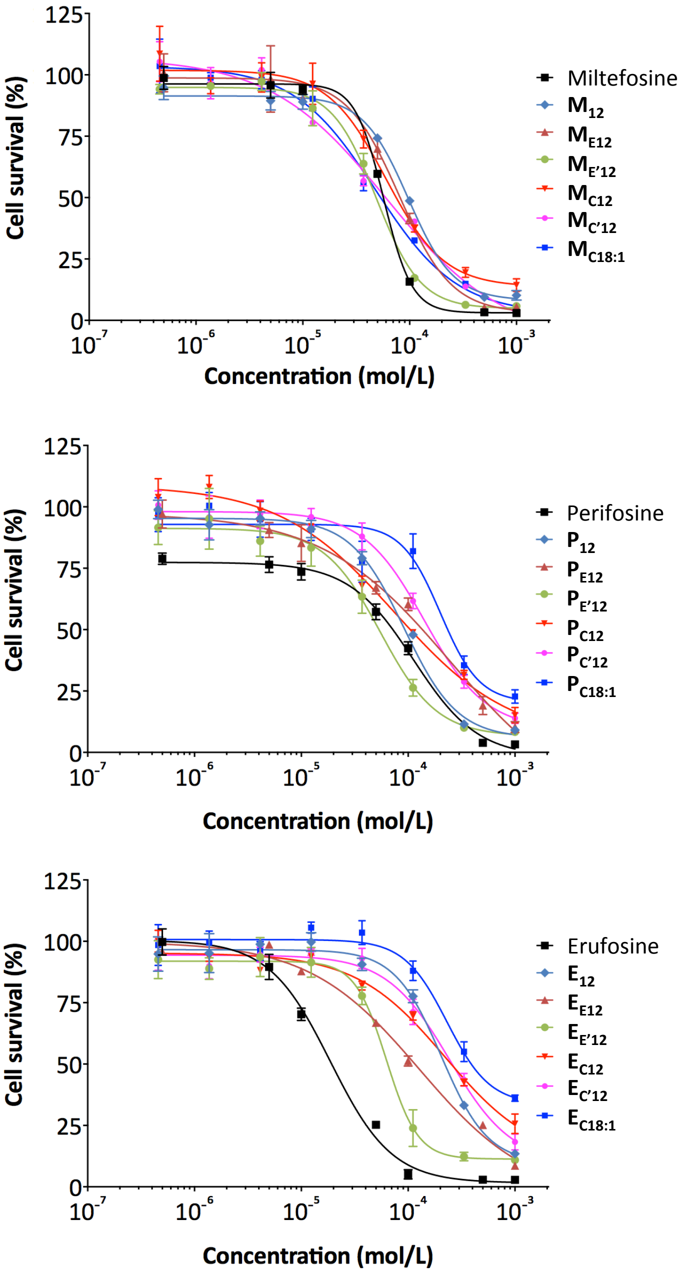

Figure S4. Survival of 16HBE cells treated with increasing concentrations of APLs and pro-APLs.

Supplementary Table

Table S1. Particle size and zeta potential (ζ) of lipoplexes obtained from pro-APLs and pCMV-Gluc, as measured by DLS. Lipoplexes were prepared at 25 °C, in 5 % glucose, at a N/P ratio of 3 and with 1 molar equivalent of DOPE. Data for the erufosine prodrugs are reported from the literature (Gaillard et al., 2019). Data are the mean of three independent measurements (± SD).

Compound	Particle size (nm)	ζ (mV)
$\mathbf{M_{12}}$	121 ± 13	+ 46 ± 1
$\mathbf{M_{E12}}$	178 ± 8	+ 43 ± 1
$\mathbf{M_{E'12}}$	86 ± 3	+ 40 ± 1
$\mathbf{M_{C12}}$	186 ± 48	+ 31 ± 1
$\mathbf{M_{C'12}}$	91 ± 3	+ 42 ± 1
$\mathbf{M_{C18:1}}$	275 ± 19	+ 39 ± 1
$\mathbf{P_{12}}$	348 ± 43	+ 16 ± 1
$\mathbf{P_{E12}}$	330 ± 76	+ 44 ± 2
$\mathbf{P_{E'12}}$	610 ± 54	+ 37 ± 2
$\mathbf{P_{C12}}$	576 ± 41	+ 44 ± 2
$\mathbf{P_{C'12}}$	724 ± 56	+ 44 ± 1
$\mathbf{P_{C18:1}}$	489 ± 130	+ 14 ± 3
$\mathbf{E_{12}}$	194 ± 28	+ 42 ± 1
$\mathbf{E_{E12}}$	141 ± 5	+ 48 ± 2
$\mathbf{E_{E'12}}$	149 ± 15	+ 39 ± 2
$\mathbf{E_{C12}}$	117 ± 15	+ 40 ± 1
$\mathbf{E_{C'12}}$	92 ± 10	+ 54 ± 2
$\mathbf{E_{C18:1}}$	611 ± 12	+ 47 ± 3

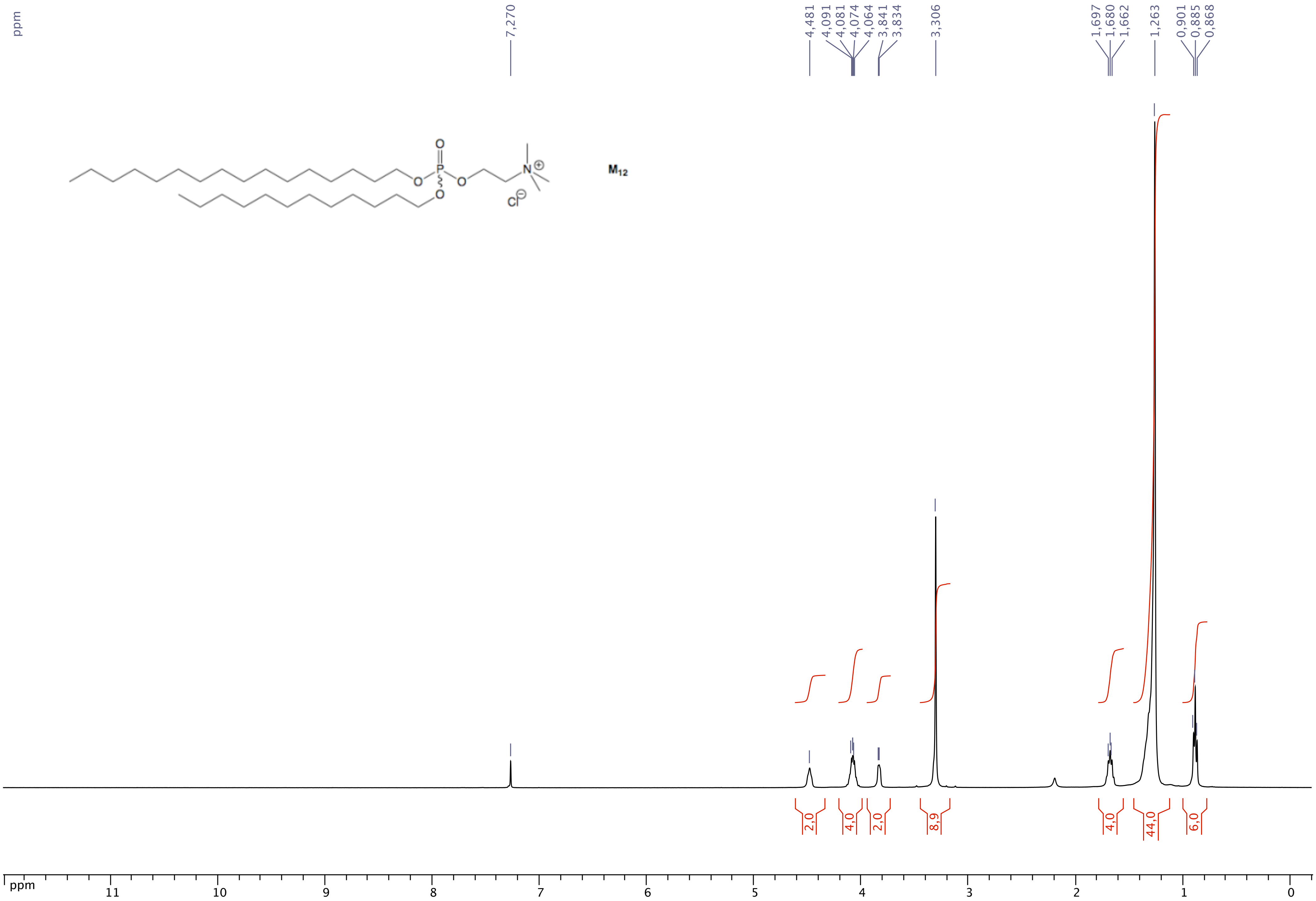
ppm
7,270
4,481
4,091
4,081
4,074
4,064
3,841
3,834
3,306
1,697
1,680
1,662
1,263
0,901
0,885
0,868
O
P
O
O
O
N
Cl
M12
2,0
4,0
2,0
8,9
4,0
44,0
6,0
ppm
11
10
9
8
7
6
5
4
3
2
1
0

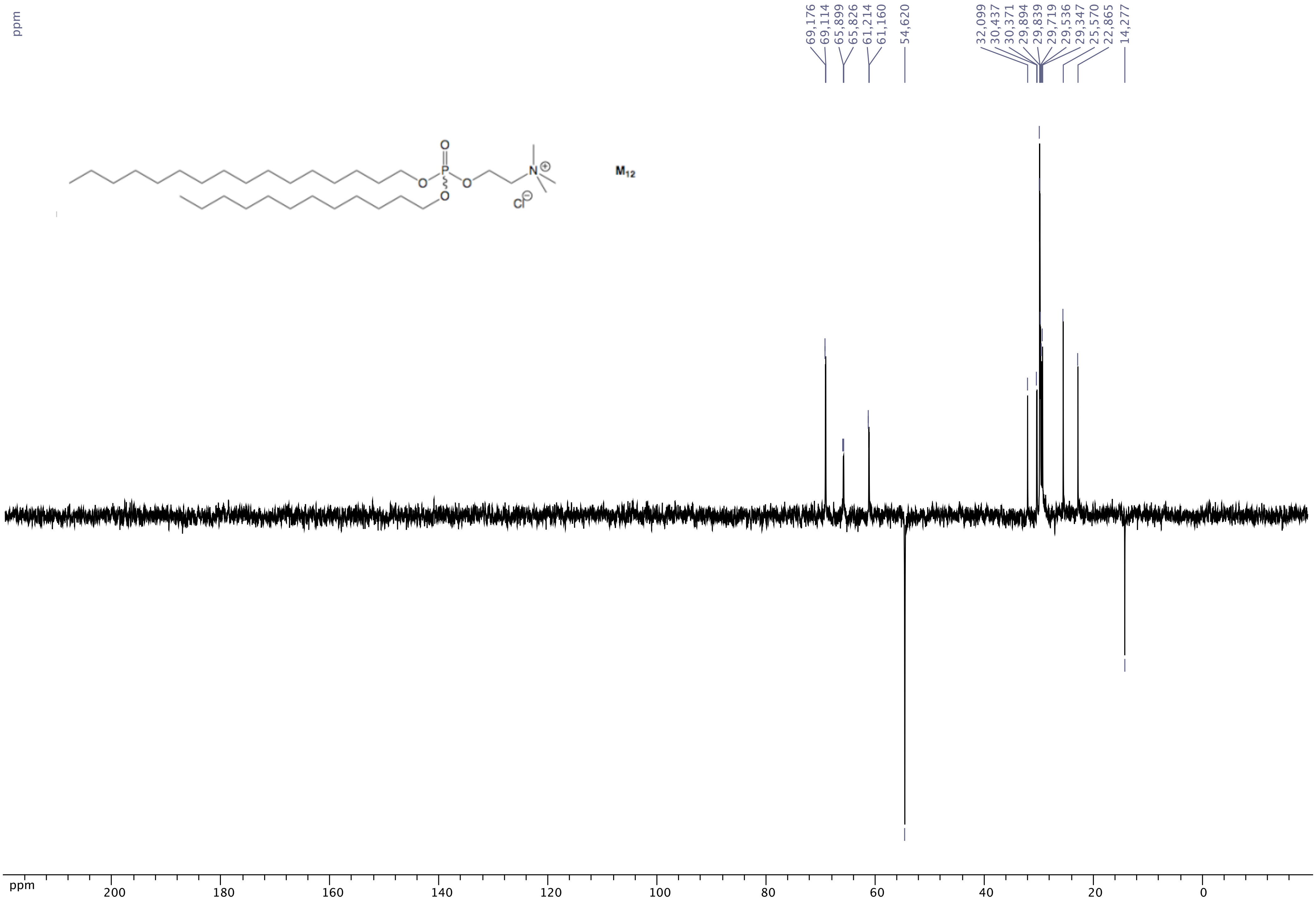
ppm
69,176
69,114
65,899
65,826
61,214
61,160
54,620
32,099
30,437
30,371
29,894
29,839
29,719
29,536
29,347
25,570
22,865
14,277
M12
ppm
200
180
160
140
120
100
80
60
40
20
0

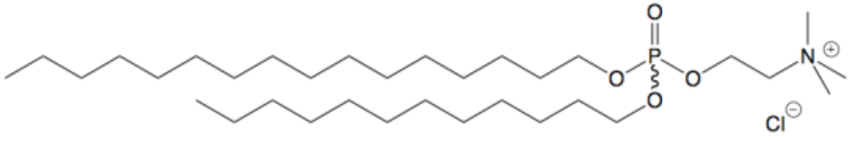

M_{12}

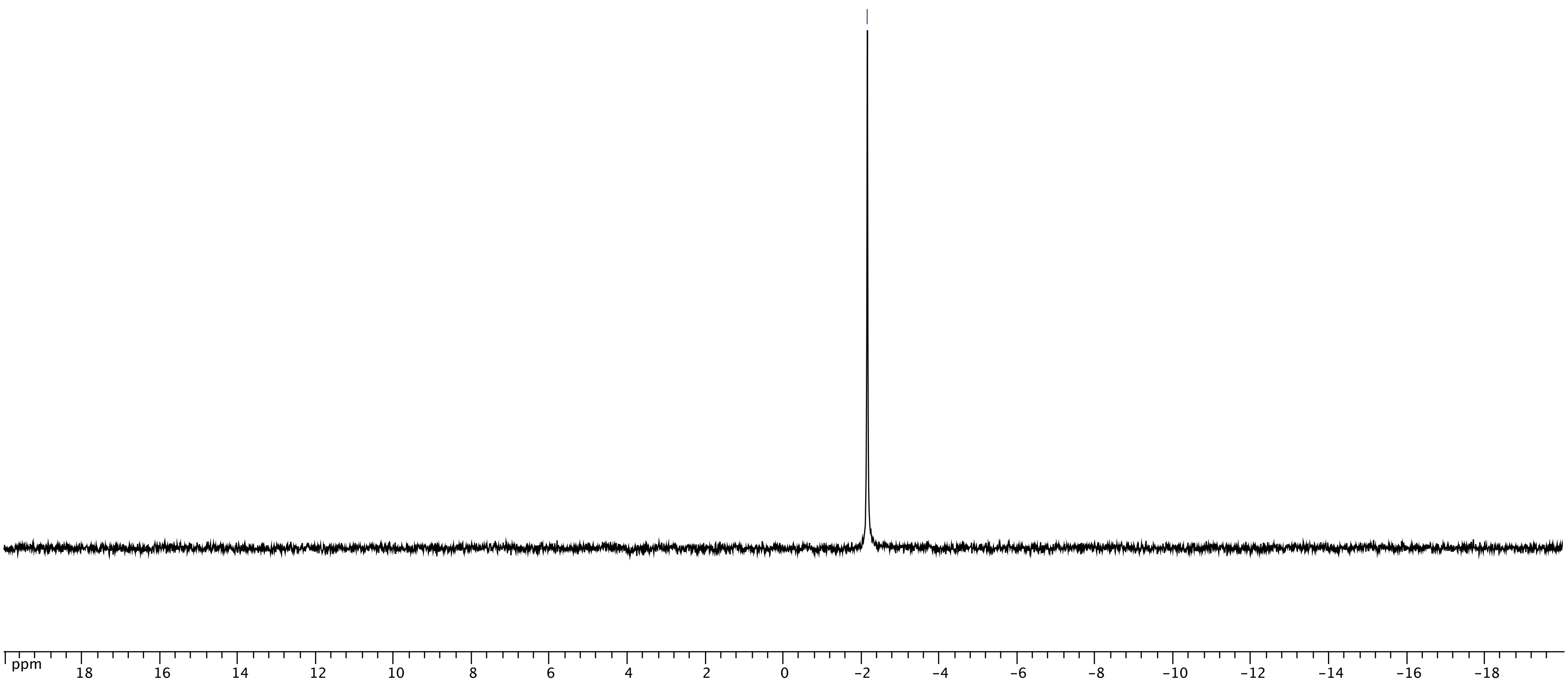

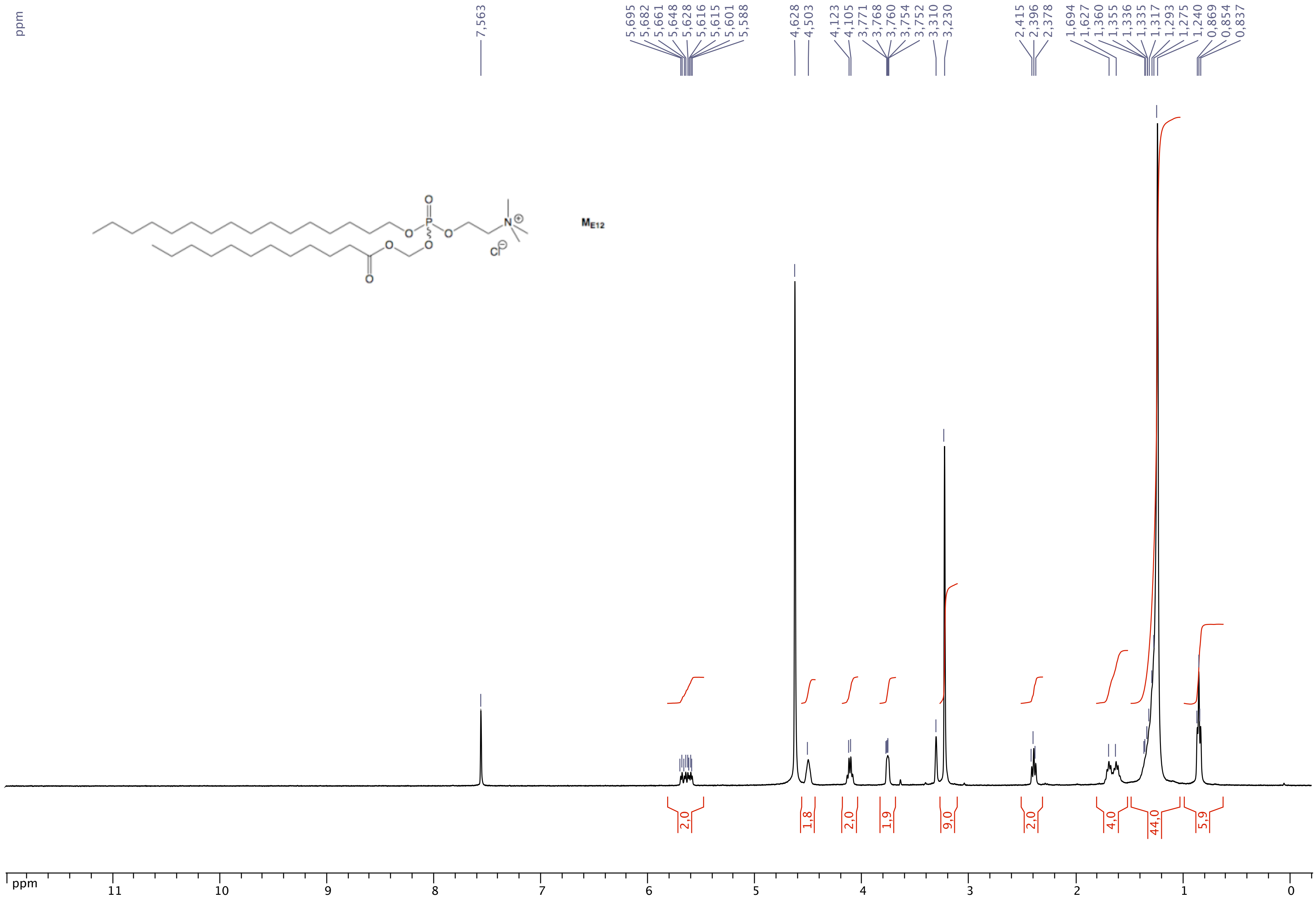

ppm
7,563
5,695
5,682
5,661
5,648
5,628
5,616
5,615
5,601
5,588
4,628
4,503
4,123
4,105
3,771
3,768
3,760
3,754
3,752
3,310
3,230
2,415
2,396
2,378
1,694
1,627
1,360
1,355
1,336
1,335
1,317
1,293
1,275
1,240
0,869
0,854
0,837
O
P
N
Cl
M$_{E12}$
2,0
1,8
2,0
1,9
9,0
2,0
4,0
44,0
5,9
ppm
11
10
9
8
7
6
5
4
3
2
1
0

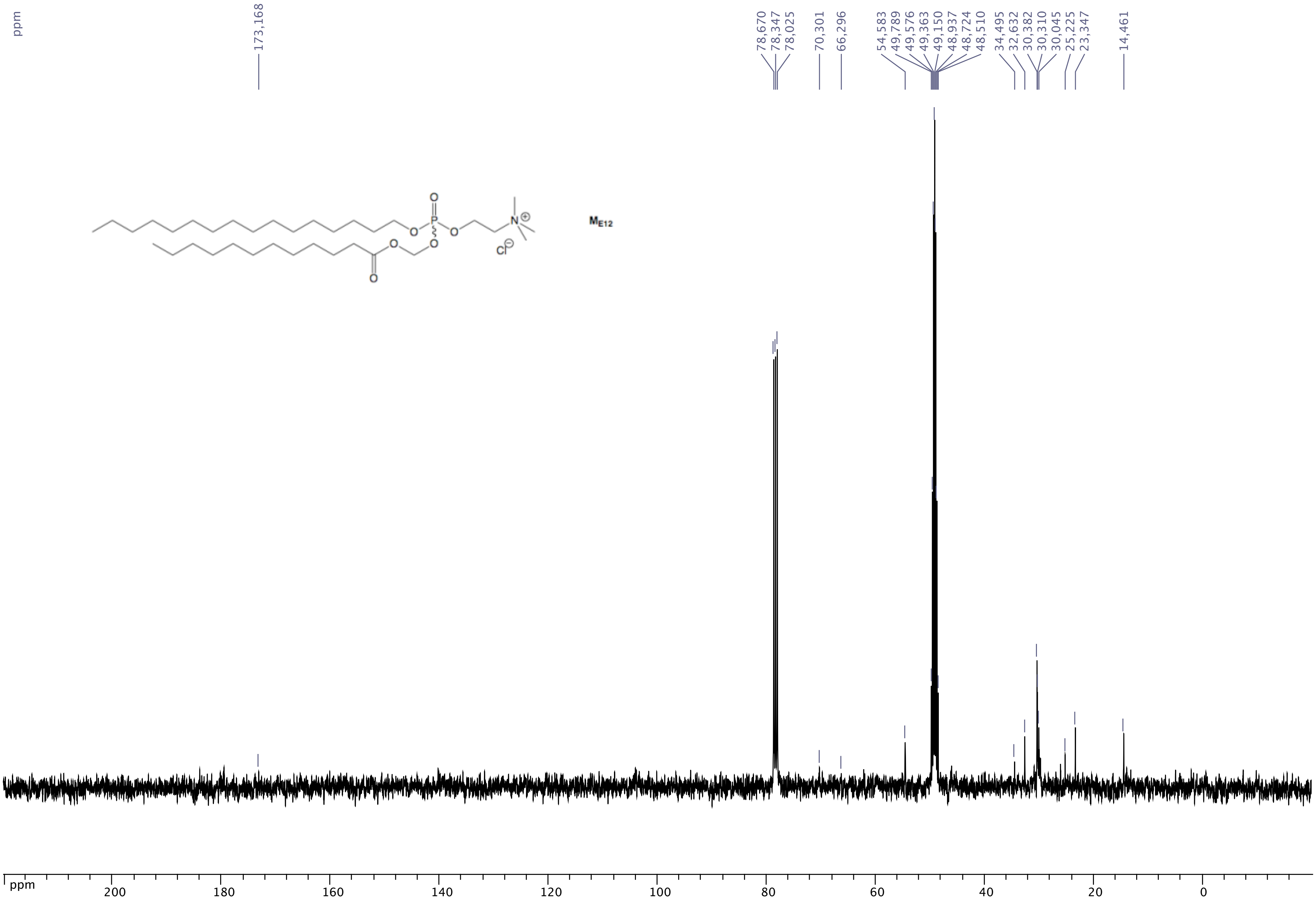
ppm
173,168
78,670
78,347
78,025
70,301
66,296
54,583
49,789
49,576
49,363
49,150
48,937
48,724
48,510
34,495
32,632
30,382
30,310
30,045
25,225
23,347
14,461
M$_{E12}$
Cl
ppm
200
180
160
140
120
100
80
60
40
20
0

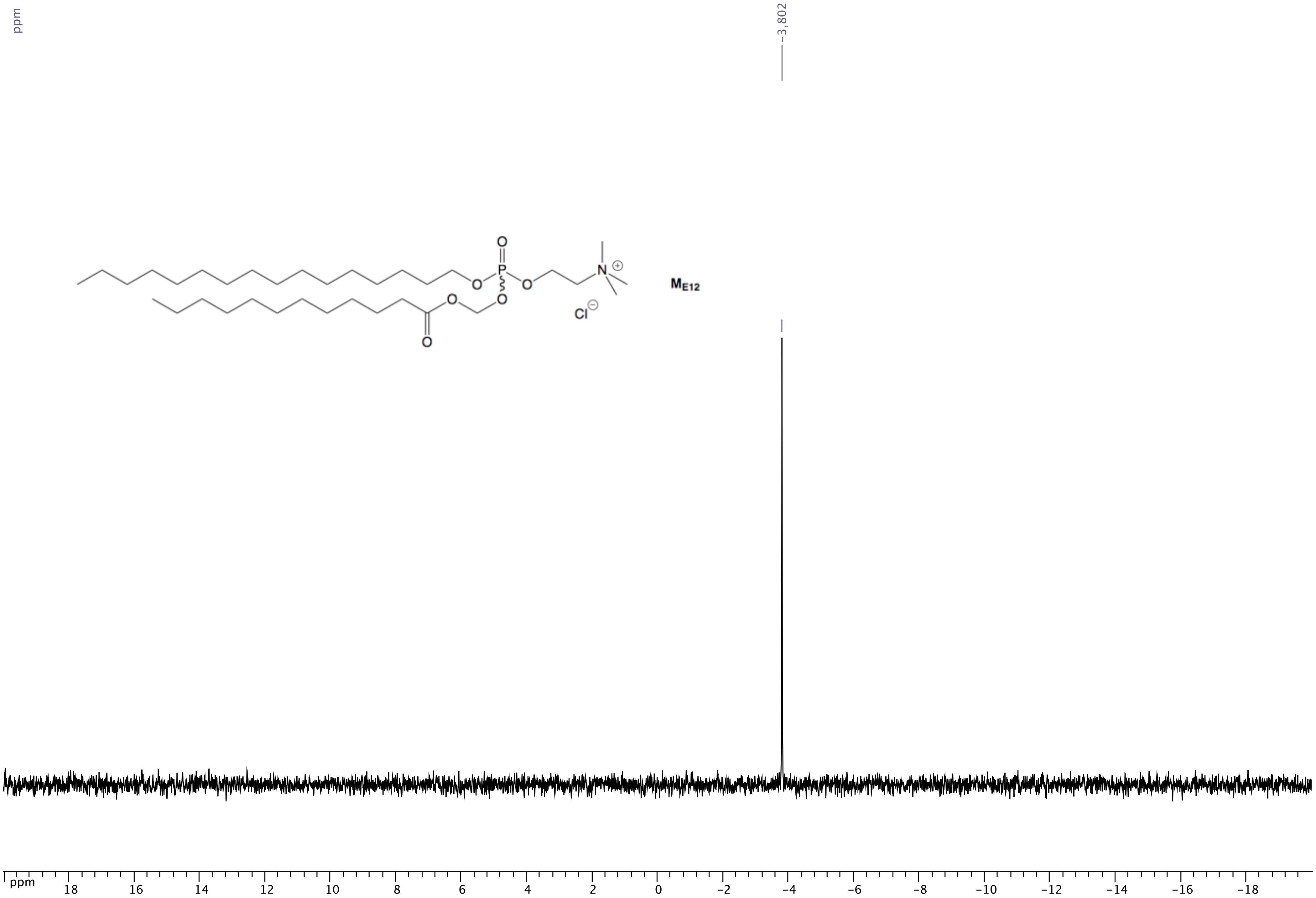

ppm
-3,802
O
P
O
O
O
O
N
Cl
O
M_{E12}
ppm
18
16
14
12
10
8
6
4
2
0
−2
−4
−6
−8
−10
−12
−14
−16
−18

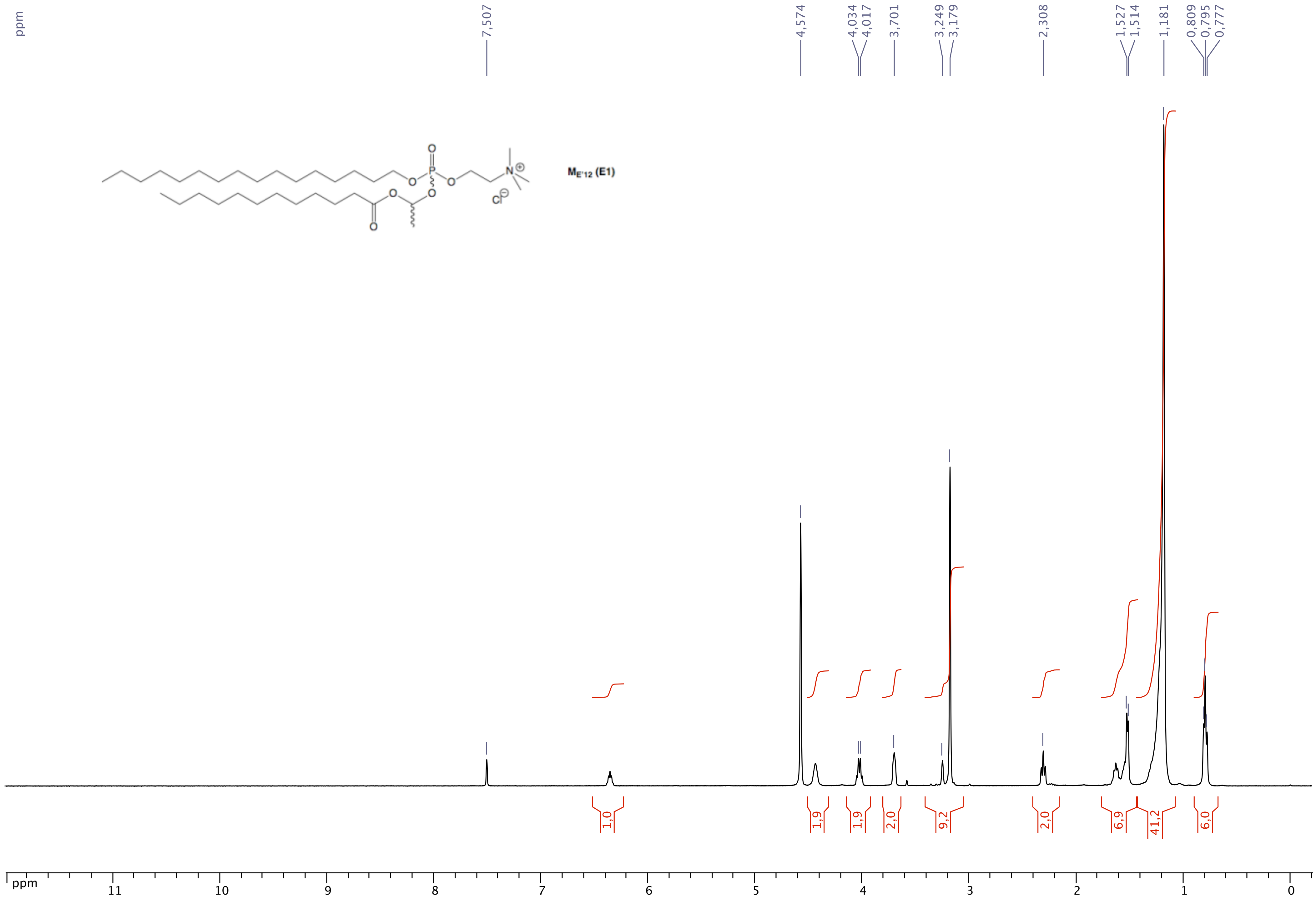
ppm
7,507
4,574
4,034
4,017
3,701
3,249
3,179
2,308
1,527
1,514
1,181
0,809
0,795
0,777
O
P
O
O
O
N
O
O
O
Cl
1,0
1,9
1,9
2,0
9,2
2,0
6,9
41,2
6,0
ppm
11
10
9
8
7
6
5
4
3
2
1
0

$M_{E'12}$ (E1)

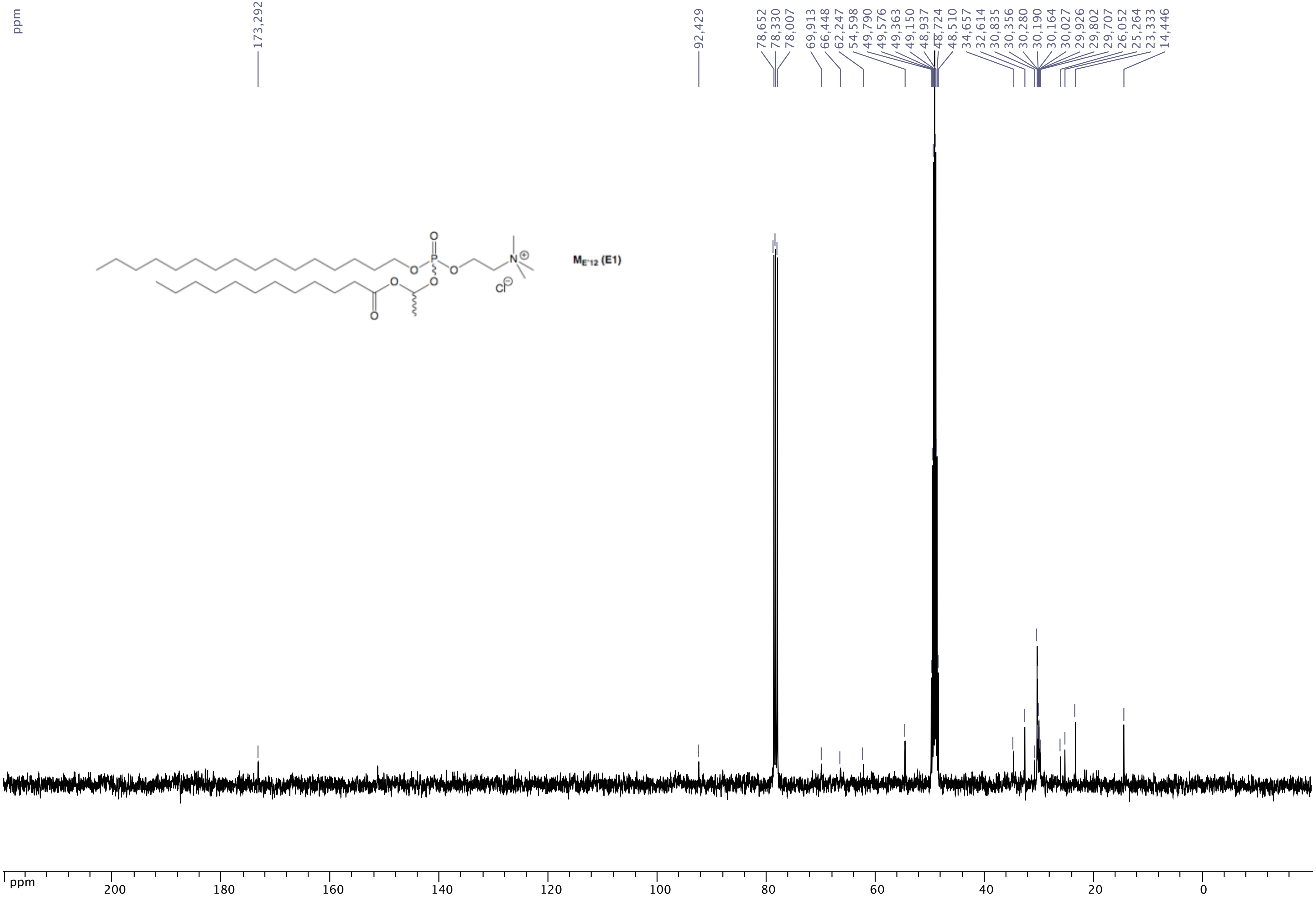
ppm
173,292
92,429
78,652
78,330
78,007
69,913
66,448
62,247
54,598
49,790
49,576
49,363
49,150
48,937
48,724
48,510
34,657
32,614
30,835
30,356
30,280
30,190
30,164
30,027
29,926
29,802
29,707
26,052
25,264
23,333
14,446
M_E'12 (E1)
ppm
200
180
160
140
120
100
80
60
40
20
0

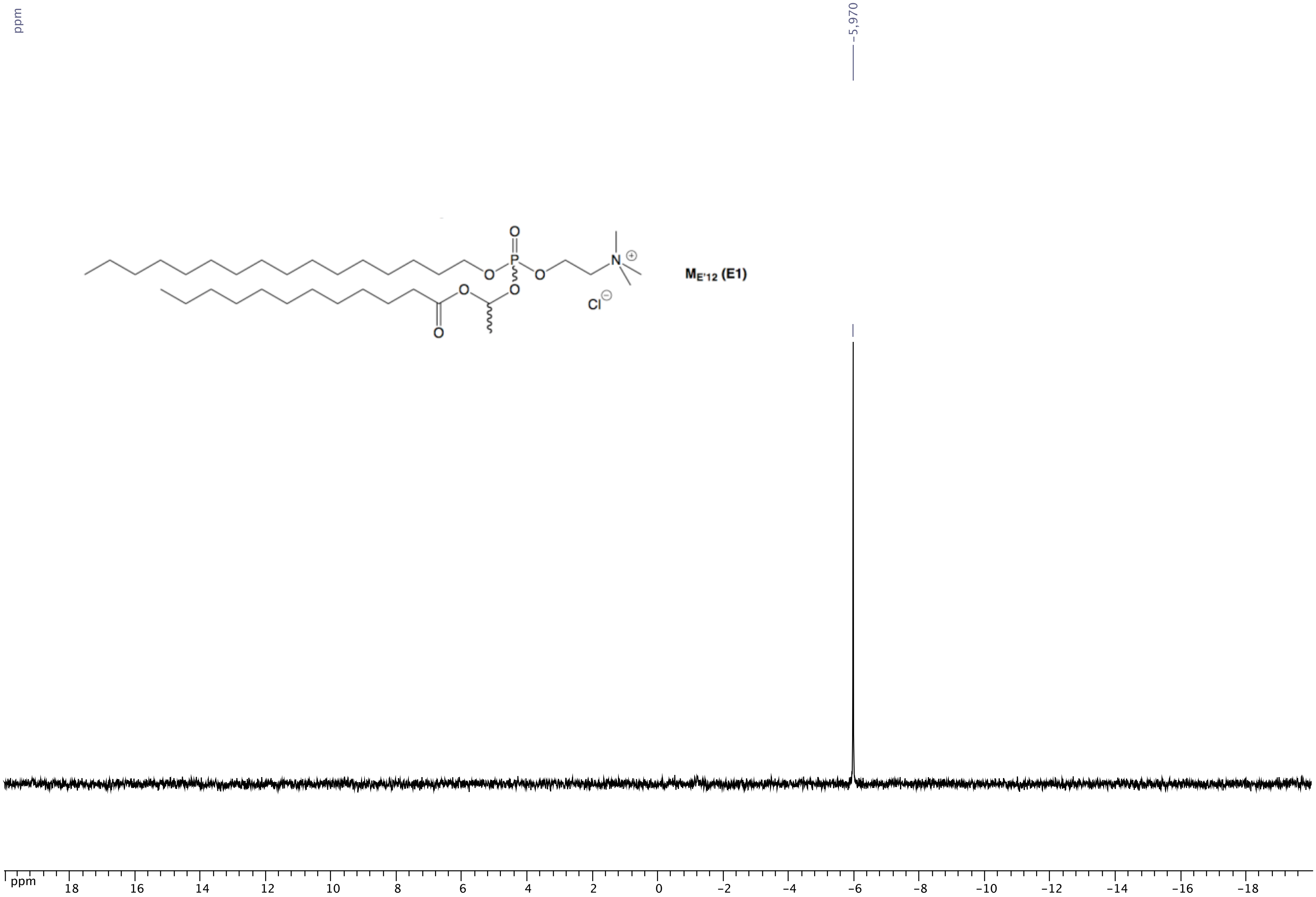

ppm
-5,970
$M_{E'12}$ (E1)
ppm
18
16
14
12
10
8
6
4
2
0
−2
−4
−6
−8
−10
−12
−14
−16
−18

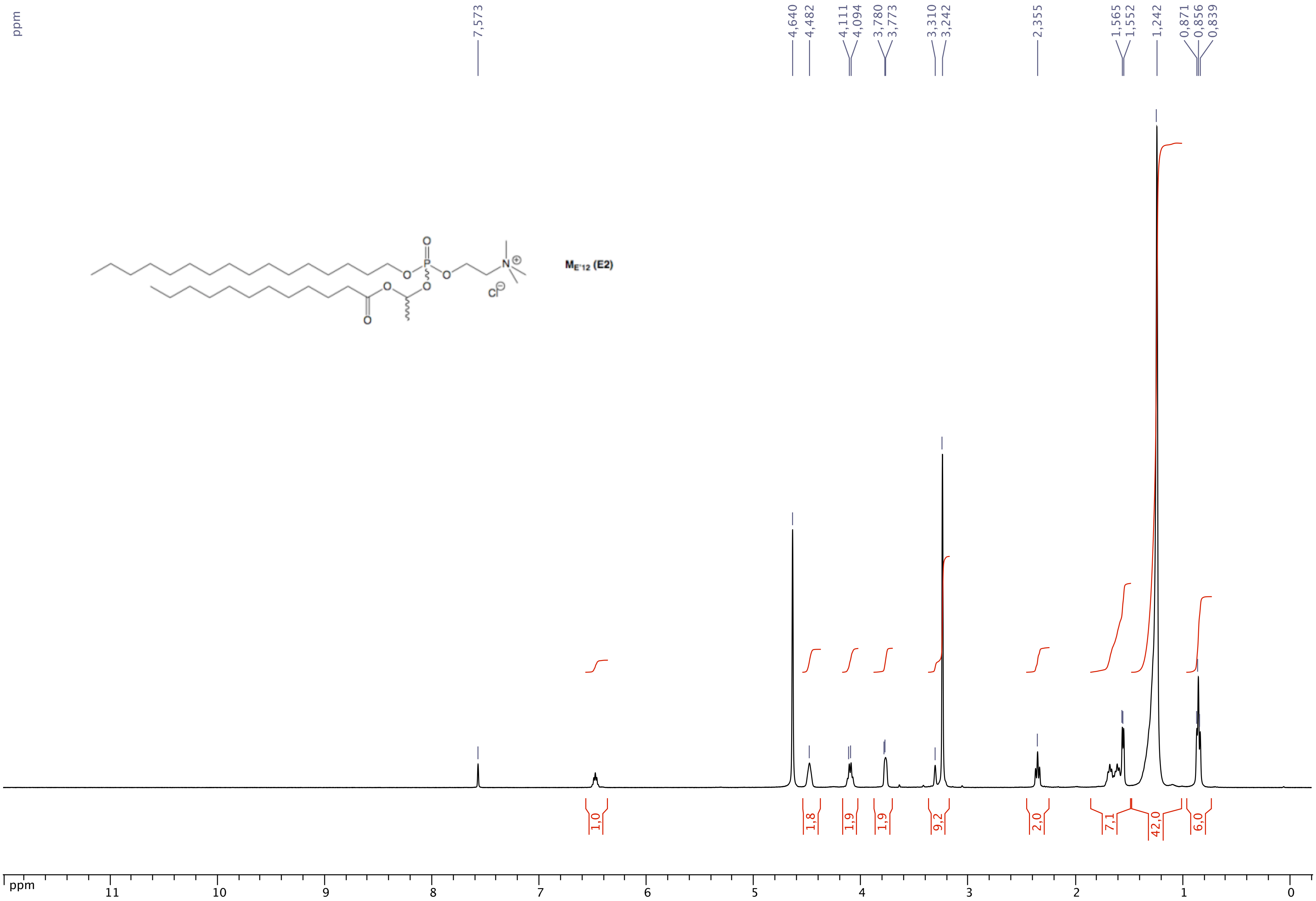
ppm
7,573
4,640
4,482
4,111
4,094
3,780
3,773
3,310
3,242
2,355
1,565
1,552
1,242
0,871
0,856
0,839
O
P
O
O
O
N
Cl
O
O
M_E'12 (E2)
1,0
1,8
1,9
1,9
9,2
2,0
7,1
42,0
6,0
ppm
11
10
9
8
7
6
5
4
3
2
1
0

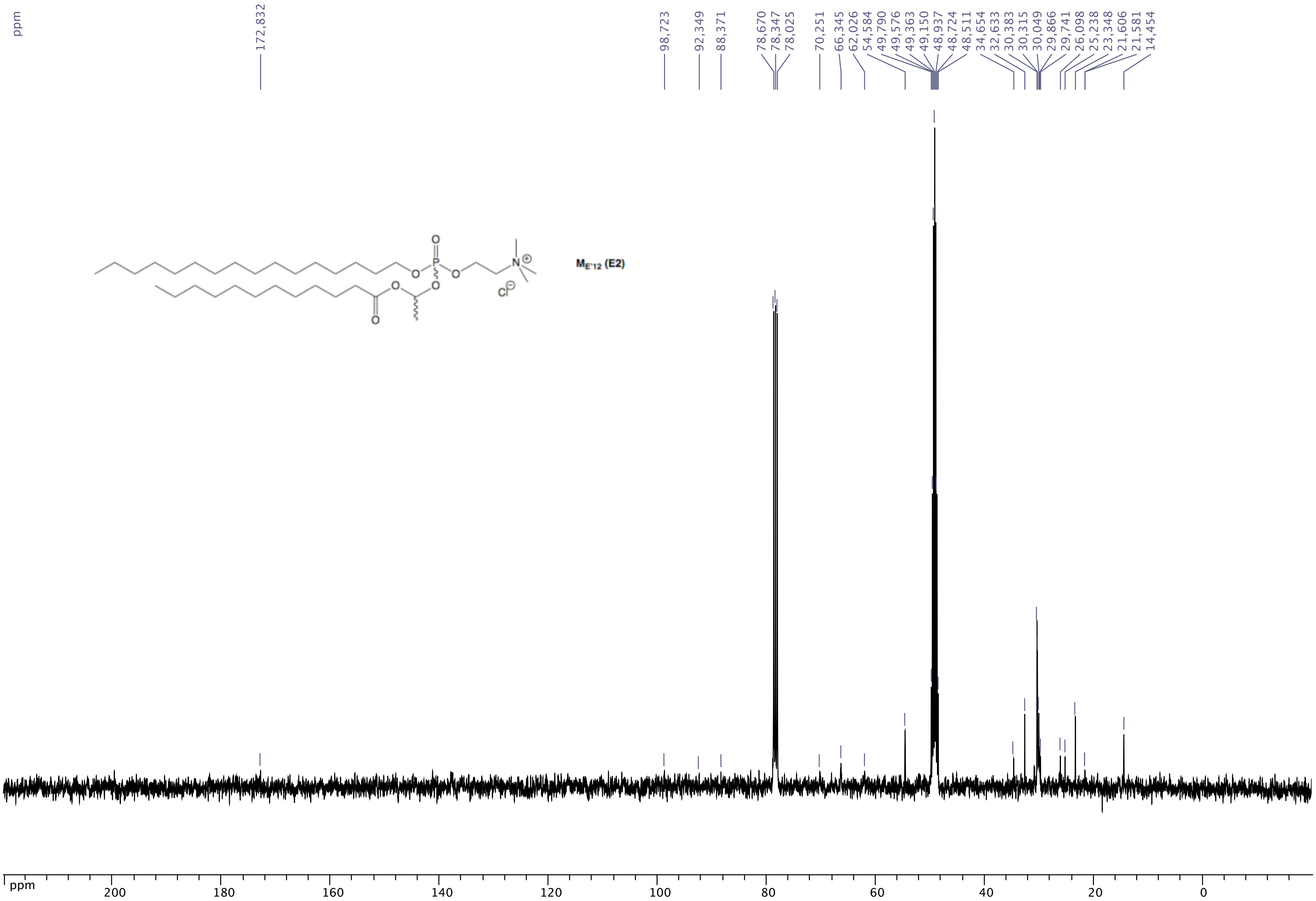

ppm
172,832
98,723
92,349
88,371
78,670
78,347
78,025
70,251
66,345
62,026
54,584
49,790
49,576
49,363
49,150
48,937
48,724
48,511
34,654
32,633
30,383
30,315
30,049
29,866
29,741
26,098
25,238
23,348
21,606
21,581
14,454
M$_{E'12}$ (E2)
ppm
200
180
160
140
120
100
80
60
40
20
0

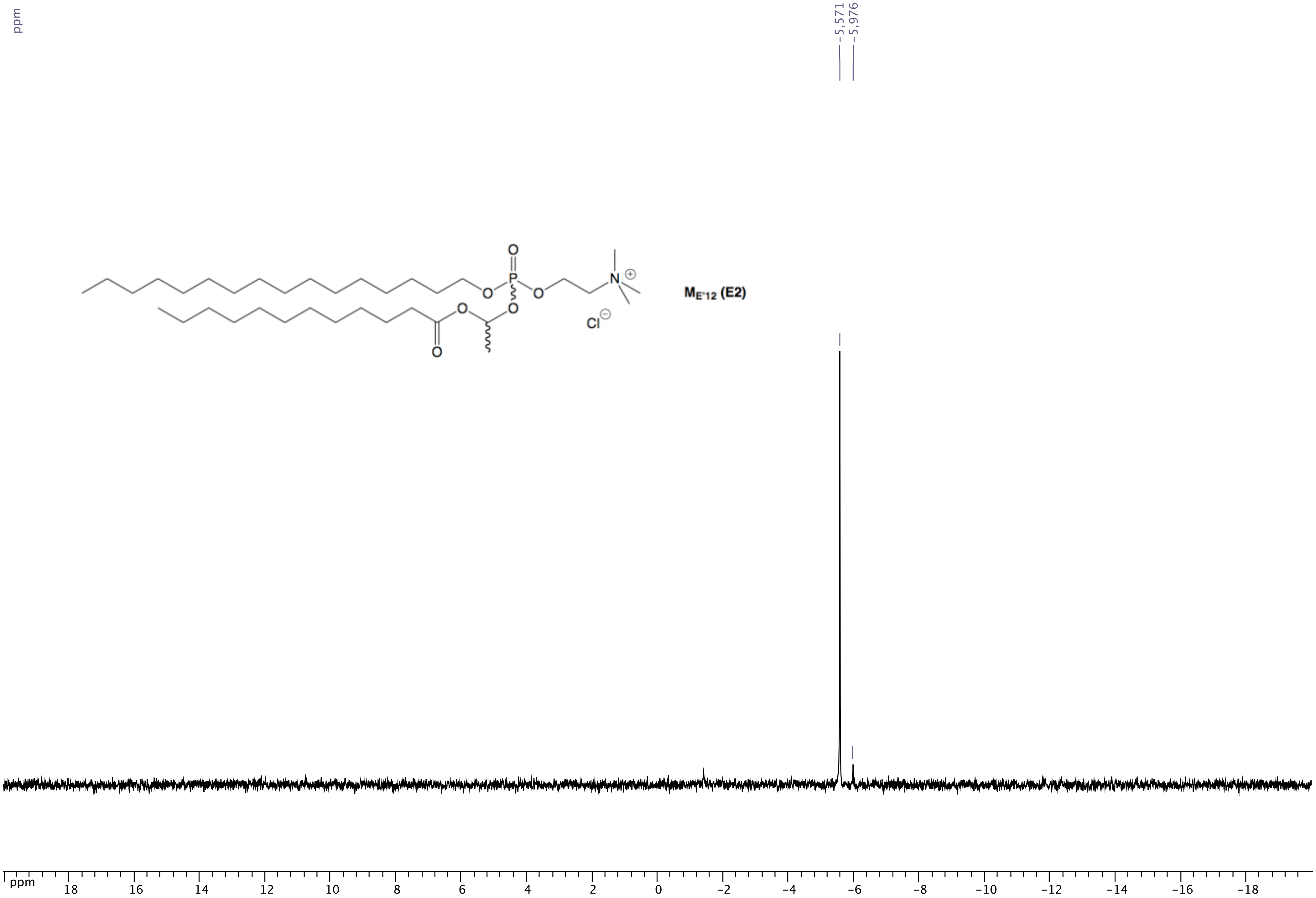

$M_{E'12}$ (E2)

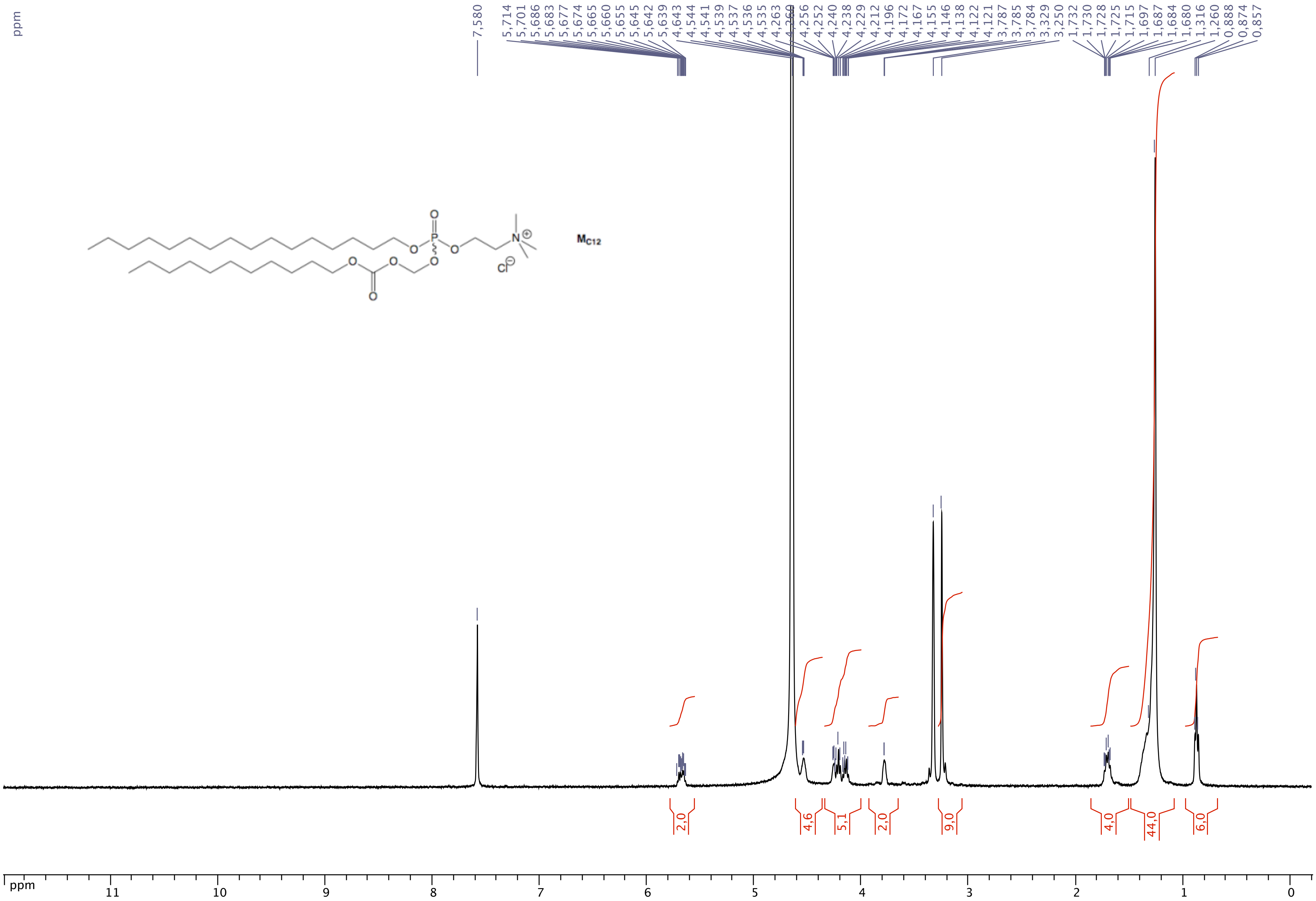
ppm
7,580
5,714
5,701
5,686
5,683
5,677
5,674
5,665
5,660
5,655
5,645
5,642
5,639
4,643
4,544
4,541
4,539
4,537
4,536
4,535
4,263
4,256
4,252
4,240
4,238
4,229
4,212
4,196
4,172
4,167
4,155
4,146
4,138
4,122
4,121
3,787
3,785
3,784
3,329
3,250
1,732
1,730
1,728
1,725
1,715
1,697
1,687
1,684
1,680
1,316
1,260
0,888
0,874
0,857
M$_{C12}$
2,0
4,6
5,1
2,0
9,0
4,0
44,0
6,0
ppm
11
10
9
8
7
6
5
4
3
2
1
0

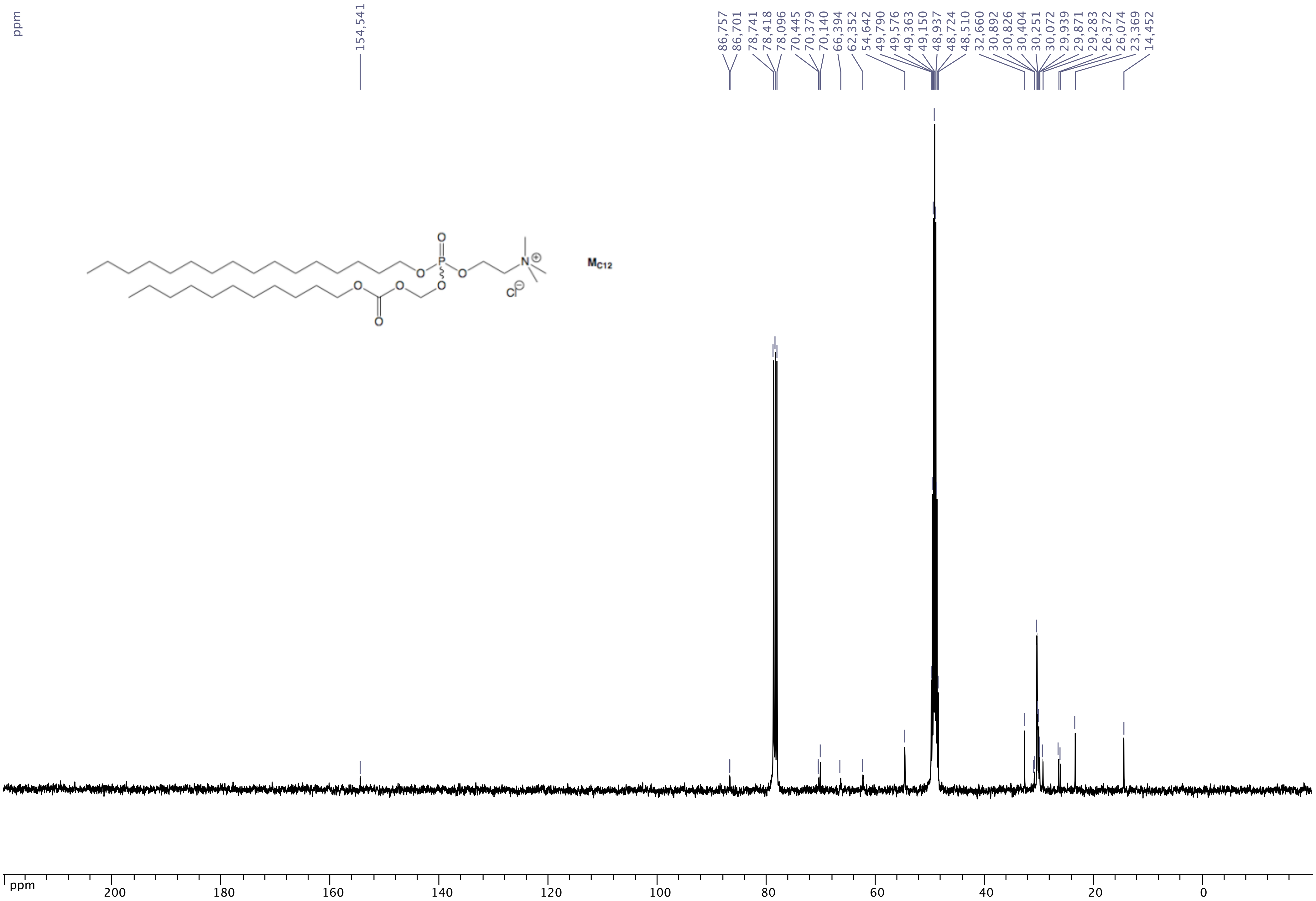
ppm
154,541
86,757
86,701
78,741
78,418
78,096
70,445
70,379
70,140
66,394
62,352
54,642
49,790
49,576
49,363
49,150
48,937
48,724
48,510
32,660
30,892
30,826
30,404
30,251
30,072
29,939
29,871
29,283
26,372
26,074
23,369
14,452
M_{C12}
ppm
200
180
160
140
120
100
80
60
40
20
0

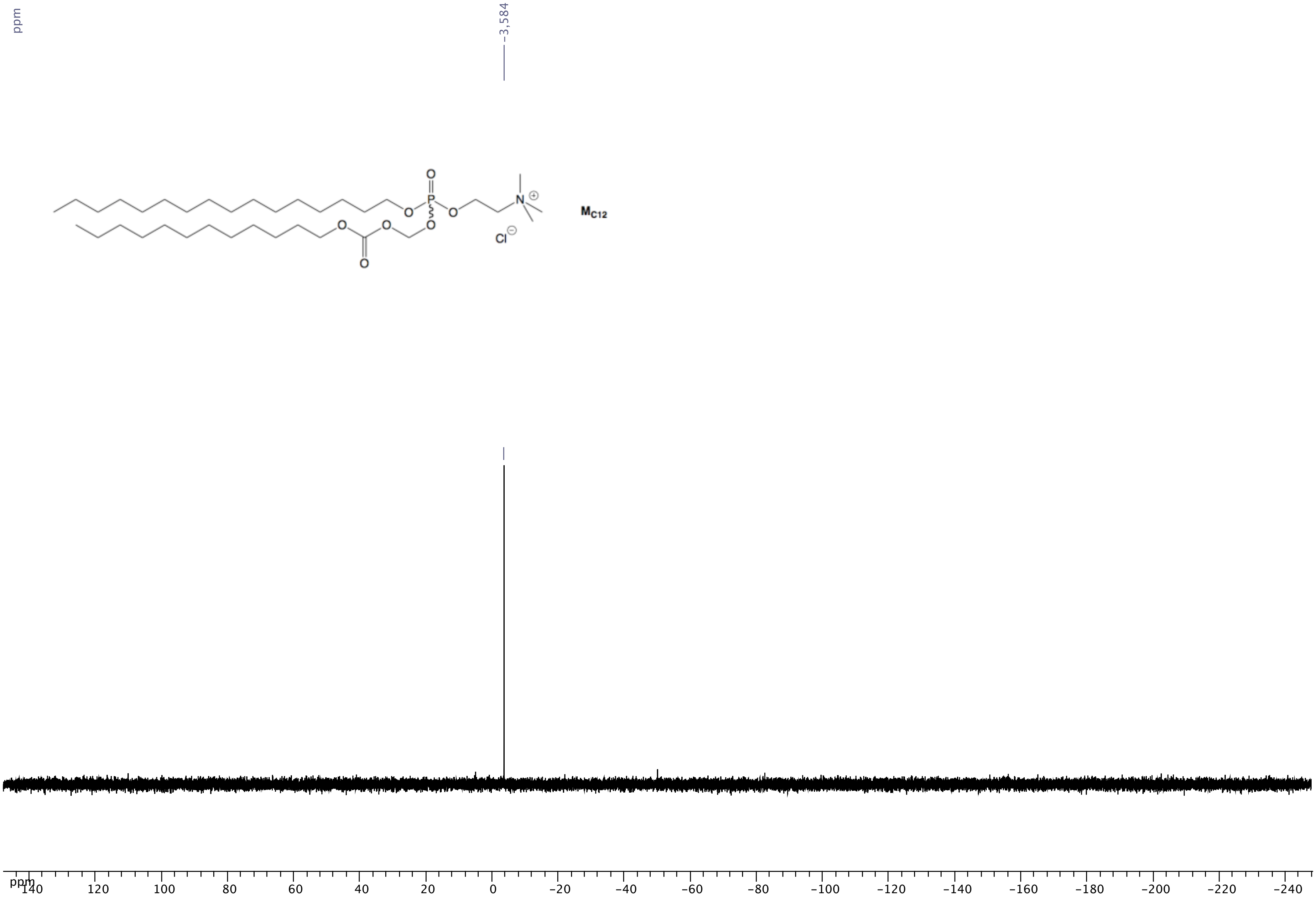

ppm
−3,584
M_C12
Cl
ppm
140
120
100
80
60
40
20
0
−20
−40
−60
−80
−100
−120
−140
−160
−180
−200
−220
−240

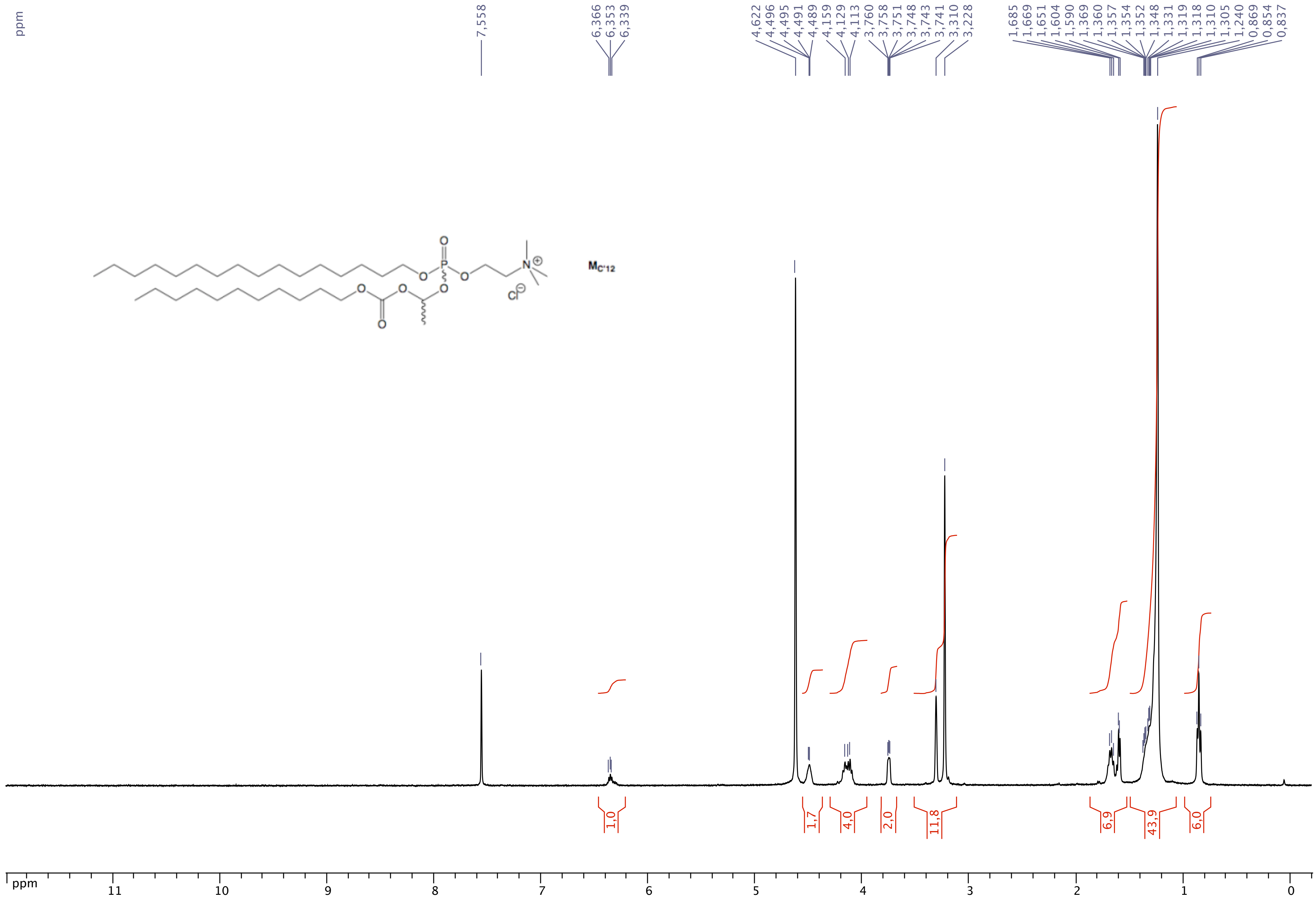
ppm
7,558
6,366
6,353
6,339
4,622
4,496
4,495
4,491
4,489
4,159
4,129
4,113
3,760
3,758
3,751
3,748
3,743
3,741
3,310
3,228
1,685
1,669
1,651
1,604
1,590
1,369
1,360
1,357
1,354
1,352
1,348
1,331
1,319
1,318
1,310
1,305
1,240
0,869
0,854
0,837
$M_{C'12}$
1,0
1,7
4,0
2,0
11,8
6,9
43,9
6,0
ppm
11
10
9
8
7
6
5
4
3
2
1
0

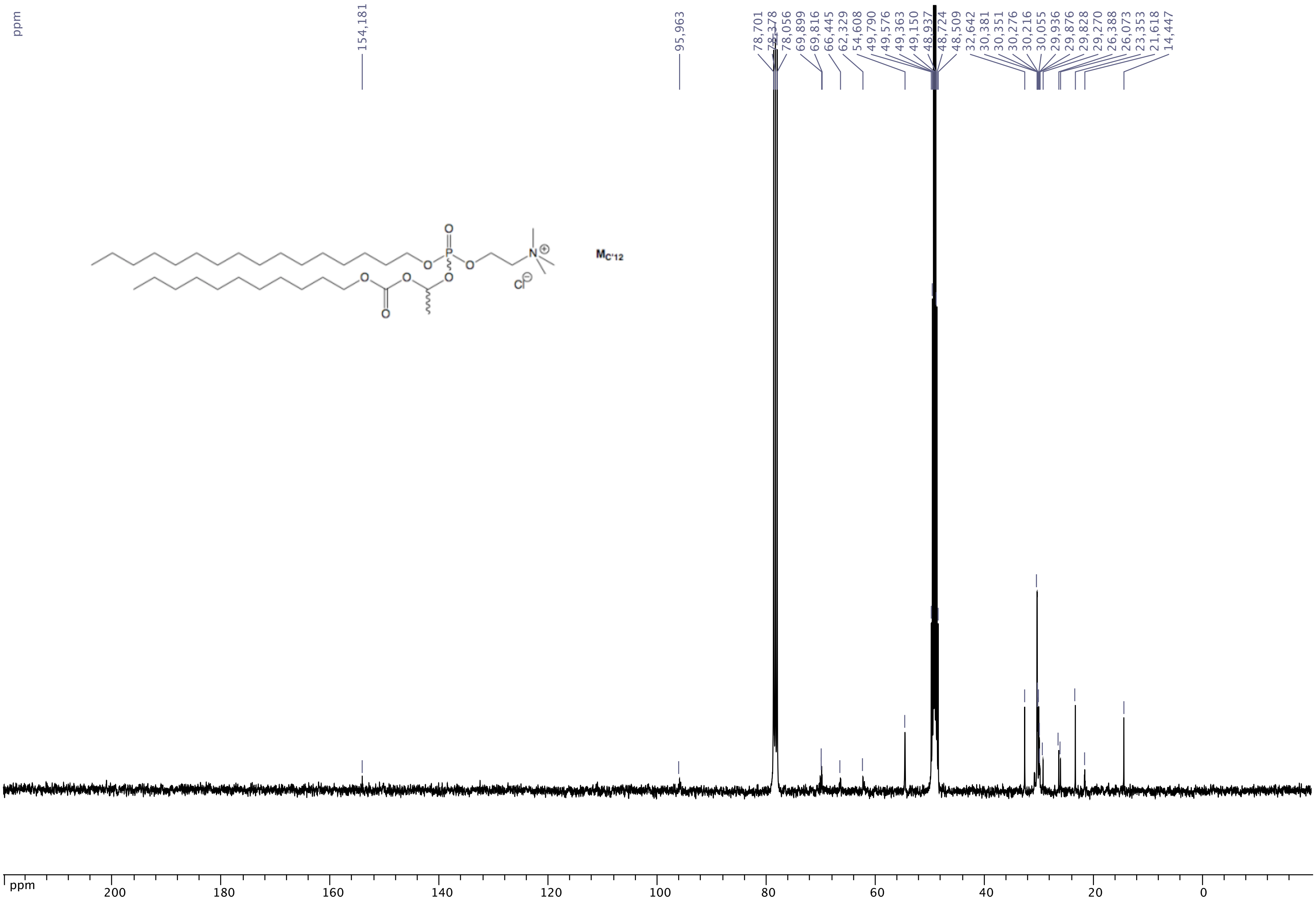
M_C'12
ppm
154,181
95,963
78,701
78,378
78,056
69,899
69,816
66,445
62,329
54,608
49,790
49,576
49,363
49,150
48,937
48,724
48,509
32,642
30,381
30,351
30,276
30,216
30,055
29,936
29,876
29,828
29,270
26,388
26,073
23,353
21,618
14,447
ppm
200
180
160
140
120
100
80
60
40
20
0

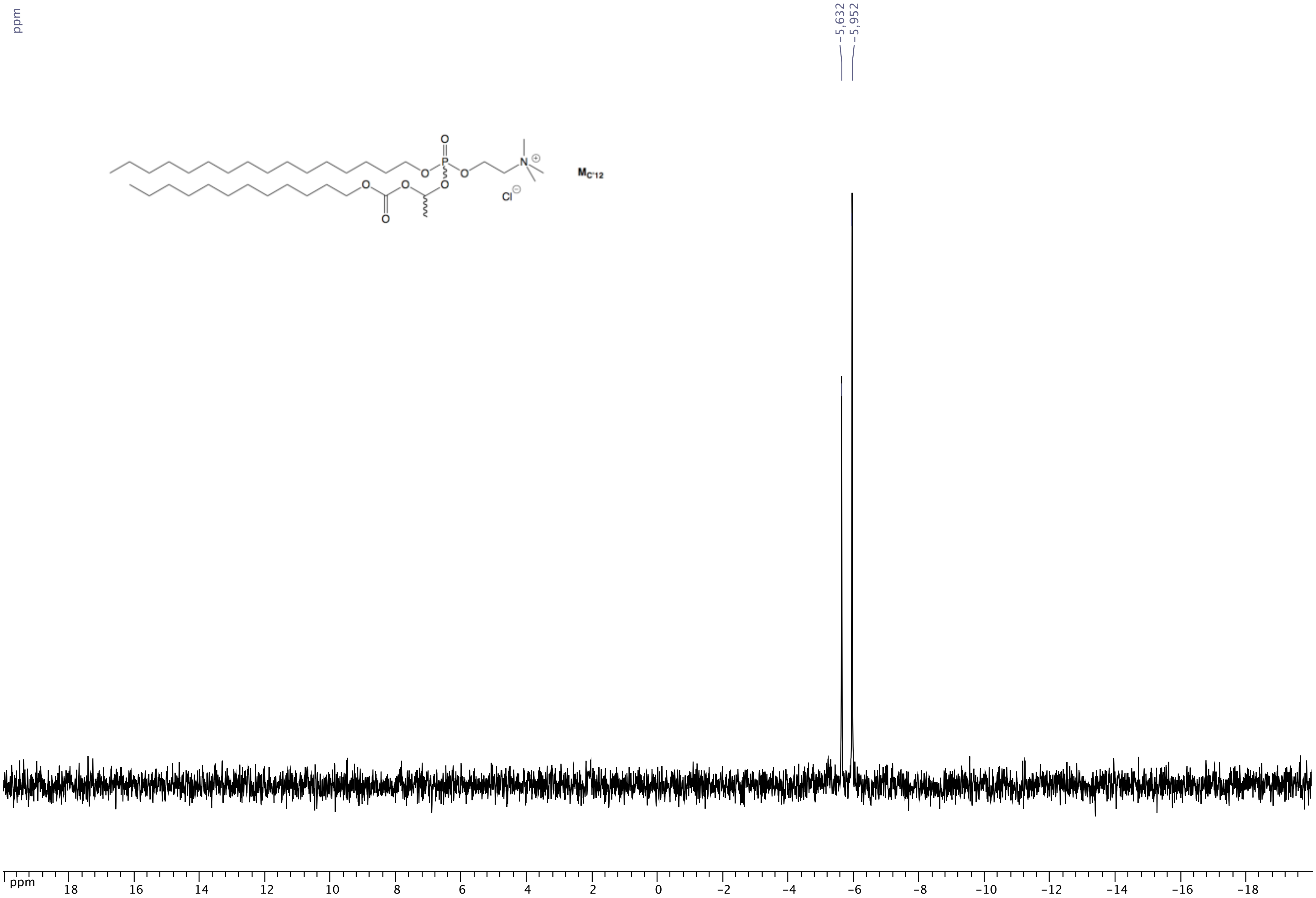
ppm
−5,632
−5,952
M$_{C'12}$
Cl
ppm
18
16
14
12
10
8
6
4
2
0
−2
−4
−6
−8
−10
−12
−14
−16
−18

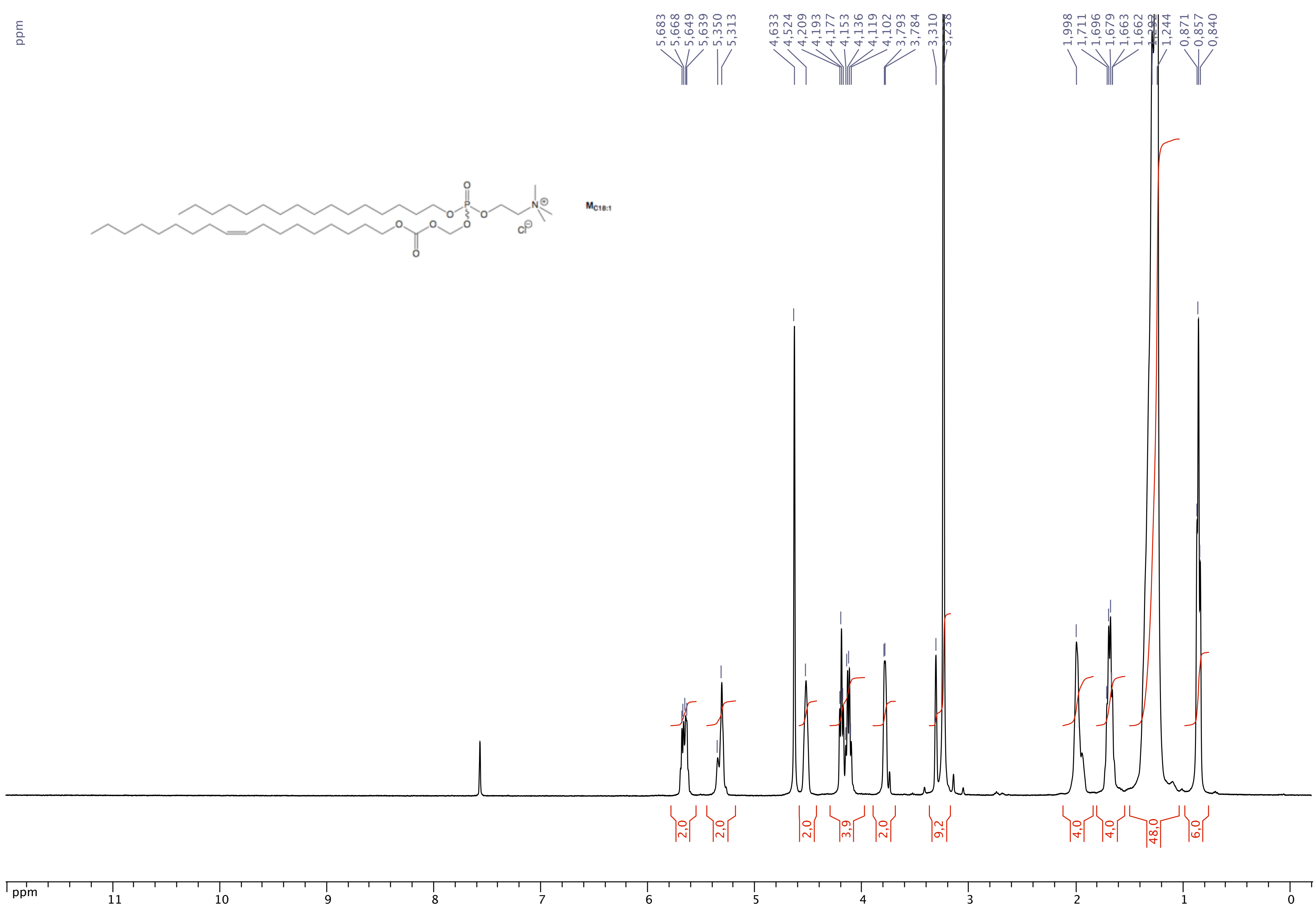
ppm
5,683
5,668
5,649
5,639
5,350
5,313
4,633
4,524
4,209
4,193
4,177
4,153
4,136
4,119
4,102
3,793
3,784
3,310
3,238
1,998
1,711
1,696
1,679
1,663
1,662
1,244
0,871
0,857
0,840
M C18:1
2,0
2,0
2,0
3,9
2,0
9,2
4,0
4,0
48,0
6,0
ppm
11
10
9
8
7
6
5
4
3
2
1
0

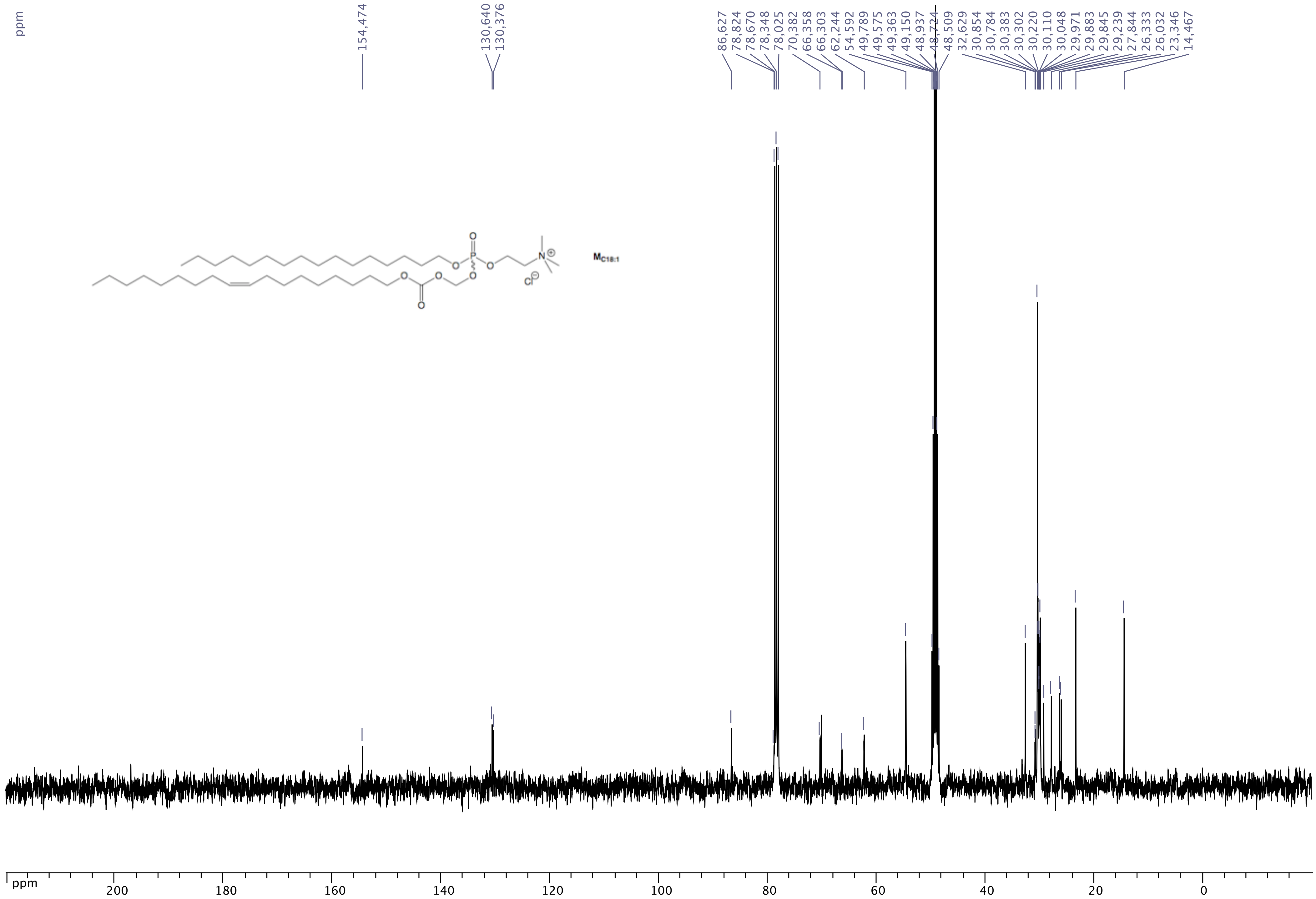
ppm
154,474
130,640
130,376
86,627
78,824
78,670
78,348
78,025
70,382
66,358
66,303
62,244
54,592
49,789
49,575
49,363
49,150
48,937
48,724
48,509
32,629
30,854
30,784
30,383
30,302
30,220
30,110
30,048
29,971
29,883
29,845
29,239
27,844
26,333
26,032
23,346
14,467
$M_{C18:1}$
ppm
200
180
160
140
120
100
80
60
40
20
0

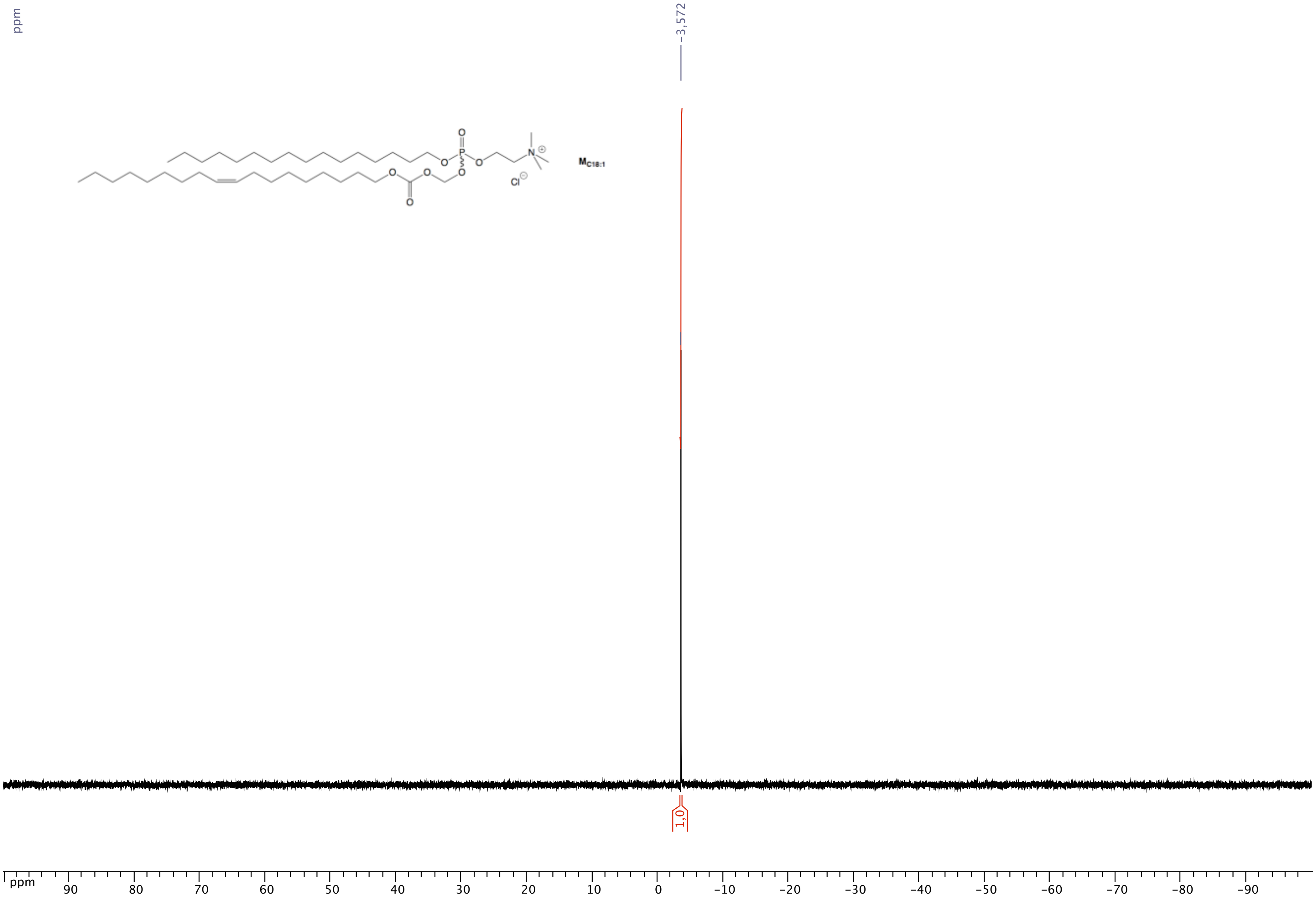
ppm
-3,572
M C18:1
1,0
ppm 90 80 70 60 50 40 30 20 10 0 -10 -20 -30 -40 -50 -60 -70 -80 -90

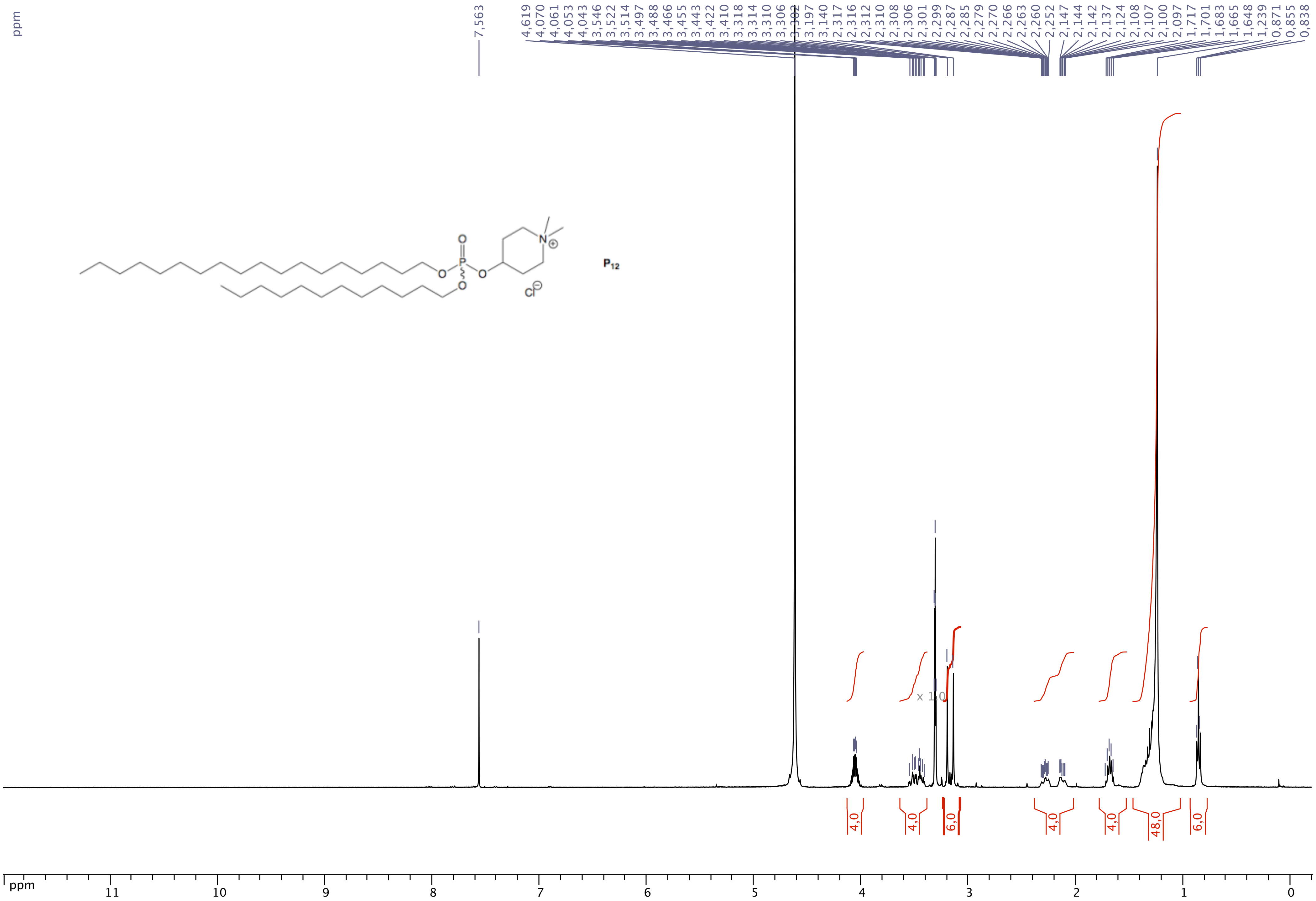
ppm
7,563
4,619
4,070
4,061
4,053
4,043
3,546
3,522
3,514
3,497
3,488
3,466
3,455
3,443
3,422
3,410
3,318
3,314
3,310
3,306
3,302
3,197
3,140
2,317
2,316
2,312
2,310
2,308
2,306
2,301
2,299
2,287
2,285
2,279
2,270
2,266
2,263
2,260
2,252
2,147
2,144
2,142
2,137
2,124
2,108
2,107
2,100
2,097
1,717
1,701
1,683
1,665
1,648
1,239
0,871
0,855
0,838
P12
4,0
4,0
6,0
4,0
4,0
48,0
6,0
ppm
11
10
9
8
7
6
5
4
3
2
1
0

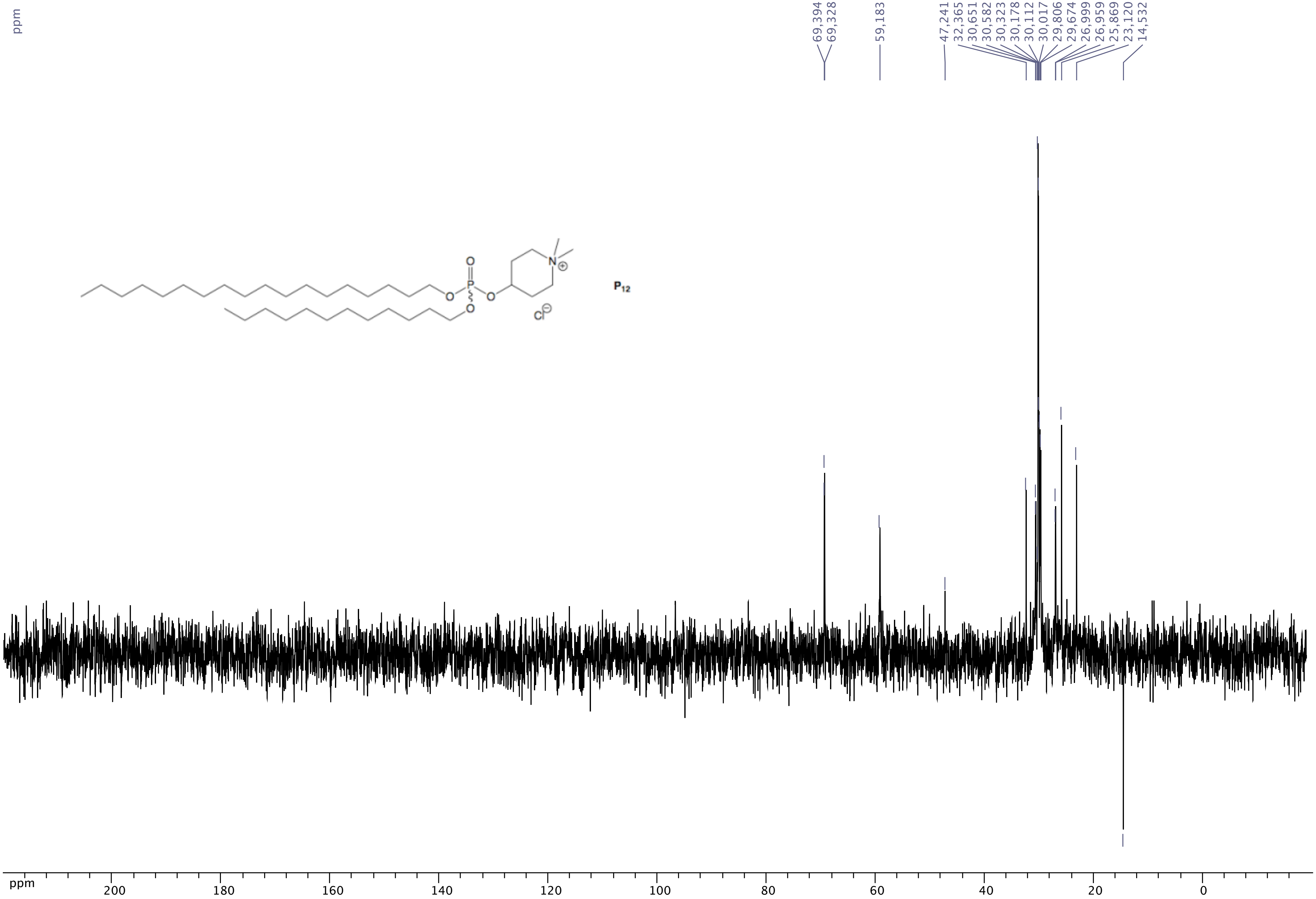
ppm
69,394
69,328
59,183
47,241
32,365
30,651
30,582
30,323
30,178
30,112
30,017
29,806
29,674
26,999
26,959
25,869
23,120
14,532
P12
ppm
200
180
160
140
120
100
80
60
40
20
0

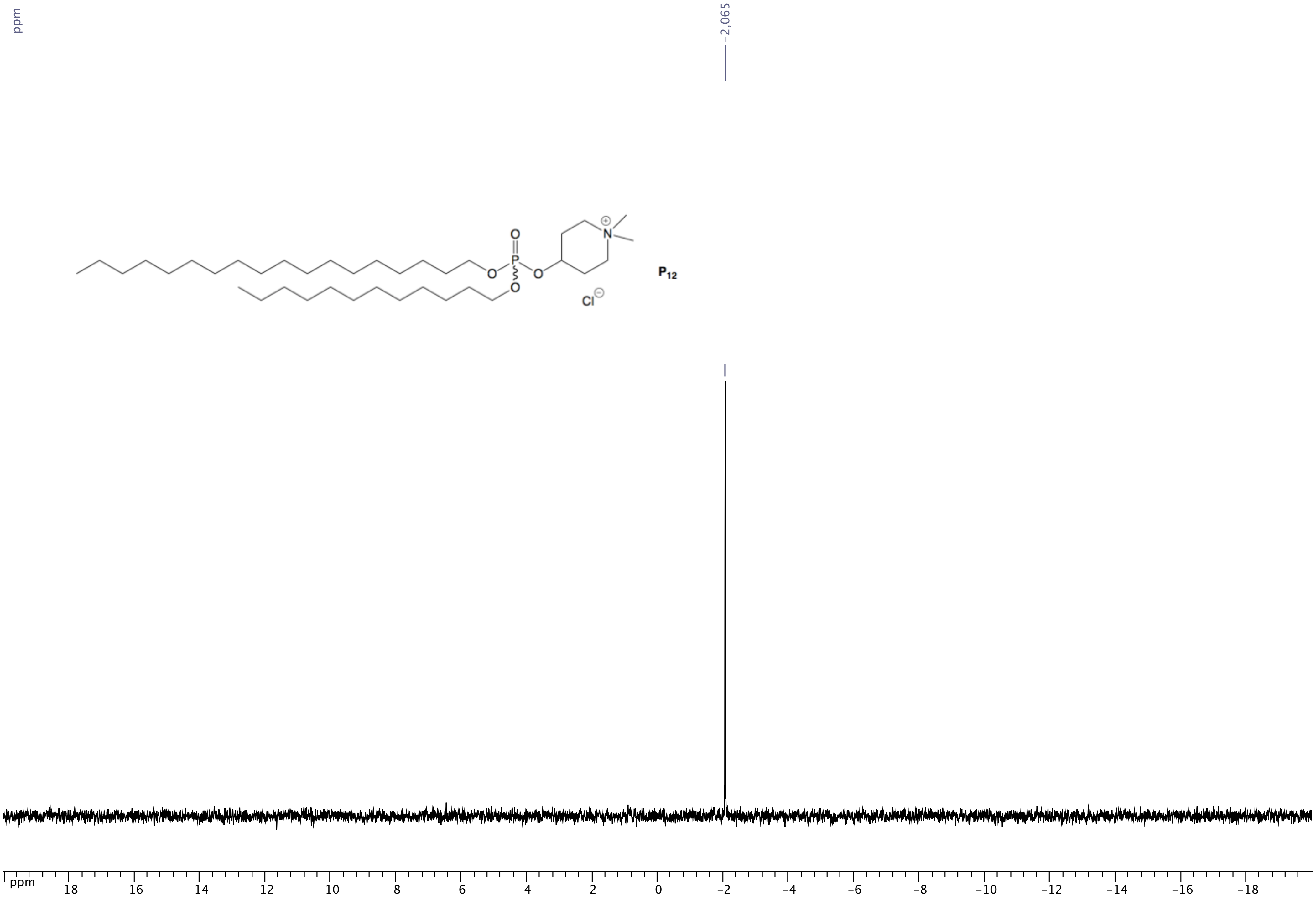
ppm
-2,065
P12
O
P
N
Cl
ppm
18
16
14
12
10
8
6
4
2
0
−2
−4
−6
−8
−10
−12
−14
−16
−18

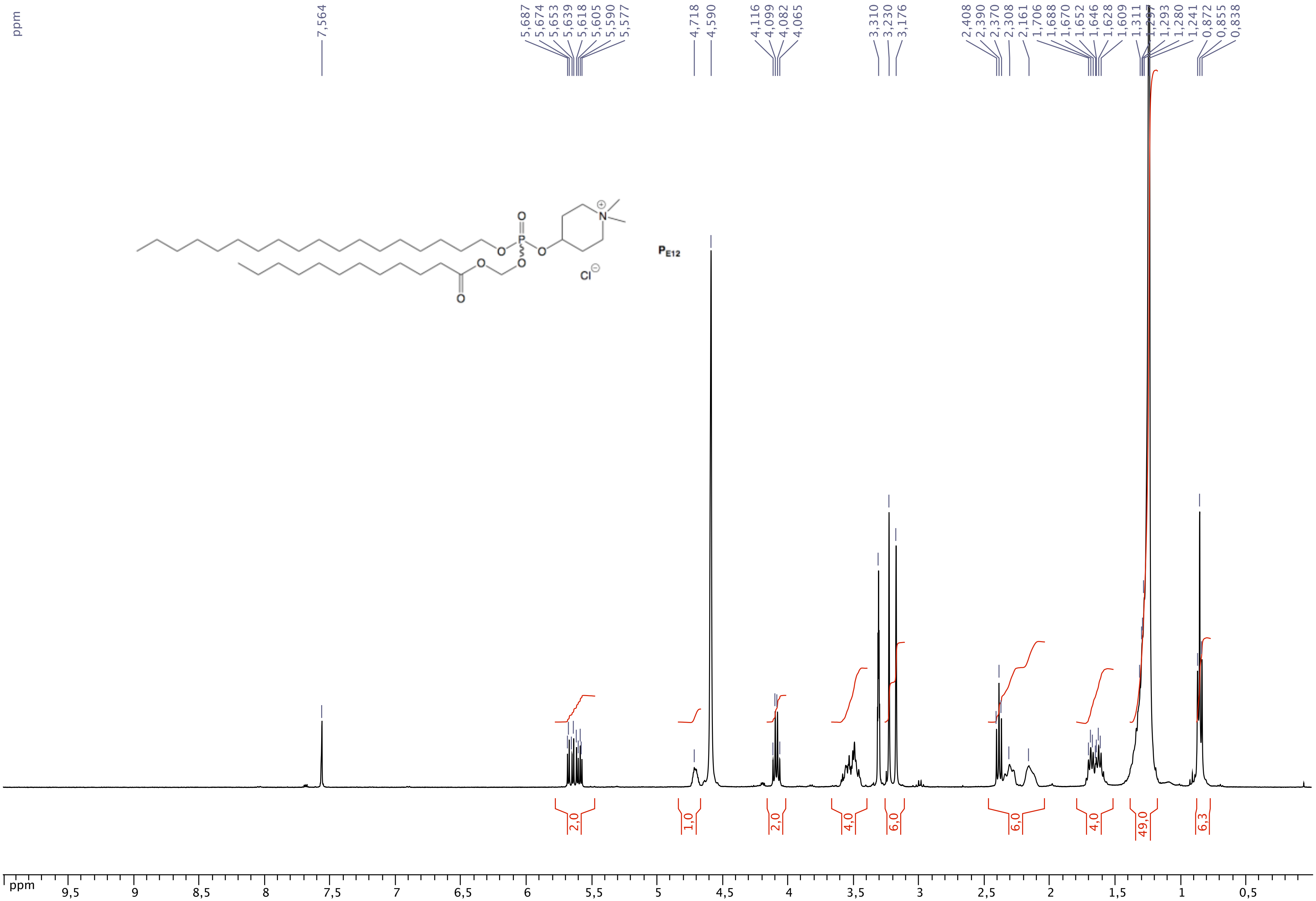
ppm
7,564
5,687
5,674
5,653
5,639
5,618
5,605
5,590
5,577
4,718
4,590
4,116
4,099
4,082
4,065
3,310
3,230
3,176
2,408
2,390
2,370
2,308
2,161
1,706
1,688
1,670
1,652
1,646
1,628
1,609
1,311
1,293
1,280
1,241
0,872
0,855
0,838
P_{E12}
O
O
O
O
P
N
Cl
2,0
1,0
2,0
4,0
6,0
6,0
4,0
49,0
6,3
ppm
9,5
9
8,5
8
7,5
7
6,5
6
5,5
5
4,5
4
3,5
3
2,5
2
1,5
1
0,5

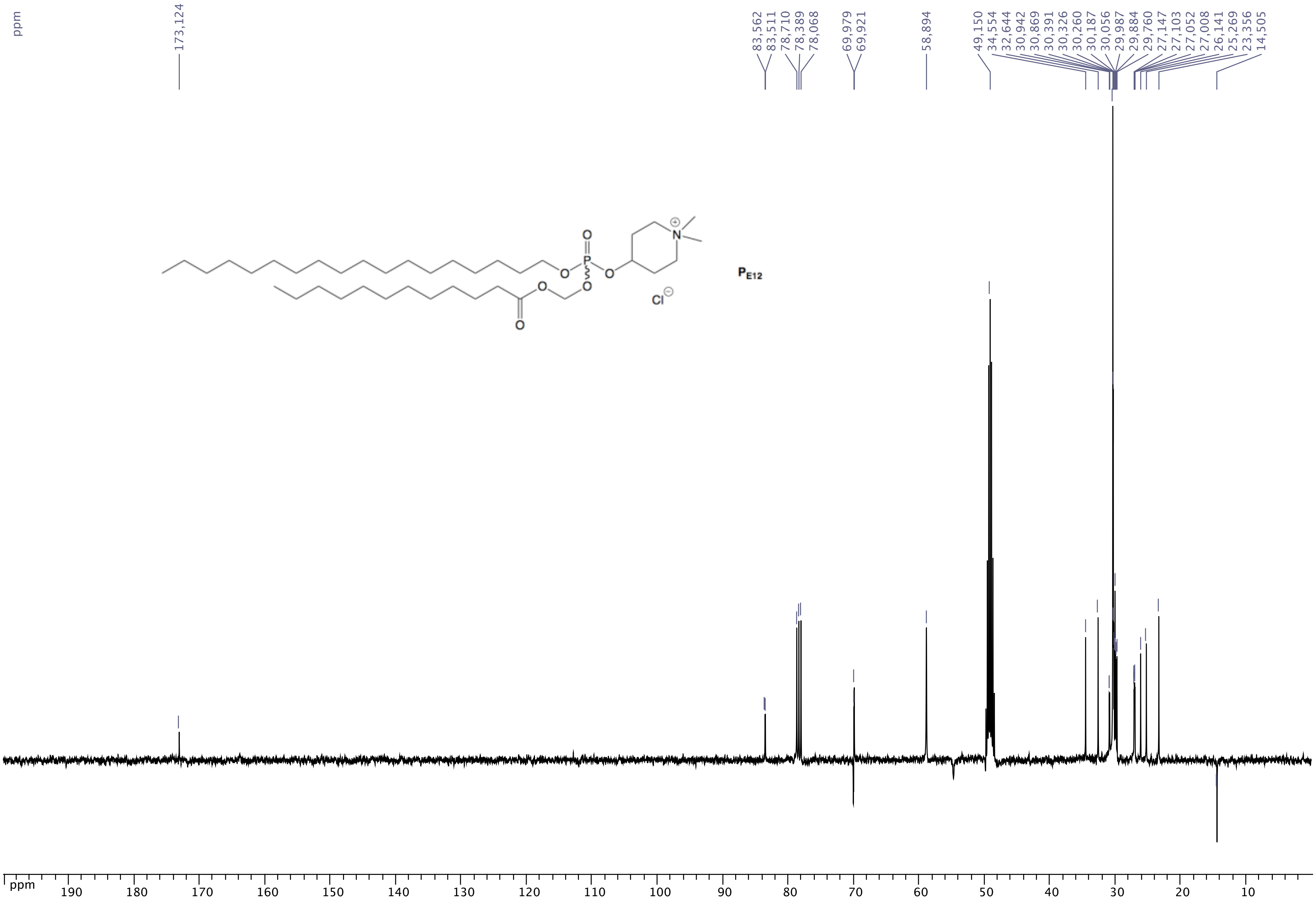
ppm
173,124
83,562
83,511
78,710
78,389
78,068
69,979
69,921
58,894
49,150
34,554
32,644
30,942
30,869
30,391
30,326
30,260
30,187
30,056
29,987
29,884
29,760
27,147
27,103
27,052
27,008
26,141
25,269
23,356
14,505
P_{E12}
ppm
190
180
170
160
150
140
130
120
110
100
90
80
70
60
50
40
30
20
10

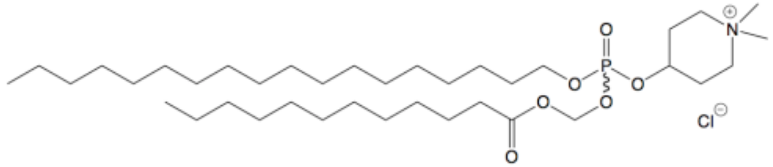

P_E12

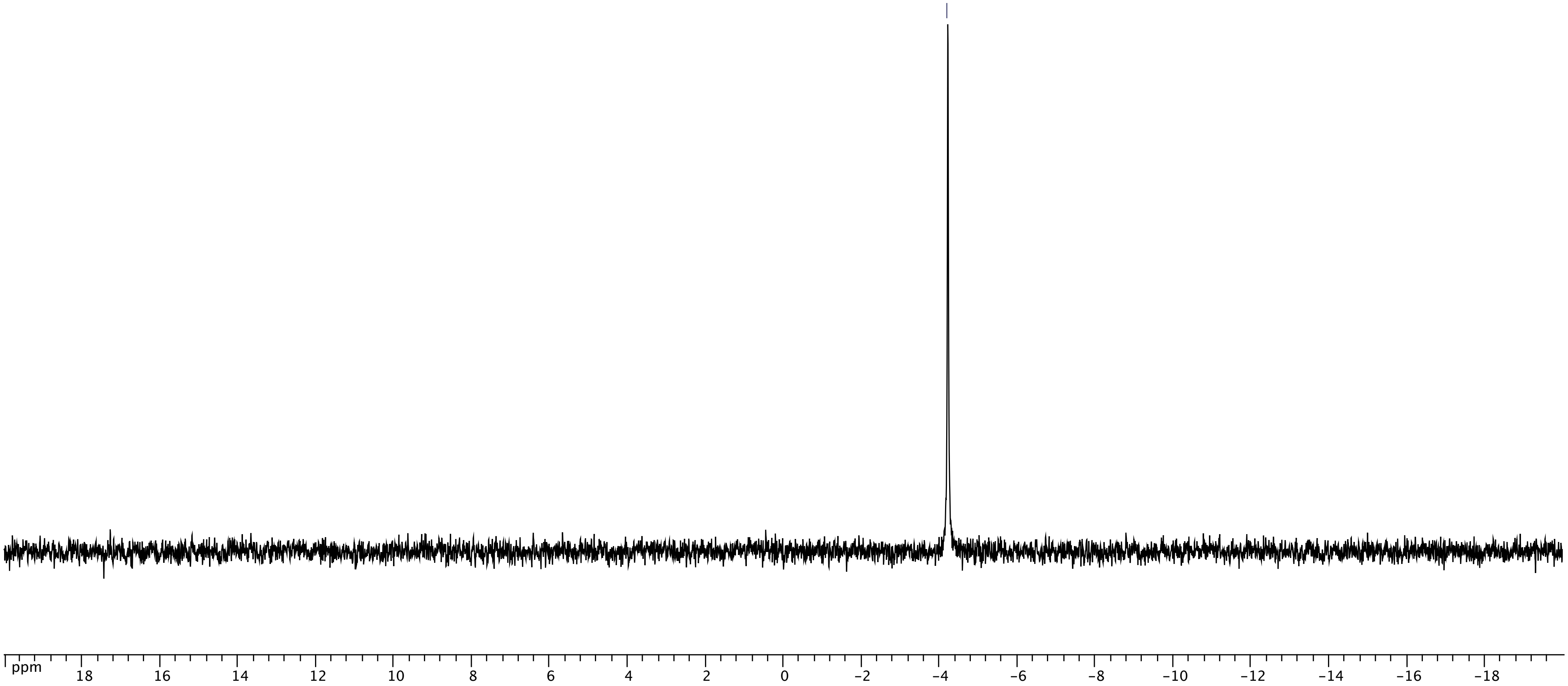

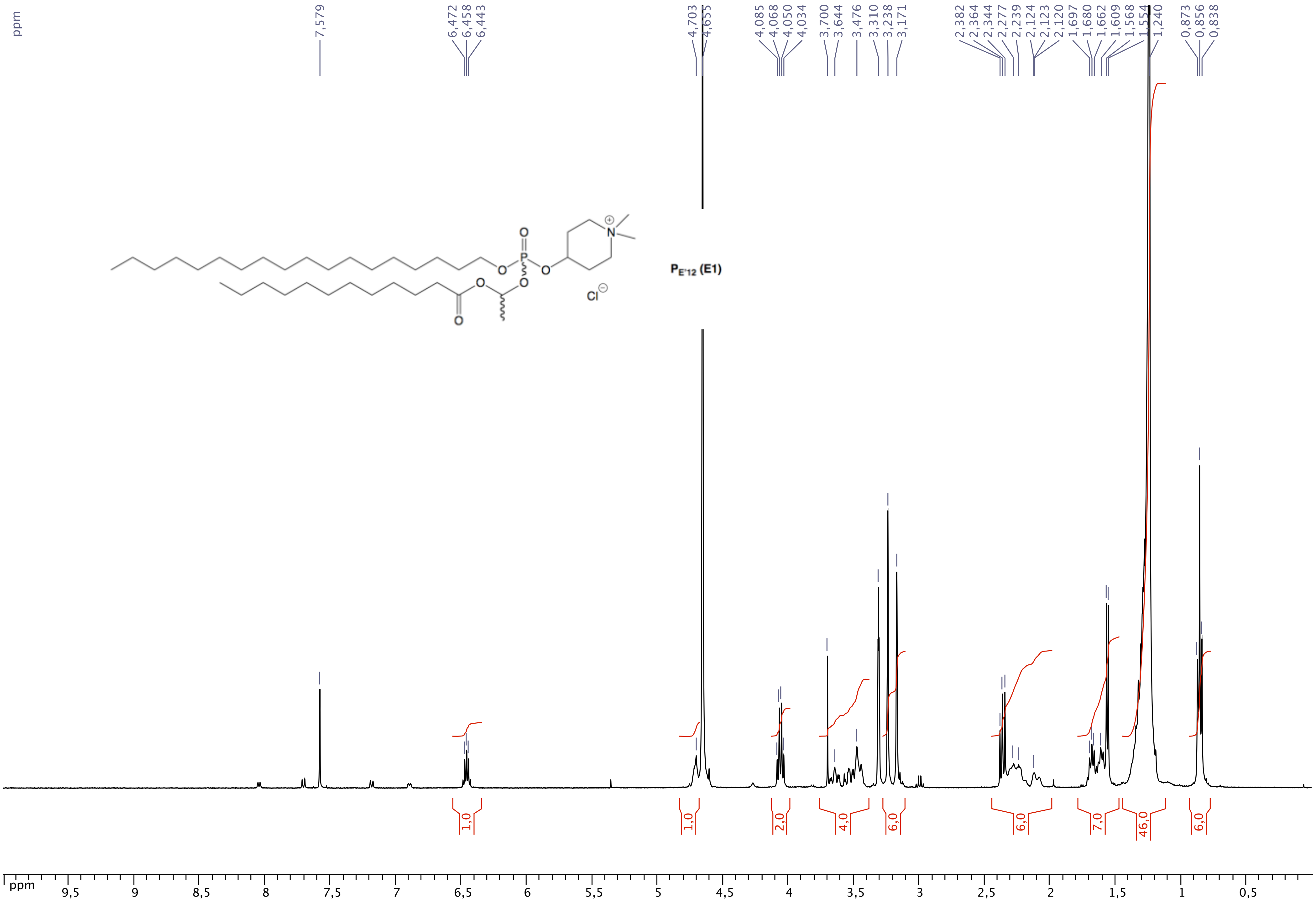
ppm
7,579
6,472
6,458
6,443
4,703
4,655
4,085
4,068
4,050
4,034
3,700
3,644
3,476
3,310
3,238
3,171
2,382
2,364
2,344
2,277
2,239
2,124
2,123
2,120
1,697
1,680
1,662
1,609
1,568
1,554
1,240
0,873
0,856
0,838
O
P
O
O
O
O
O
N
Cl
PE'12 (E1)
1,0
1,0
2,0
4,0
6,0
6,0
7,0
46,0
6,0
ppm
9,5
9
8,5
8
7,5
7
6,5
6
5,5
5
4,5
4
3,5
3
2,5
2
1,5
1
0,5

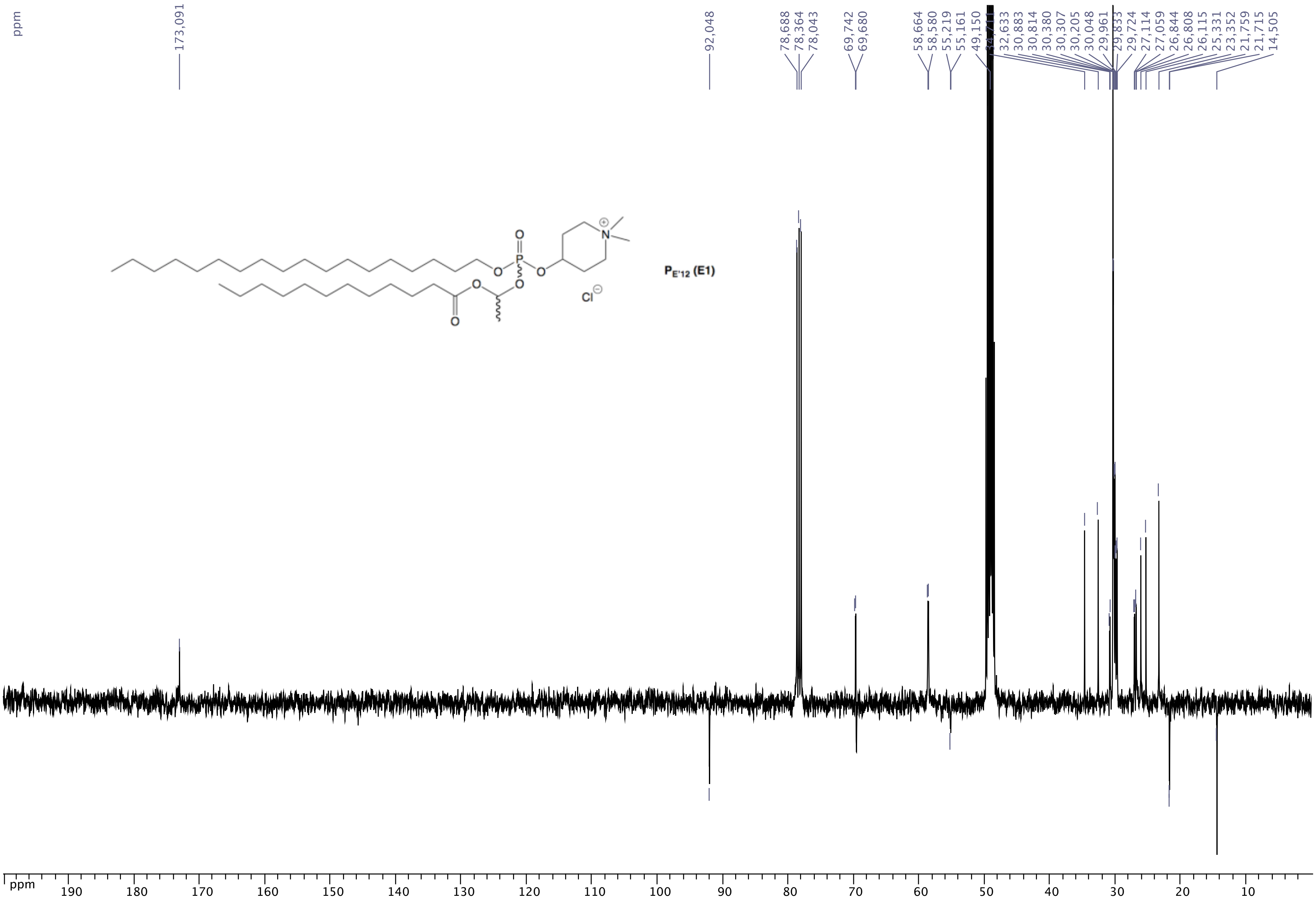
ppm
173,091
92,048
78,688
78,364
78,043
69,742
69,680
58,664
58,580
55,219
55,161
49,150
32,633
30,883
30,814
30,380
30,307
30,205
30,048
29,961
29,833
29,724
27,114
27,059
26,844
26,808
26,115
25,331
23,352
21,759
21,715
14,505
$P_{E'12}$ (E1)
ppm
190
180
170
160
150
140
130
120
110
100
90
80
70
60
50
40
30
20
10

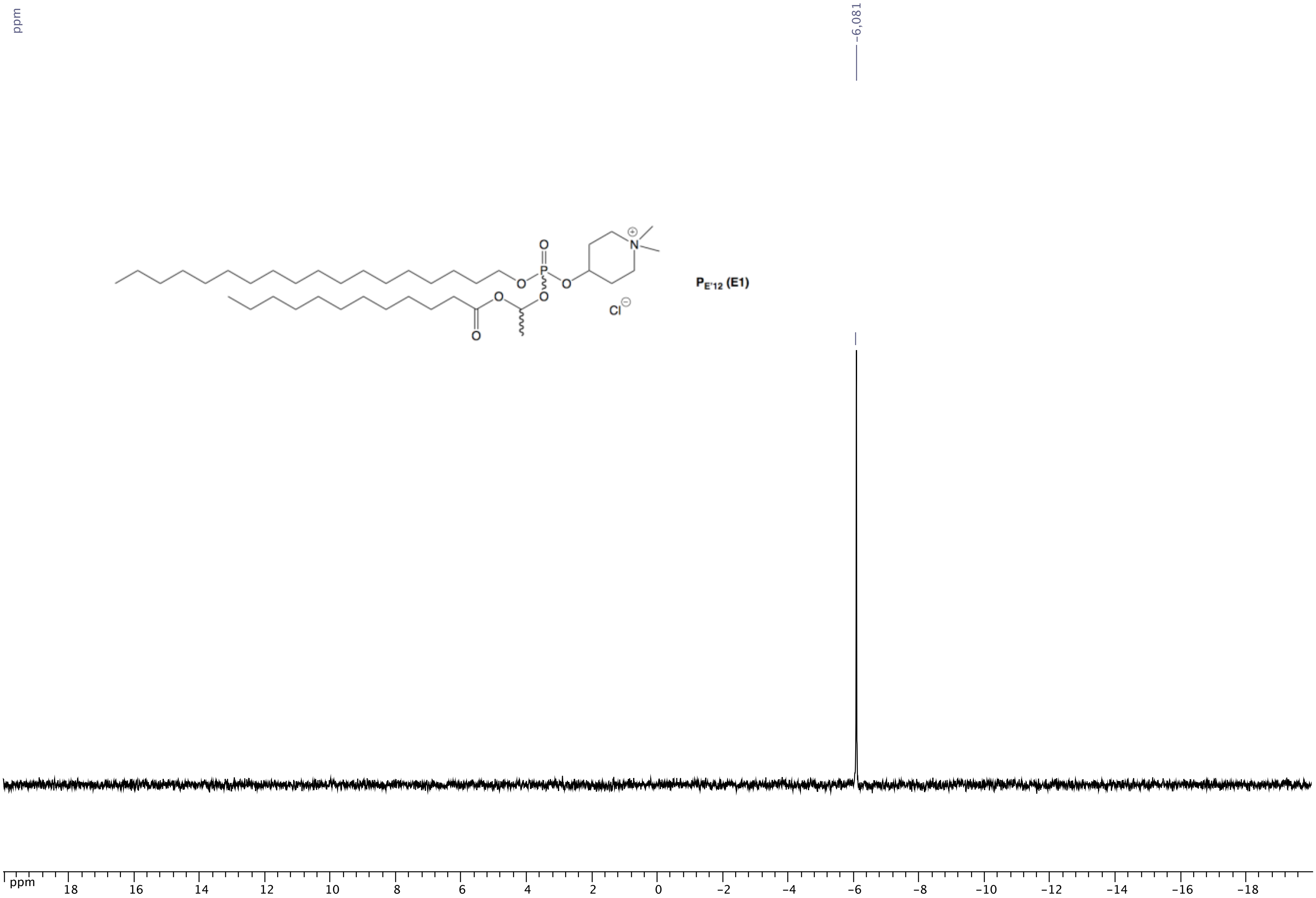

$P_{E'12}$ (E1)

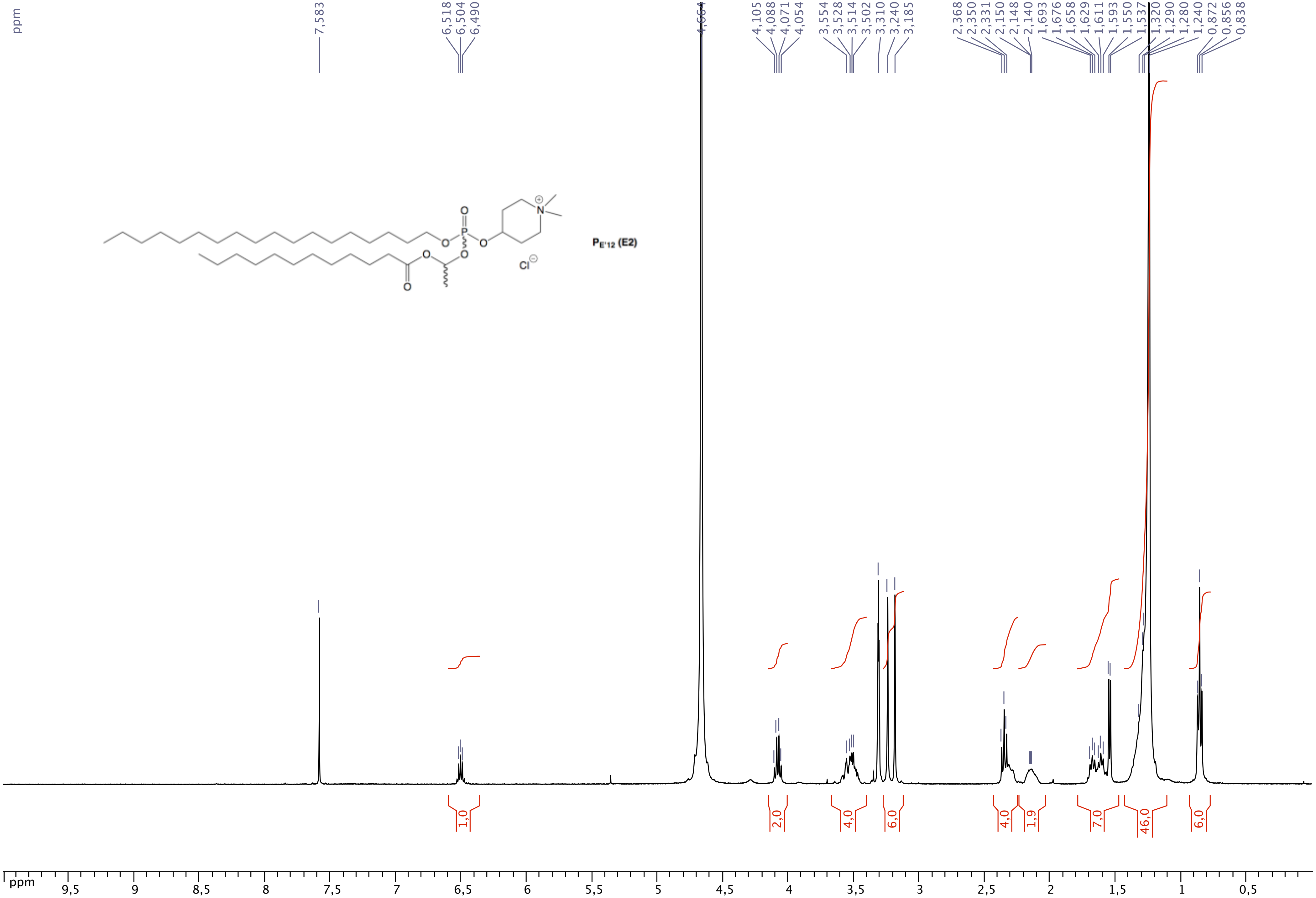
ppm
7,583
6,518
6,504
6,490
4,664
4,105
4,088
4,071
4,054
3,554
3,528
3,514
3,502
3,310
3,240
3,185
2,368
2,350
2,331
2,150
2,148
2,140
1,693
1,676
1,658
1,629
1,611
1,593
1,550
1,537
1,320
1,290
1,280
1,240
0,872
0,856
0,838
$P_{E'12}$ (E2)
Cl
1,0
2,0
4,0
6,0
4,0
1,9
7,0
46,0
6,0
ppm
9,5
9
8,5
8
7,5
7
6,5
6
5,5
5
4,5
4
3,5
3
2,5
2
1,5
1
0,5

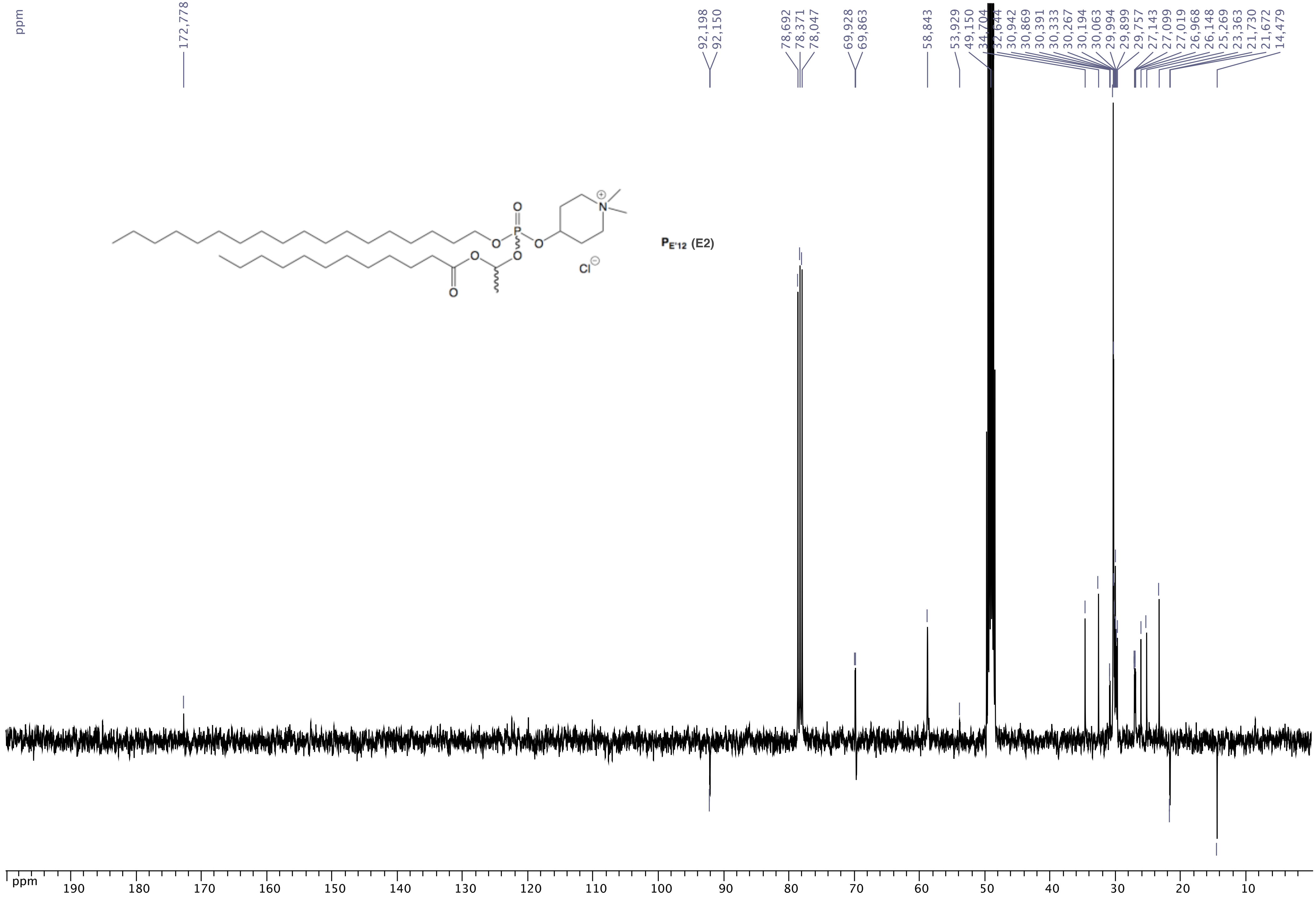
ppm
172,778
92,198
92,150
78,692
78,371
78,047
69,928
69,863
58,843
53,929
49,150
34,704
32,644
30,942
30,869
30,391
30,333
30,267
30,194
30,063
29,994
29,899
29,757
27,143
27,099
27,019
26,968
26,148
25,269
23,363
21,730
21,672
14,479
P_E'12 (E2)
Cl
ppm
190
180
170
160
150
140
130
120
110
100
90
80
70
60
50
40
30
20
10

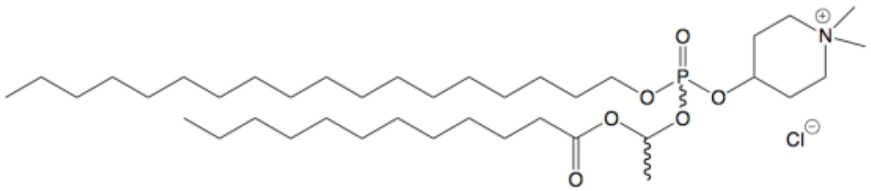

$P_{E'12}$ (E2)

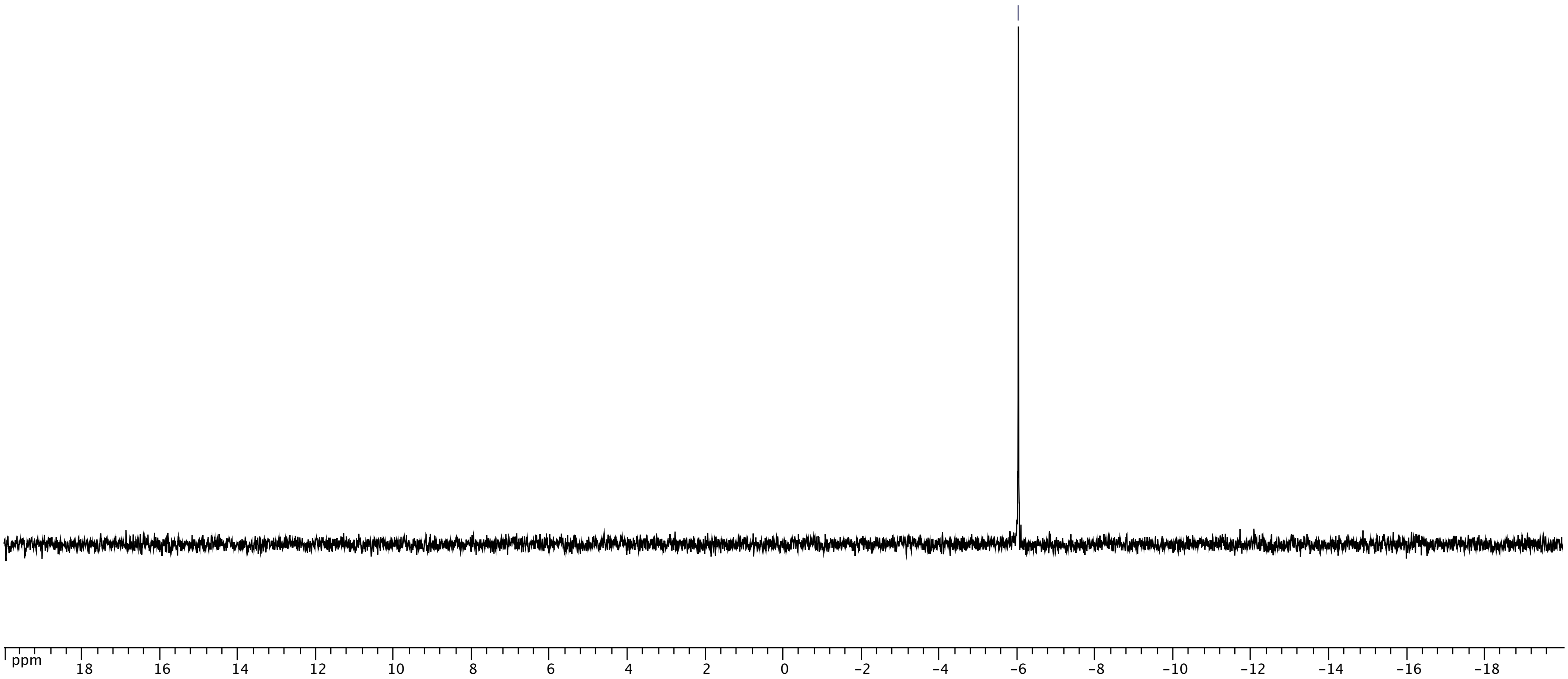

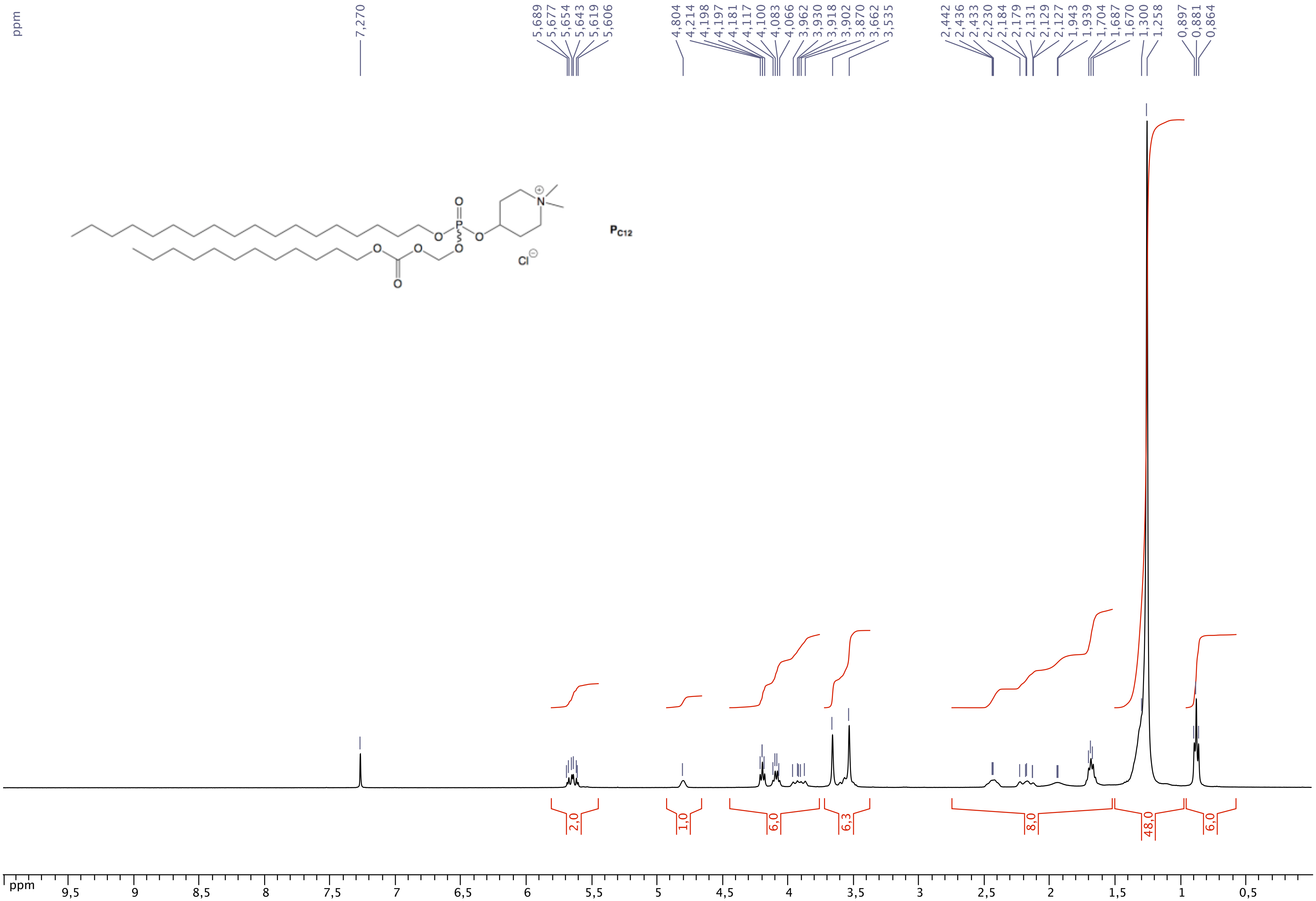

ppm
7,270
5,689
5,677
5,654
5,643
5,619
5,606
4,804
4,214
4,198
4,197
4,181
4,117
4,100
4,083
4,066
3,962
3,930
3,918
3,902
3,870
3,662
3,535
2,442
2,436
2,433
2,230
2,184
2,179
2,131
2,129
2,127
1,943
1,939
1,704
1,687
1,670
1,300
1,258
0,897
0,881
0,864
P_{C12}
2,0
1,0
6,0
6,3
8,0
48,0
6,0
ppm 9,5 9 8,5 8 7,5 7 6,5 6 5,5 5 4,5 4 3,5 3 2,5 2 1,5 1 0,5

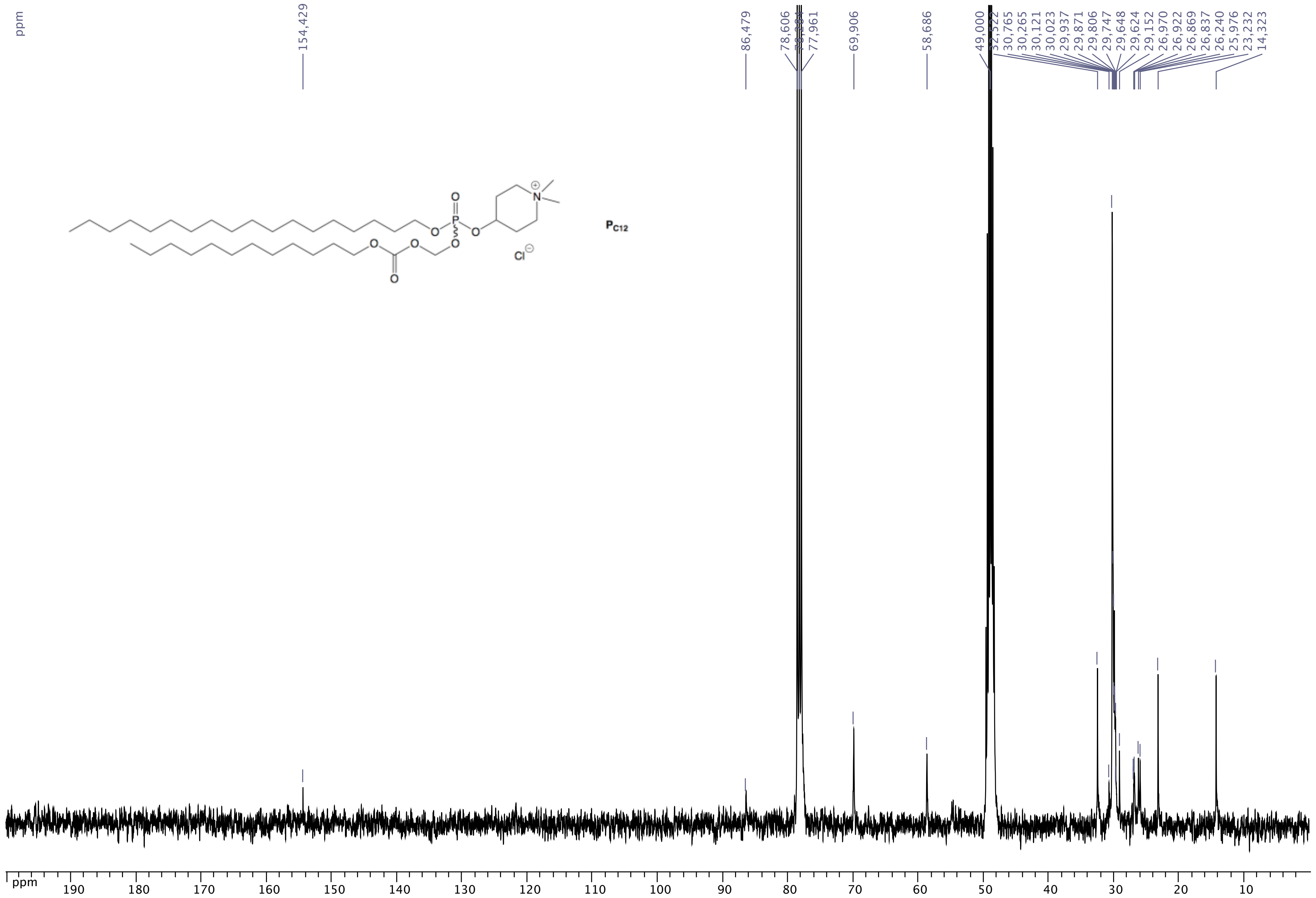
ppm
154,429
86,479
78,606
77,961
69,906
58,686
49,000
32,522
30,765
30,265
30,121
30,023
29,937
29,871
29,806
29,747
29,648
29,624
29,152
26,970
26,922
26,869
26,837
26,240
25,976
23,232
14,323
P_{C12}
ppm
190
180
170
160
150
140
130
120
110
100
90
80
70
60
50
40
30
20
10

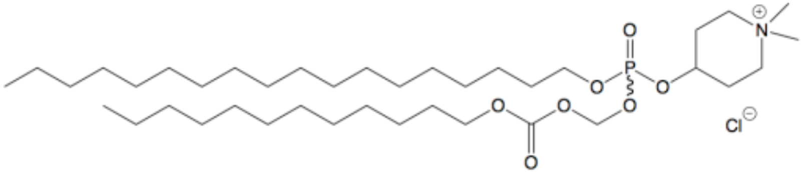
O
P
O
O
O
O
O
O
N
Cl

P$_{C12}$

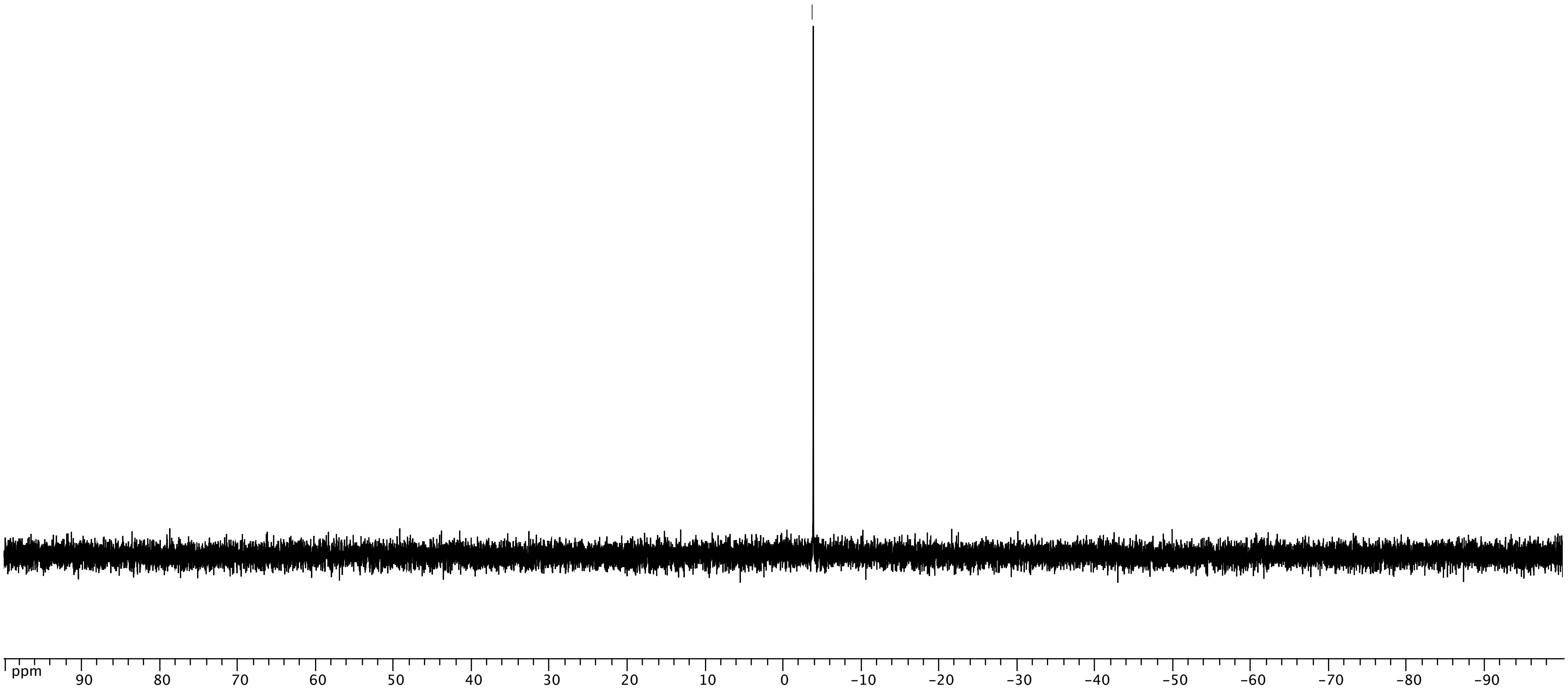
ppm
−3,820
ppm
90
80
70
60
50
40
30
20
10
0
−10
−20
−30
−40
−50
−60
−70
−80
−90

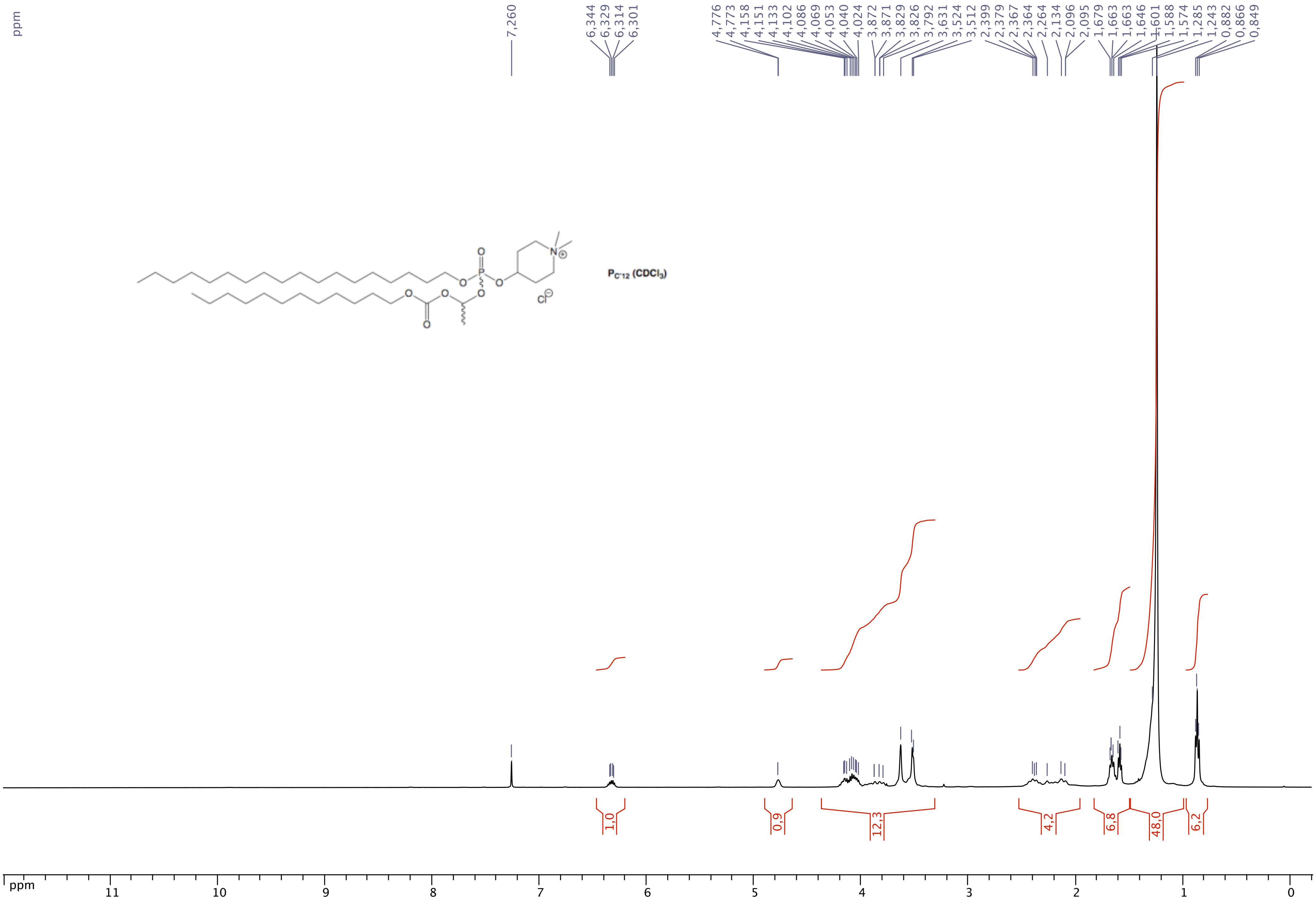

ppm
7,260
6,344
6,329
6,314
6,301
4,776
4,773
4,158
4,151
4,133
4,102
4,086
4,069
4,053
4,040
4,024
3,872
3,871
3,829
3,826
3,792
3,631
3,524
3,512
2,399
2,379
2,367
2,364
2,264
2,134
2,096
2,095
1,679
1,663
1,663
1,646
1,601
1,588
1,574
1,285
1,243
0,882
0,866
0,849
P C'12 (CDCl3)
1,0
0,9
12,3
4,2
6,8
48,0
6,2
ppm
11
10
9
8
7
6
5
4
3
2
1
0

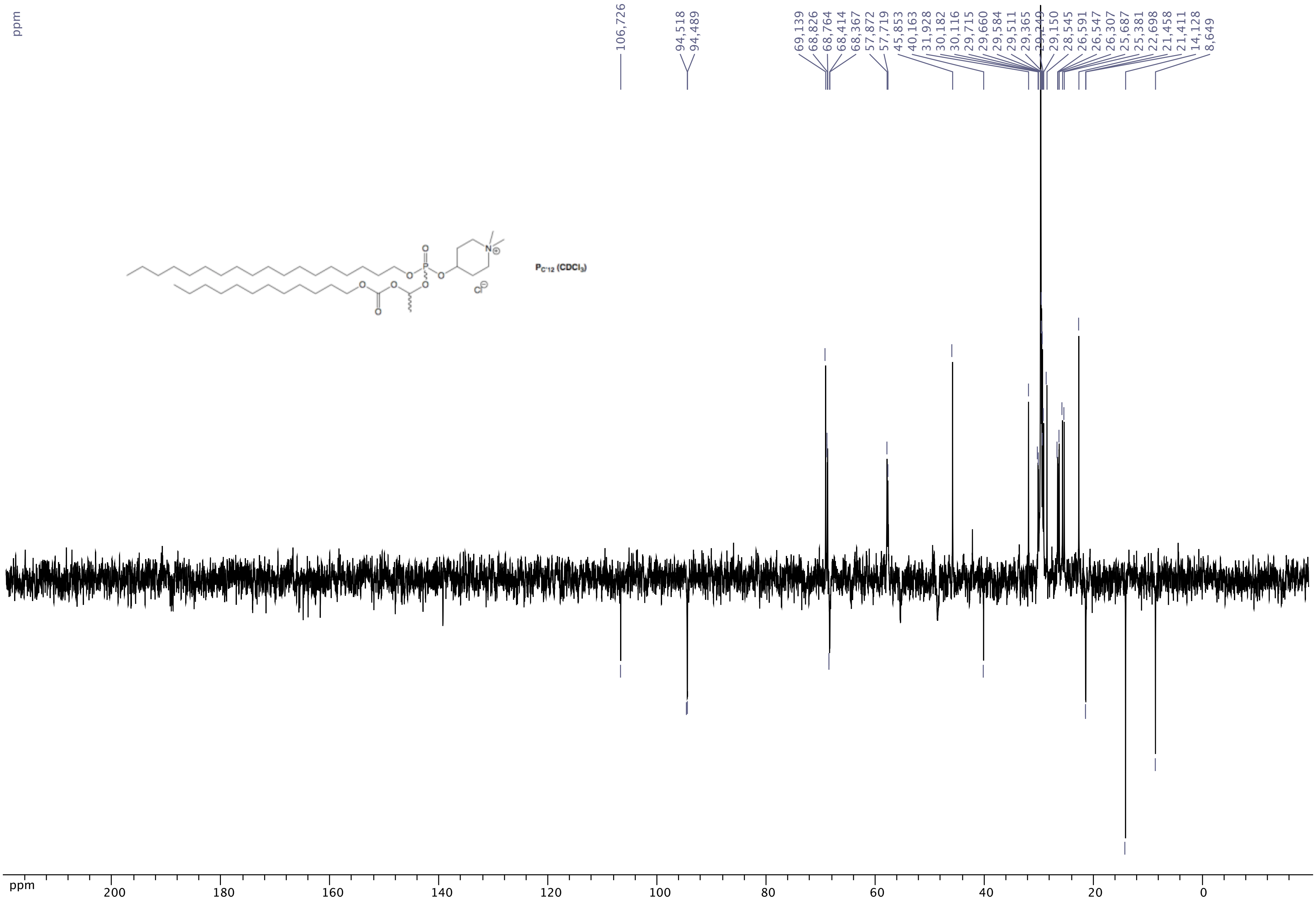

ppm
106,726
94,518
94,489
69,139
68,826
68,764
68,414
68,367
57,872
57,719
45,853
40,163
31,928
30,182
30,116
29,715
29,660
29,584
29,511
29,365
29,249
29,150
28,545
26,591
26,547
26,307
25,687
25,381
22,698
21,458
21,411
14,128
8,649
$P_{C'12}$ ($CDCl_3$)
ppm
200
180
160
140
120
100
80
60
40
20
0

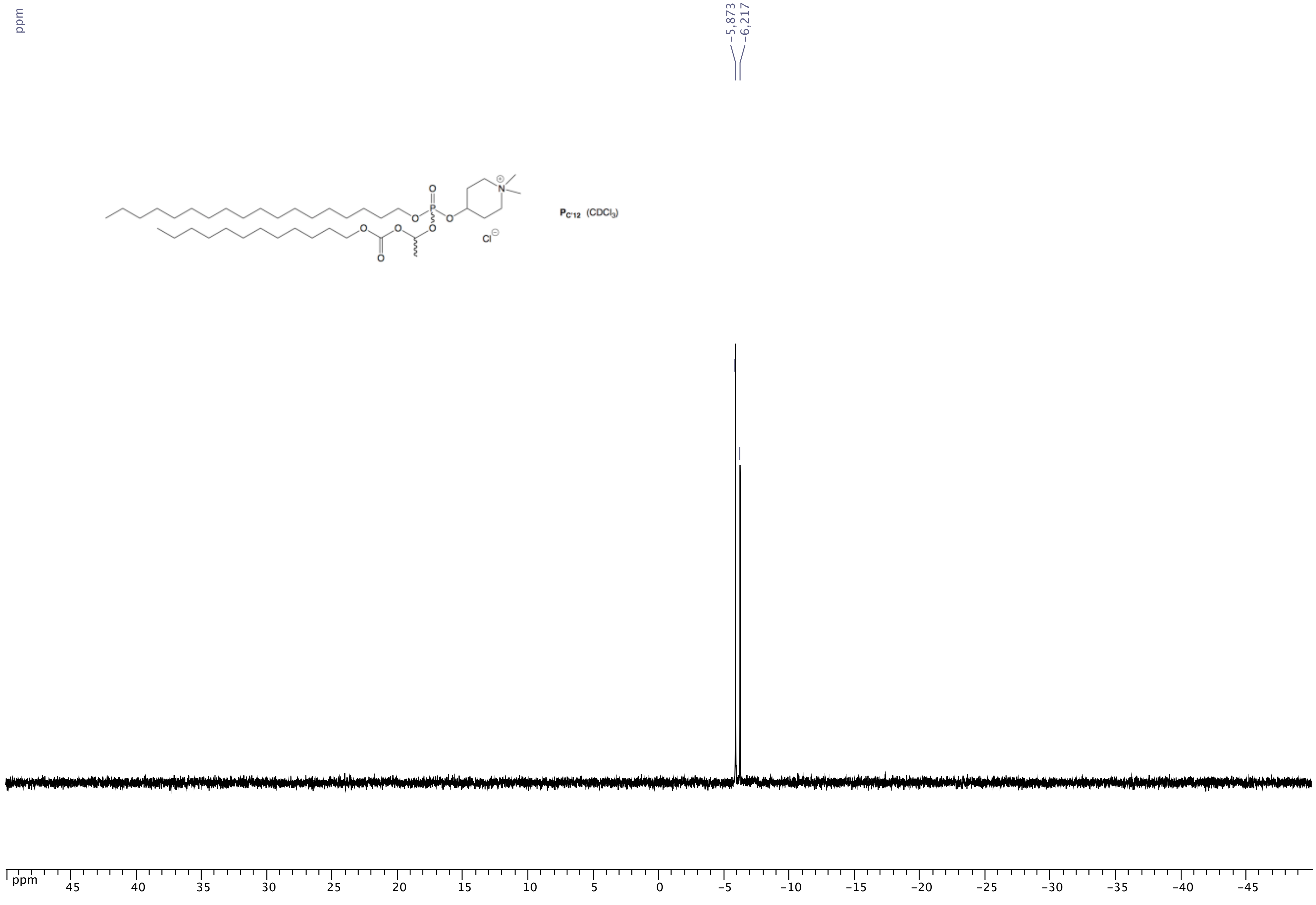

ppm
−5,873
−6,217
P_C'12 (CDCl3)
Cl
ppm
45 40 35 30 25 20 15 10 5 0 −5 −10 −15 −20 −25 −30 −35 −40 −45

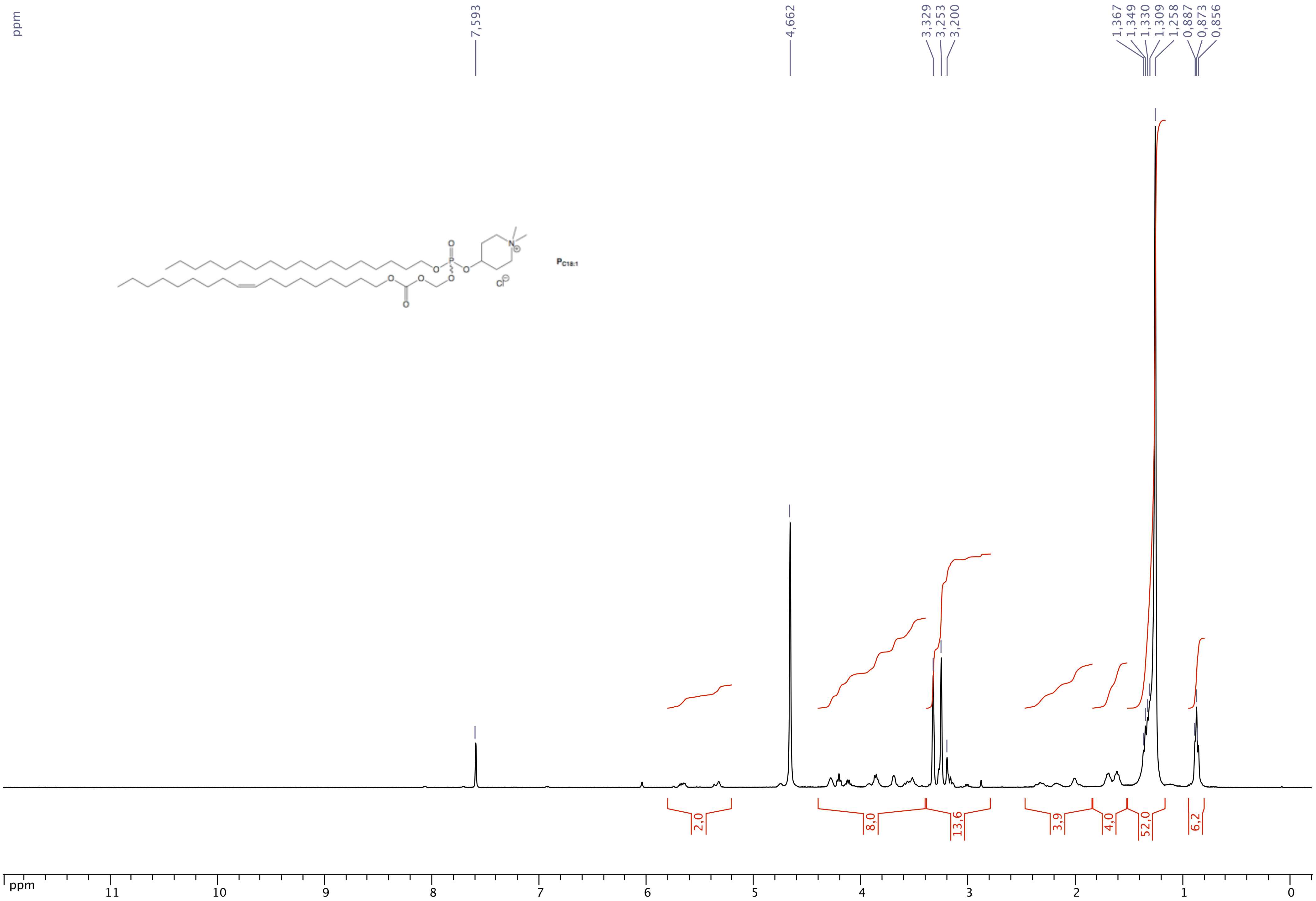

ppm
7,593
4,662
3,329
3,253
3,200
1,367
1,349
1,330
1,309
1,258
0,887
0,873
0,856
P C18:1
2,0
8,0
13,6
3,9
4,0
52,0
6,2
ppm
11
10
9
8
7
6
5
4
3
2
1
0

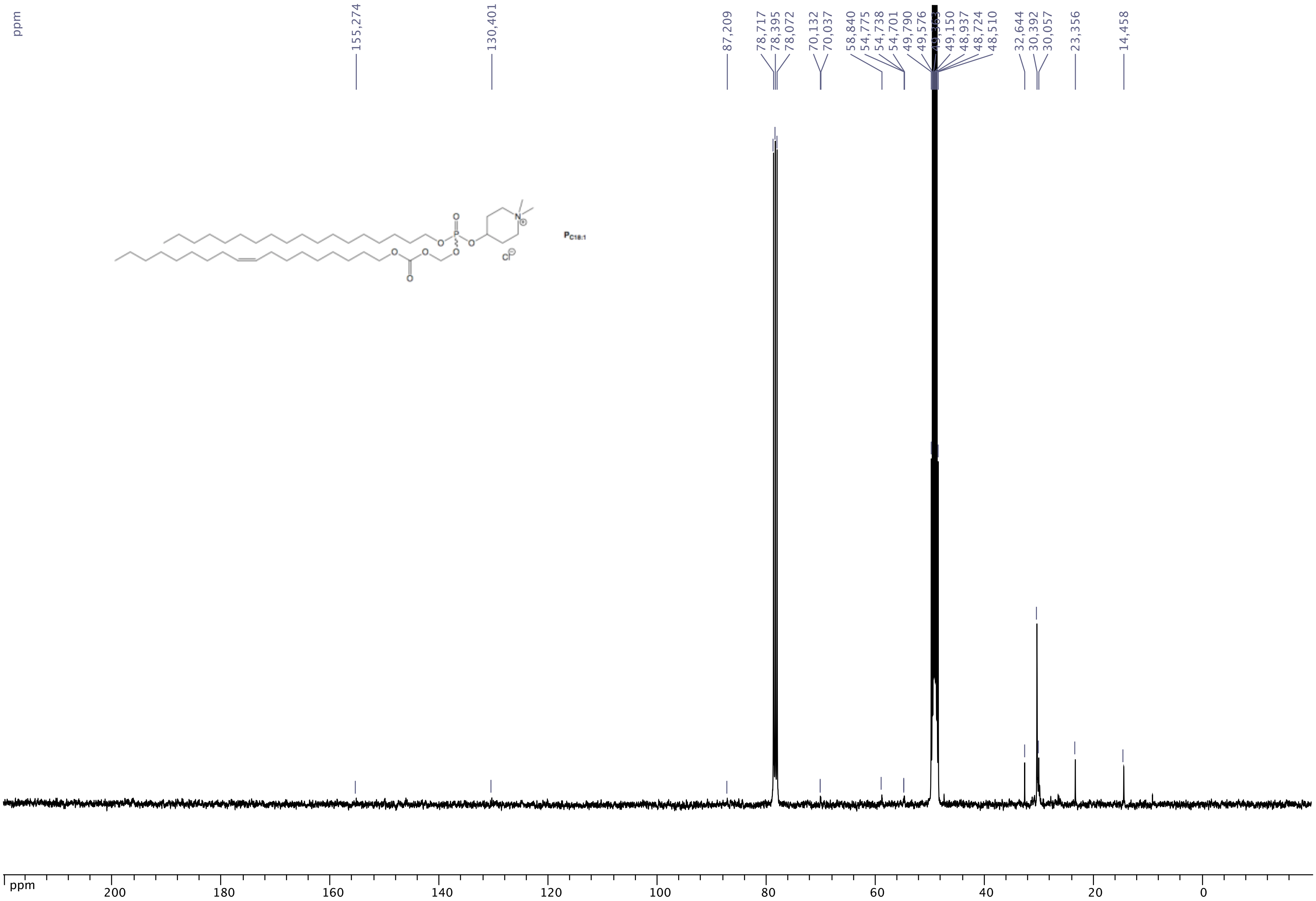

ppm
155,274
130,401
87,209
78,717
78,395
78,072
70,132
70,037
58,840
54,775
54,738
54,701
49,790
49,576
49,150
48,937
48,724
48,510
32,644
30,392
30,057
23,356
14,458
PC18:1
ppm
200
180
160
140
120
100
80
60
40
20
0

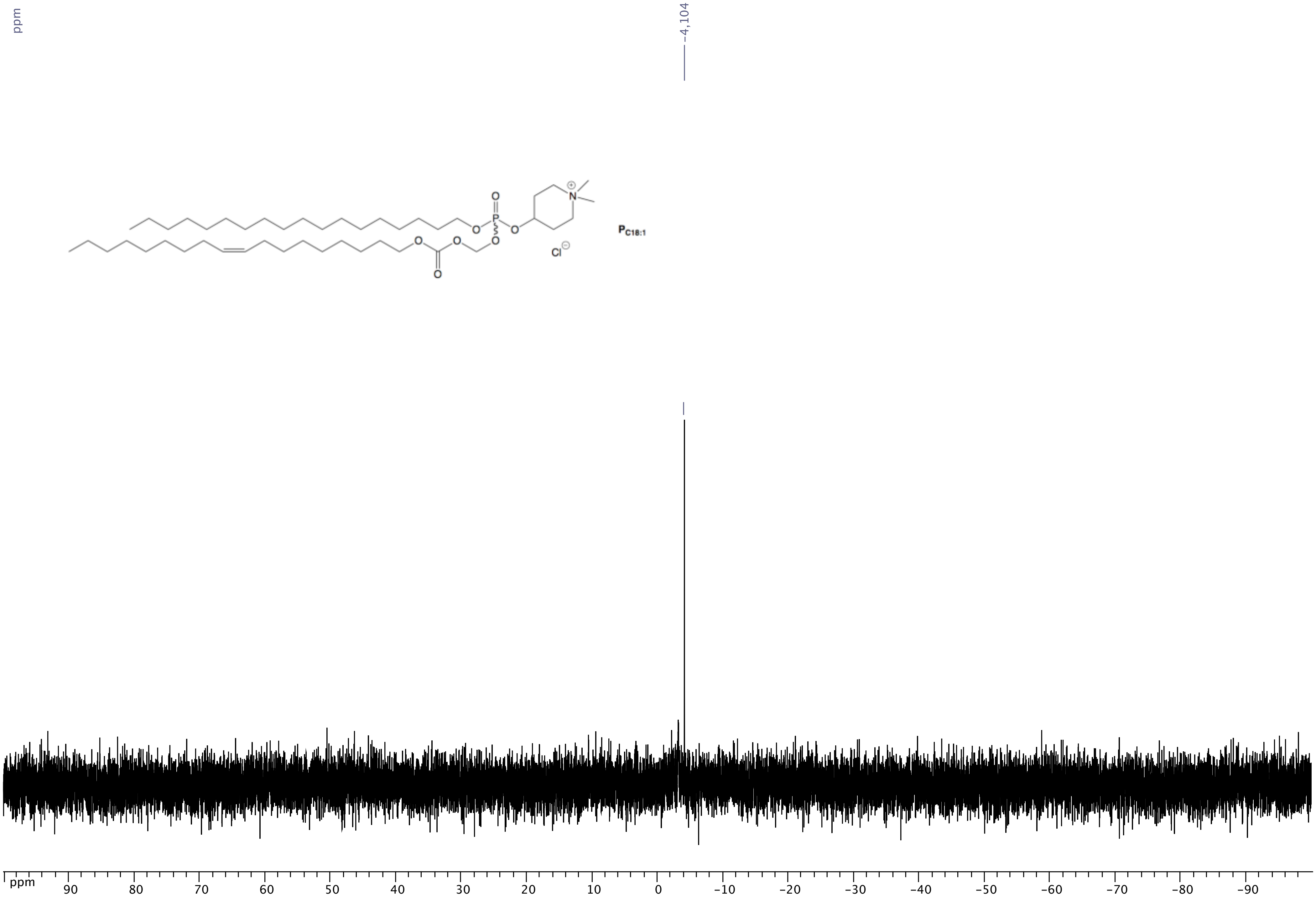
ppm
-4,104
P_C18:1
Cl
ppm
90
80
70
60
50
40
30
20
10
0
−10
−20
−30
−40
−50
−60
−70
−80
−90

References

Evans, B. C., Nelson, C. E., Yu, S. S., Beavers, K. R., Kim, A. J., Li, H., Nelson, H. M., Giorgio, T. D. & Duvall, C. L. (2013) Ex vivo red blood cell hemolysis assay for the evaluation of pH-responsive endosomolytic agents for cytosolic delivery of biomacromolecular drugs. *J. Vis. Exp.* 73, e50166. doi: 10.3791/50166

Gaillard, B., Remy, J.-S., Pons, F. & Lebeau, L. (2019) Erufosine (ErPC3) cationic prodrugs as dual gene delivery reagents for combined antitumor therapy. *Chem.-Eur. J.* 25, 15662–15679. doi: 10.1002/chem.201903976

Gentine, P., Bubel, A., Crucifix, C., Bourel-Bonnet, L. & Frisch, B. (2012) Manufacture of liposomes by isopropanol injection: characterization of the method. *J. Liposome Res.* 22, 18–30. doi: 10.3109/08982104.2011.584318

Goddard, E. D., Turro, N. J., Kuo, P. L. & Ananthapadmanabhan, K. P. (1985) Fluorescence probes for critical micelle concentration determination. *Langmuir* 1, 352–355. doi: 10.1021/la00063a015

Lebeau, L., Olland, S., Oudet, P. & Mioskowski, C. (1992) Rational design and synthesis of phospholipids for the two-dimensional crystallization of DNA gyrase, a key element in chromosome organization. *Chem. Phys. Lipids* 62, 93–103. doi: 10.1016/0009-3084(92)90087-6

Piñeiro, L., Novo, M. & Al-Soufi, W. (2015) Fluorescence emission of pyrene in surfactant solutions. *Adv. Colloid Interface Sci.* 215, 1–12. doi: 10.1016/j.cis.2014.10.010
